# Canfam_GSD*: De novo* chromosome-length genome assembly of the German Shepherd Dog (*Canis lupus familiaris*) using a combination of long reads, optical mapping, and Hi-C

**DOI:** 10.1093/gigascience/giaa027

**Published:** 2020-04-01

**Authors:** Matt A Field, Benjamin D Rosen, Olga Dudchenko, Eva K F Chan, Andre E Minoche, Richard J Edwards, Kirston Barton, Ruth J Lyons, Daniel Enosi Tuipulotu, Vanessa M Hayes, Arina D. Omer, Zane Colaric, Jens Keilwagen, Ksenia Skvortsova, Ozren Bogdanovic, Martin A Smith, Erez Lieberman Aiden, Timothy P L Smith, Robert A Zammit, J William O Ballard

**Affiliations:** 1 Centre for Tropical Bioinformatics and Molecular Biology, Australian Institute of Tropical Health and Medicine, James Cook University, Smithfield Road, Cairns, QLD 4878, Australia; 2 John Curtin School of Medical Research, Australian National University, Garran Rd, Canberra, ACT 2600, Australia; 3 Animal Genomics and Improvement Laboratory, Agricultural Research Service USDA, Baltimore Ave, Beltsville, MD 20705, USA; 4 The Center for Genome Architecture, Department of Molecular and Human Genetics, Baylor College of Medicine, Baylor Plaza, Houston, TX 77030, USA; 5 Department of Computer Science, Rice University, Main St, Houston, TX 77005, USA; 6 Center for Theoretical and Biological Physics, Rice University, Main St, Houston, TX 77005, USA; 7 Garvan Institute of Medical Research, Victoria Street, Darlinghurst, NSW 2010, Australia; 8 St Vincent’s Clinical School, University of New South Wales Sydney, Victoria Street, Darlinghurst NSW 2010, Australia; 9 Faculty of Medicine, UNSW Sydney, High St, Kensington, NSW 2052, Australia; 10 School of Biotechnology and Biomolecular Sciences, UNSW Sydney, High St, Kensington, NSW 2052, Australia; 11 Central Clinical School, University of Sydney, Parramatta Road, Camperdown, NSW 2050, Australia; 12 Julius Kühn-Institut, Erwin-Baur-Str. 27, 06484 Quedlinburg, Germany; 13 Broad Institute of MIT and Harvard, Main St, Cambridge, MA 02142, USA; 14 Shanghai Institute for Advanced Immunochemical Studies, ShanghaiTech University, ShanghaiTech University, Huaxia Middle Rd, Pudong 201210, China; 15 US Meat Animal Research Center, Agricultural Research Service USDA, Rd 313, Clay Center, NE 68933, USA; 16 Vineyard Veterinary Hospital, Windsor Rd, Vineyard, NSW 2765, Australia

**Keywords:** Hi-C, long-read sequencing, optical mapping, *de novo* genome assembly, canine hip dysplasia, DNA Zoo

## Abstract

**Background:**

The German Shepherd Dog (GSD) is one of the most common breeds on earth and has been bred for its utility and intelligence. It is often first choice for police and military work, as well as protection, disability assistance, and search-and-rescue. Yet, GSDs are well known to be susceptible to a range of genetic diseases that can interfere with their training. Such diseases are of particular concern when they occur later in life, and fully trained animals are not able to continue their duties.

**Findings:**

Here, we provide the draft genome sequence of a healthy German Shepherd female as a reference for future disease and evolutionary studies. We generated this improved canid reference genome (CanFam_GSD) utilizing a combination of Pacific Bioscience, Oxford Nanopore, 10X Genomics, Bionano, and Hi-C technologies. The GSD assembly is ∼80 times as contiguous as the current canid reference genome (20.9 vs 0.267 Mb contig N50), containing far fewer gaps (306 vs 23,876) and fewer scaffolds (429 vs 3,310) than the current canid reference genome CanFamv3.1. Two chromosomes (4 and 35) are assembled into single scaffolds with no gaps. BUSCO analyses of the genome assembly results show that 93.0% of the conserved single-copy genes are complete in the GSD assembly compared with 92.2% for CanFam v3.1. Homology-based gene annotation increases this value to ∼99%. Detailed examination of the evolutionarily important pancreatic amylase region reveals that there are most likely 7 copies of the gene, indicative of a duplication of 4 ancestral copies and the disruption of 1 copy.

**Conclusions:**

GSD genome assembly and annotation were produced with major improvement in completeness, continuity, and quality over the existing canid reference. This resource will enable further research related to canine diseases, the evolutionary relationships of canids, and other aspects of canid biology.

## Introduction

Arising from wild grey wolves on the Eurasian continent >15,000 years ago, the dog (*Canis lupus familiaris*, NCBI:txid9615) was the first species to be domesticated [1–3]. Mitochondrial DNA evidence suggests that seats of canine domestication may have been China [3], Europe [4], and the Middle East [5]. Since domestication, canids have undergone thousands of years of selective breeding, giving rise to a myriad of phenotypic variants. However, most modern breeds are <200 years old and are of European ancestry [6, 7].

The German Shepherd Dog (GSD) is a medium to large working dog and was developed from common livestock dogs late in the 19th century in continental Europe [7]. In 1899, Captain Max von Stephanitz attended a dog exhibition event and was shown a dog named "Hektor Linksrhein." Hektor satisfied what von Stephanitz believed a working dog should be, and he bought him immediately. After purchasing the dog, von Stephanitz changed his name to "Horand von Grafrath" and founded the Verein für Deutsche Schäferhunde (Society for the German Shepherd Dog). Horand was declared to be the first GSD and was the first dog added to the society's breed register [8]. Von Stephanitz is reported to have kept a strong reign over the early development of the GSD, and this likely resulted in a degree of inbreeding. However, it also enabled the fixation of qualities that are now features of the breed.

Subsequent roles for the GSD, which included guarding and police work, contributed to selective breeding for larger and more confident dogs [9]. Over recent decades, further selection towards characteristics deemed desirable in the show ring have further altered the GSD conformation [10]. Perhaps the best-known disease is canine hip dysplasia (CHD), which is a complex disease combining genetic and environmental factors. Genetic factors, such as shallow acetabulum, subluxation, and poorly forming femoral heads will manifest early in a dog's life if severe. Environmental factors such as overweight or poor exercise area (many stairs and much jumping in juvenile life) will manifest in later life. Other common health problems include elbow dysplasia, bloat, degenerative myelopathy, epilepsy, haemophilia, diabetes, inflammatory bowel disease, and a variety of cancers including osteosarcoma, lymphoma, and melanoma [11–16].

In Australia, early imports of GSDs were known to have arrived from 1904. In October 1928, the Federal Government of Australia placed an importation ban on the breed, which was enforced in 1929. During the course of the import ban, which was to stretch for another 43 years, few imports were smuggled into the country. The import ban was lifted in 1972, with some restrictions remaining until 1976. With the lifting of the import ban, German, New Zealand, English and some American dogs were imported into Australia, and the breed enjoyed a surge in popularity. Currently, the GSD is the largest breed (purebred) dog population in Australia [17].

**Figure 1:**
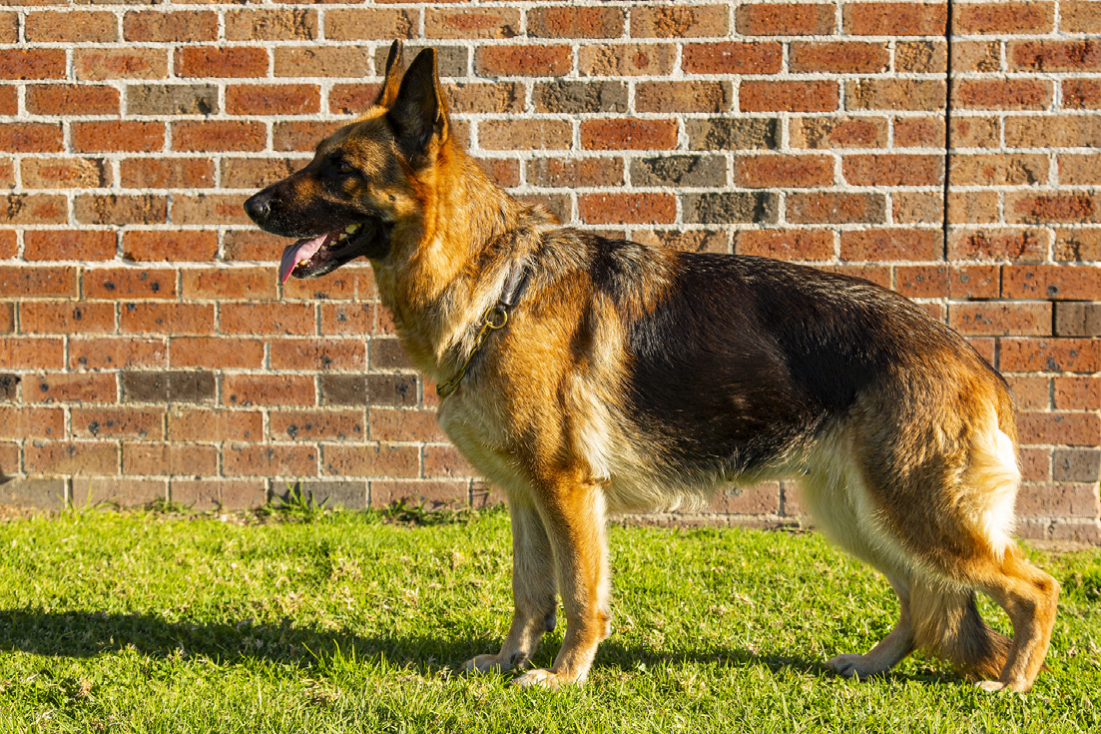
"Nala" the female German Shepherd Dog. Nala, or formally “Jonkahra Nala” (Australian Registration #2100398550), was born in 2013 and is free of all known genetic diseases. Her sire was imported from Germany, and her dam is from Australian lines.

The aim of this study is to is to provide a high-resolution long-read *de novo* assembly of the genome of a GSD female that is free of known genetic diseases (Fig. 1). This *de novo* genome assembly will be an invaluable tool for advancing knowledge of both simple and polygenic genetic diseases and also the evolutionary affinities of the GSD.

## Results

### Workflow

The genome was assembled using Pacific Bioscience (PacBio) Single Molecule Real-Time (SMRT) sequencing, Oxford Nanopore (ONT) PromethION sequencing, 10X Genomics Chromium genome sequencing with Bionano, and Hi-C scaffolding (Supplementary Fig. 1). Contigs were assembled using SMRT and ONT sequencing [18] and then polished [19, 20] to minimize error propagation (see Long read genome assembly section for details). The assembled sequence contigs were scaffolded sequentially using 10X linked reads, Bionano optical mapping, and Hi-C proximity ligation scaffolding. To increase the contiguity of the assembly we used the SMRT and ONT reads to fill gaps, which was then followed by a final round of polishing. Homology-based gene prediction was performed using *C. lupus familiaris* and 8 related mammals. The resulting chromosome-length genome assembly and its gene annotation was deposited to NCBI with accession number GCA 008641055.2. The mitochondrial genome (VSDE01000430) was subsequently added to the assembly. Finally, comparisons with the canine genome of the boxer (CanFam3.1) were made [21].

### Assembly statistics/completeness

The final submission contains 2,407,291,559 total bp (2,401,147,102 ungapped), 429 scaffolds with a contig N50 length of 20.9 Mb, and a scaffold N50 length of 64.3 Mb. The full-length chromosome scaffolds in the assembly accounted for 98.3% of the genome, with only 0.95% of all sequence not aligning to a CanFam3.1 chromosome. Evaluation by BUSCO (BUSCO v3.0.2b [22], short mode, implementing BLAST+ v2.2.31 [23], HMMer v3.2.1 [24], AUGUSTUS v3.3.2 [25], and EMBOSS v6.6.0) against the Laurasiatheria_ob9 dataset (n = 6,253) indicated that 93.0% of the conserved single-copy genes were complete (Table 1, Supplementary Tables 1, 2, Supplementary Fig. 2). Each analysis step in assembly, scaffolding, and polishing improved scaffold N50 and/or BUSCO scores, consistent with improving assembly quality (Supplementary Table 1, Supplementary Fig. 2). BUSCO predictions are sensitive to changes in sequence and assembly size, with scaffolding and polishing causing losses as well as gains (Supplementary Table 1). Compiling BUSCO results across all assembly stages (BUSCOMP v0.8.0) reveals that ≥6,085 (97.3%) are present and complete in the assembly, with only 118 genes (1.9%) not found at any stage.

**Table 1:** Genome assembly and annotation statistics for GSD assembly vs CanFam3.1

Statistic	GSD	CanFam3.1
Total sequence length	2,407,291,559	2,410,976,875
Total ungapped length	2,401,147,102	2,392,715,236
No. of contigs	735	27,106
Contig N50	20,914,347	267,478
Contig L50	37	2,436
No. of scaffolds	410	3,268
Scaffold N50	64,346,267	63,241,923
Scaffold L50	15	15
No. of gaps	306	23,876
BUSCO complete (genome)	93.0% (91.6% single copy, 1.4% duplicate copy)	92.2% (91.1% single copy, 1.1% duplicate copy)
BUSCO fragmented (genome)	3.6%	4.0%
BUSCO missing (genome)	3.4%	3.8%
BUSCO complete (annotation)	98.9% (96.5% single copy, 2.4% duplicate copy)	95.1% (94.1% single copy, 1.0% duplicate copy)
BUSCO fragmented (annotation)	1.0%	1.9%
BUSCO missing (annotation)	0.1%	3.0%

Additional *k*-mer analysis of the final assembly was performed using KAT v2.4.2 [26]. KAT comp was used to compare *k*-mer frequencies from the 10X reads (16 bp barcode trimmed from read 1) with their copy number in the assembly. This comparison revealed no sign of missing data nor large duplications, including retention of haplotigs (Supplementary Fig. 3).

### Comparison with CanFam3.1

The GSD assembly was compared with the current reference genome CanFam3.1. Results are summarized in Table 1.

The GSD assembly offers improvements over CanFam3.1 using a wide variety of metrics. The GSD assembly has a contig N50 that is almost 80 times greater than CanFam3.1 and contains 78 times fewer gaps, and 2,881 fewer scaffolds. BUSCO results on the genome also indicate an improvement in the GSD assembly, with 47 more complete genes (25 fewer fragmented genes and 22 fewer missing genes).

On the basis of the existing CanFam3.1 annotation and the GSD annotation provided by GeMoMa [27], the longest full-length transcript per gene was selected to avoid an overestimation of duplicated genes by BUSCO v3.02. Comparing the BUSCO statistics for the annotations, a clear improvement from 95.1% to 98.9% complete single-copy orthologs could be observed.

#### Variation relative to CanFam3.1

All 39 full-length chromosomes in the final assembly were aligned to the corresponding chromosomes in CanFam3.1 using MUMmer4 [28]. Single-nucleotide polymorphisms (SNPs) and small indels (deletions and insertions <50 bp) were called using the MUMmer4 call-SNPs module. In total 3,137,227 SNVs and 5,111,356 small indels were detected (Supplementary Table 3). Copy number variants (CNVs) and structural variants (SVs) were called using svmu (v0.2) [29]. Variants >100 bp were extracted, resulting in 66,673 total CNV/SVs. By variant type, this was broken down into 39,742 CNVs, 13,552 insertions, 13,150 deletions, and 229 inversions (Supplementary Table 4).

#### Pancreatic amylase (AMY2B) analysis

**Figure 2:**
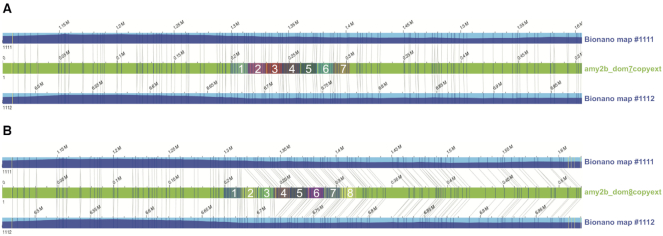
Bionano genome map alleles aligned to hypothetical sequence constructs. The hypothetical sequence constructs (green bars) contain either 7 (labelled "amy2b_dom7copyext") or 8 (labelled "amy2b_dom8copyext") copies of the repeat unit (highlighted by coloured boxes within the green bar and numbered in white font). Dark blue and yellow vertical lines on the sequence contig and consensus map indicate matching and non-matching DLE1 enzymatic labels, respectively.

*AMY2B* is important in canid evolution, with variation in copy number being linked to starch diet adaptations in ancient European dogs. Ollivier et al. looked at both ancient and modern dogs, finding the expansion as early as the seventh century, with between 4 and 16 copies in modern dogs [30]. No long reads were found to span the entire region. The longest read in the region covered ≤3 complete copies, with 4 copies ultimately submitted in the GSD assembly. Further examination of this region was attempted using both the Bionano optical map and read depth analysis from the SMRT and ONT reads (Supplementary File 1). The read depth results estimate that there are between 7 and 8 copies of the gene, while the Bionano map indicated that the most likely copy number is 7 (Fig. 2). For the Bionano analysis, single molecules of Bionano data were *de novo* assembled using a haplotype-aware algorithm (Supplementary File 2) to obtain a phased consensus genome map set. Alignment of the resulting genome maps to the GSD assembly identified 2 homozygous alleles (Map ID Nos. 1111 and 1112) spanning the *AMY2B* region as predicted by GeMoMa (Supplementary Fig. 4). The alignment shows a ∼11 kb “insertion,” flanked by DLE1 enzymatic labels at positions 47,325,815 and 47,333,432 of NALACHR6.01, suggesting that this fragment, which is upstream of the *AMY2B* region, is either lost or collapsed in the GSD assembly. Additionally, the region flanked by DLE1 labels at 47,341,704 and 47,396,280, which encompasses 3 of the GeMoMa-predicted *AMY2B* copies, is tandemly duplicated, suggesting 7 possible copies of *AMY2B* in Nala. The 2 alleles are supported by an average of 40× and 23× single long molecules, with 12 spanning the full repeat structure, of which 8 also span the 11-kb insertion. It should be noted here that, owing to sequence similarity between the 4 GeMoMa-predicted *AMY2B* copies and associated inherent alignment ambiguities, it is unclear exactly which repeat units are duplicated.

Compared with CanFam3.1, the pair of homozygous genome map alleles show an insertion of ∼100 kb flanked by DLE1 labels at positions 46,954,644 and 46,999,962 of Chr6 (Supplementary Fig. 5), which is indicative of a complete loss of the *AMY2B* locus in CanFam3.1.

To better determine whether Bionano data support 7 or 8 copies of the *AMY2B* repeat, we compared the 2 genome map alleles against 2 synthetic sequence constructs containing either 7 (amy2b_dom7copyext) or 8 (amy2b_dom8copyext) copies of the 14,862-bp *AMY2B* repeat with the highest read depth support (namely, the third copy) from the GSD assembly, flanked by ∼401.5-kb sequences assembled from SMRT and ONT reads (Supplementary File 1). Alignment results confirmed the presence of 7 repeat units, showing a perfect alignment to the 7-copy sequence construct (Fig. 2A), but a “deletion” of 1 repeat unit relative to the 8-copy construct (Fig. 2B).

#### Olfactory receptor and keratin cluster analyses

Correct annotation of the olfactory and keratin clusters in canines has been problematic, but it is important for research on canid health and evolution [21, 31, 32]. Dogs are macrosmatic animals that rely highly on their sense of smell. Yet, the molecular basis of such prominent chemosensory capacities remains largely unknown. The ability to detect and discriminate the multitude of odors in vertebrates is mediated by a superfamily of G protein–coupled olfactory receptor (OR) proteins [33]. Based on the description in the reference annotations, we filtered all messenger RNAs (mRNAs) of the references that contain in their description the regular expression “olfactory receptor.” We then extracted the number of mRNAs and genes per reference organism. Subsequently, we used the IDs to filter the GSD annotation and counted the number of predicted mRNAs and genes. This procedure identifies 1,250 mRNAs and 933 genes in the GSD and 849 mRNAs and 804 genes in the boxer. Quignon et al. [31] identified 5 amino acid patterns characteristic of ORs in the canine genome and retrieved 1,094 dog genes (872 genes and 222 pseudogenes).

Keratins are filament proteins of the epithelial cytoskeleton and are essential for normal skin homeostasis. Over time the genes encoding keratins have undergone multiple rounds of duplication, with high similarity between different keratin paralogs [32]. Analogously to the olfactory receptor study, we filtered all mRNAs of the references that contain in their description the keyword “keratin\d.” This procedure identifies 118 mRNAs and 83 genes in the GSD and 73 mRNAs and 55 genes in the boxer. Balmer et al. [32] investigated the NCBI (dog annotation release 103) gene predictions for the canine gene clusters to RNA-sequencing data that were generated from adult skin of 5 dogs and adult hair follicle tissue of 1 dog and annotated 61 putatively functional keratin genes in the dog.

## Discussion

Concerns for the health welfare of the GSD have been widely aired [34, 35]. The GSD had the highest number of published predispositions to inherited diseases overall among the 50 most commonly registered Kennel Club breeds and had the second-highest number of disorders exacerbated by conformation, exceeded only by the Great Dane [36]. The British Kennel Club Breed Watch system categorizes the GSD as a Category Three breed “requiring particular monitoring and additional support” and considered to be more susceptible to developing specific health conditions associated with exaggerated conformation. Breed Watch points of concern include cow hocks, excessive turn of stifle, nervous temperament, sickle hock, and weak hindquarters [37].

The high-quality genome assembly will advance knowledge of breed-specific diseases such as CHD and extend to issues related to canine personality. The severity of CHD depends on both genetic and environmental factors. In GSDs, the heritability (*h*^2^) estimates have varied from 0.1 to 0.6 [38]. To date, different study populations and methods affect the results substantially because the reported quantitative trait locus association and candidate genes are inconsistent between studies [39–41]. While boxers are prone to CHD, the hip scores of "Tasha" (used for CanFam) are unknown. Furthermore, GSD-specific SNPs as well as significant CNVs and SVs are difficult to detect. Concerning canine personality, Saetre et al. [42] examined how traits are transmitted between generations in a cohort containing >10,000 behaviorally tested GSD and Rottweiler dogs. In both breeds, the pattern of co-inheritance was found to be similar for a broad personality trait previously named shyness–boldness, with heritability estimated to be 0.25 in the 2 breeds. Currently, the underlying genes involved in these behaviors are not known.

The assembly is expected to enable the selection of GSDs for particular duties including police work, where their sensitive nose is frequently used to discriminate odors. Robin et al. [43] analysed the nucleotide sequences of 109 OR genes (102 genes and 7 pseudogenes) in 6 different breeds including GSDs. In this study, they showed that OR genes are highly polymorphic, with a mean of 1 SNP per 577 nucleotides. However, the degree of polymorphism observed is highly variable, with some OR genes having few if any SNPs and others being highly polymorphic (1 SNP/122 nucleotides). Yang et al. [44] conducted a preliminary study of 22 SNPs from the exonic regions of 12 OR genes in GSDs and found a significant correlation between SNP genotypes of OR genes and olfactory abilities of dogs.

We envisage that these data will also facilitate understanding of the evolution of dog breeds and canids in general. The evolutionary position of the GSD among extant breeds is not firmly established. The Fédération Cynologique Internationale places it in Group 1 as part of the Herding group. Bigi et al. [45] hypothesized that the German Shepherd dog was closely related to the Czechoslovakian wolfdog. More recently Parker et al. [6] proposed that the GSD is distinct from other herding breeds and in a clade along with the French Berger Picard, New Hampshire Chinook, Peruvian hairless, and Mexican xoloitzcuinti.

## Conclusions

This *de novo* genome assembly and annotation will be an invaluable tool for advancing knowledge of breed-specific diseases and the evolutionary affinities of the GSD. Here, we present an improved canid genome assembly and annotation relative to CanFam 3.1.

## Methods

### DNA extraction, sequencing, and scaffolding

#### Sampling: Nala the German Shepherd Dog

In selecting an animal for the project, it was considered essential to select a female that had been cleared, as much as possible, of any recognizable inherited conditions. The animal needed to display all the hallmarks of a good quality representative of the breed but need not necessarily be a show-winning specimen. Nala is an easygoing and approachable 5.5-year-old female (born 5 December 2013) and a treasured family pet that showed typical appearance for a GSD. She has had no sign of the hip dysplasia that appears in GSD (Supplementary Fig. 6) or any other known genetic diseases. Nala had a combined hip score of 3 (1 on the left-hand side and 2 on the right-hand side) when the X-ray was taken at 5 years of age: each hip was measured on a 0–53 scale, with a total of 106 being crippling. The score of 3 is well below the current Australian average of 9 for GSDs. She is registered with the Australian National Kennel Council (# 2100398550) with her dam from Australian bred lines and sire imported from Germany. Her dam and sire remain healthy aging adults without disease. Nala's dam has 7 progeny radiographed from 4 sires with no failures. Her sire had 31 progeny radiographed from 13 different dames resulting in 4 failures and 27 passes recorded for the GSD National Council hip scheme. In the Australian 53-point scoring scheme, a pass is ≤8 in any 1 hip, no point gets a 3, and ≤16 in total.

#### Pacific Biosciences SMRT sequencing

Genomic DNA was prepared from 1–2 mL of fresh blood using the genomic-tip 100/G kit (Qiagen, Hilden, Germany). This was performed with supplemental RNase (Astral Scientific, Taren Point, Australia) and proteinase K (NEB, Ipswich, MA, USA) treatment, as per the manufacturer's instructions. Isolated genomic DNA was further purified using AMPure XP beads (Beckman Coulter, Brea, CA, USA) to eliminate sequencing inhibitors. DNA purity was calculated using a Nanodrop spectrophotometer (Thermo Fisher Scientific, Waltham, MA, USA), and molecular integrity was assessed using pulse-field gel electrophoresis. DNA integrity was assessed by the Sage Science Pippin Pulse. A 0.75% KBB (Sage Science, Beverly, MA, USA) gel was run on the 9hr 10–48 kb (80 V) program. The DNA ladder used was the Invitrogen 1 kb Extension DNA ladder (cat No. 10,511–012). A total of 150 ng of DNA was loaded on the gel.

We generated 2 libraries that were size selected on Sage BluePippin gels (Sage Science, Beverly, MA, USA). Libraries were sequenced on Sequel machines with 2.0 chemistry recording 10 h movies (PacBio Sequel System, RRID:SCR_017989). Sequencing was conducted at the Ramaciotti Center for Comparative Genomics at University of New South Wales (TOW5157A1, 15 SMRT cells with a total polymerase read length 108.48 Gb) and at the Arizona Genomic Institute, University of Arizona (4 SMRT cells with a total of 11 Gb of data; note: short-read lengths were due to DNA shearing of the DNA during shipping from Australia to Arizona).

#### ONT PromethION sequencing

DNA (1 µg) was prepared for ONT sequencing using the 1D genomic DNA by ligation kit (SQK-LSK109, ONT) according to the standard protocol. Long fragment buffer was used for the final elution to exclude fragments shorter than 1,000 bp. In total, 119 ng of adapted DNA was loaded onto a FLO-PRO002 PromethION flow cell and run on an ONT PromethION sequencing device (PromethION, RRID:SCR_017987) using MinKNOW (18.08.2) with MinKNOW core (v1. 14.2).

Base-calling was performed after sequencing with the GPU-enabled guppy basecaller (v3.0.3) using the PromethION high-accuracy flip-flop model with config “dna_r9.4.1_450bps_hac.cfg.”

#### 10X Genomics Chromium sequencing

DNA was prepared following the protocol described above for SMRT sequencing. A 10X GEM library was barcoded from high molecular weight (HMW) DNA according to the manufacturer's recommended protocols. The protocol used was the Chromium Genome Reagent Kits v2 User Guide, manual part No. CG00043 Rev B [46]. Quality control was performed using LabChip GX (PerkinElmer, Waltham, MA, USA ) and Qubit 2.0 Flurometer (Life Technologies, Carlsbad, CA, USA) at the Kinghorn Centre for Clinical Genomics. The library was run on a single lane of a v2 patterned flowcell. Paired-end sequencing with 150- bp read length was performed using the Illumina HiSeq X (Illumina HiSeq X Ten, RRID:SCR_016385) within the Kinghorn Centre for Clinical Genomics at the Garvan Institute of Medical Research, Sydney, Australia.

#### DNA methylome

To explore the regulatory landscape of the GSD, we performed whole-genome bisulfite sequencing [47] on genomic DNA extracted from whole blood. In concordance with other adult vertebrates [48, 49], the GSD genome displays a typical bimodal DNA methylation pattern with >60% of CpG dinucleotides being methylated at levels >80% (hypermethylated) and 12% of CpG dinucleotides being methylated at ≤20% (hypomethylated). Next, to determine the number and genomic distribution of putative regulatory regions, we segmented the methylome into unmethylated regions (UMRs) and low-methylated regions (LMRs), using the MethylSeekR algorithm [50]. UMRs are fully unmethylated and largely coincide with CpG island promoters whereas LMRs display partial DNA methylation, which is characteristic of distal regulatory elements such as enhancers in other mammalian models [51]. These analyses resulted in the identification of ∼21,000 UMRs and ∼53,000 LMRs, in line with previously reported numbers of promoters and enhancers [50, 52] (Supplementary Fig. 7).

#### Bionano optical mapping

HMW DNA was isolated from fresh blood (stored at 4°C) using the Bionano Prep Blood DNA Isolation Protocol (Bionano Genomics [BNG], Document #30,033 revision C). Briefly, after lysing the red blood cells, white blood cells were recovered and embedded in agarose plugs. These plugs were subjected to Proteinase K (Qiagen Cat No. 158,920) digestion for 2 rounds (2 hours, then overnight) at 50°C. Following extensive washing as prescribed in the protocol, the plugs were melted and treated with GELase enzyme (Epicentre, Cat. No. G31200). The resulting HMW DNA was subjected to drop dialysis, left to equilibrate at room temperature for 4 days, and was then quantified using the Qubit Broad Range dsDNA Assay Kit (Thermo Fisher Scientific).

HMW DNA (∼190 ng/µL) was labelled (BNG, Part No. 20,351) at DLE-1 recognition sites, following the Bionano PrepTM Direct Label and Stain Protocol (BNG, Document No. 30,206 revision C). Labelled DNA was loaded directly onto Bionano Saphyr Chips (BNG, Part No. 20,319), without further fragmentation or amplification, and imaged using a Saphyr instrument to generate single-molecule optical maps (Saphyr, RRID:SCR_017992). Multiple cycles were performed to reach an average raw genome depth of coverage of 190×.

#### Hi-C chromosome length scaffolding

The Bionano assembly was further scaffolded to chromosome length by the DNA Zoo following the prescribed methodology [53]. Briefly, an *in situ* Hi-C library was prepared [54] from a blood sample of a purebred male individual named Tydus (American Kennel Club Registration DN5364660) provided by the Cornell Veterinary Biobank and sequenced to 29× coverage (assuming 2.4-Gb genome size).

## Genome Assembly Workflow

### Long-read genome assembly

The SMRT and ONT reads were corrected and assembled with the Canu assembler (Canu, RRID:SCR_015880) v1.8.0 [18]. The resulting contigs were polished by aligning the raw reads to the assembly and correcting the sequencing errors using 2 rounds of Arrow polishing [19]. There were ∼10 million fixes in the first round and ∼284,000 fixes in the second. The assembled GSD genome, with a total length of 2.39 Gb, consisted of 1,389 contigs with an N50 length of 15.68 Mb. Following the Arrow polishing there were 1,389 sequences, with a total length of 2.39 Gb (including 111 repeats of total length 13,145,025 bp) with no bubbles. There were 2,560,498 unassembled sequences of total length 17,998,063,955 bp.

### 10X Chromium linked reads

The Arrow-polished SMRT/ONT assembly was scaffolded using GSD 10X linked reads as in ARCS [55]. The 10X data were aligned using the linked-read analysis software provided by 10X Genomics, Long Ranger, v2.1.6 [56]. Misaligned reads and reads not mapping to contig ends were removed, and all possible connections between contigs were computed keeping best reciprocal connections. Finally, contig sequences were joined, spaced by 10 kb with stretches of N's, and if required reverse complemented (Supplementary File 3). In total 128 connections between the SMRT/ONT contigs could be established, increasing the assembly N50 length by 4.6 Mb (from 15.46 to 20.06 Mb; Supplementary File 3).

### Polishing round 1

To further improve the assembly, another round of polishing was performed by aligning the Illumina short reads from the 10X Chromium sequencing to the assembly using minimap2 [57] (v2.16) and correcting the sequencing errors using Racon (Racon, RRID:SCR_017642) v1.3.3 [58].

### Optical mapping for super-scaffolding using Bionano data

Single-molecule optical maps were filtered on minimum molecule length of 150 kb and minimum of 9 label sites per molecule. *De novo* assembly of single molecules into consensus maps was performed using the Bionano Solve (v3.2.2_08022018) software with aligner RefAligner (7782.7865rel) [59, 60]. Assembly was “haplotype-unaware” such that heterozygous alleles were collapsed into haploid representation. In all, ∼2 million single molecules with N50 of 220 kb were assembled into 1,245 optical genome maps with N50 of 3.1 Mb. The final assembly was in CMAP format (v0.2).

This genome map set was used to scaffold the sequence contigs using BNG's Hybrid Scaffold pipeline (v10252018). In brief, the 1,261 sequence contigs were *in silico* digested on the basis of the DLE-1 motif (CTTAAG) creating sequence maps (CMAP). Sequence maps were then aligned to the assembled optical maps based on DLE-1 labels using RefAligner. Discrete sequence maps that can be linked via a Bionano genome map were scaffolded.

Alignments indicating conflict between the sequence and optical maps, and hence suggestive of misassembly, were resolved. Specifically, optical maps supported by ≥10 single molecules at the conflict site were indicative of sequence misassembly, and so the sequence map would be “cut” (split) at the conflict point. In contrast, insufficient single-molecule support for the optical map was indicative of optical map assembly error, and so the optical map would be “cut” at the conflict site. Details of the method are provided in the Bionano Solve Theory of Operation: Hybrid Scaffold (Document No. 30,073). Following hybrid scaffolding, 21 arbitrary 10-kb N-gaps (introduced during the sequence assembly process) were resized on the basis of estimated inter-label distances from the optical maps. In all, 160 sequence contigs were hybrid-scaffolded into 109 hybrid scaffolds with N50 of ∼46.3 Mb. The remaining 1,004 sequence contigs with an N50 of ∼78.8 kb could not be scaffolded either because they were too short (<100 kb) for hybrid scaffolding with Bionano maps or because they did not align to any optical maps.

### Chromosome-length assembly using Hi-C data

**Figure 3:**
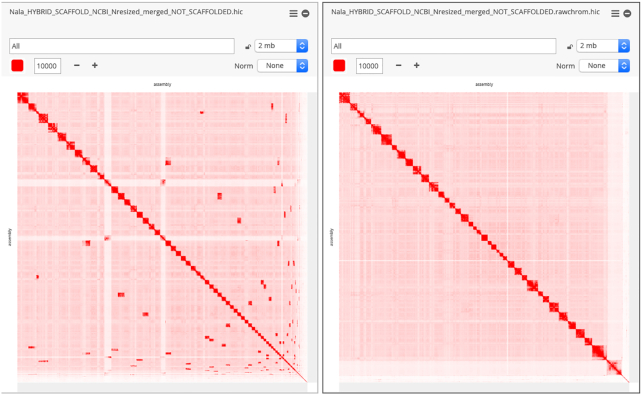
GSD assembly before and after Hi-C correction. Contact matrices (visualized in Juicebox.js) comparing the GSD assembly before and after the chromosome-length Hi-C upgrade.

The Hi-C data were processed using Juicer (Juicer, RRID:SCR_017226) [61] and used as input into the 3D-DNA pipeline [62] to produce a candidate chromosome-length genome assembly. We performed additional finishing on the scaffolds using Juicebox Assembly Tools [63]. Fig. 3 shows the contact matrices generated by aligning the Hi-C dataset to the genome assembly before the Hi-C upgrade (left) and after Hi-C scaffolding (right). The matrices are visualized in Juicebox.js, a cloud-based visualization system for Hi-C data [64], and are available for browsing at multiple resolutions at DNA Zoo [65].

### Gap filling

After scaffolding and correction, all raw SMRT and ONT reads were aligned to the assembly with Minimap2 (v2.16) (-ax map-pb/map-ont) and used by PBJelly (pbsuite v.15.8.24) [66] to fill gaps. It was able to completely close 210 gaps, increasing contig N50 to the final figure of 20.9 Mb.

### Polishing round 2

Following scaffolding, another round of polishing was done to further improve the assembly. Polishing was performed by aligning the Illumina short reads from the Chromium sequencing to the assembly using Long Ranger v2.2.2 and correcting the SNPs and indels using Pilon (Pilon, RRID:SCR_014731) [20].

### Final cleanup

The Pilon-polished genome underwent a final scaffold cleanup to generate a high-quality core assembly, remove low-coverage artefacts and haplotig sequences, and annotate remaining scaffolds with potential issues.

### Low-coverage filter

The TOW5157A1 library PacBio subreads (12.5 M subreads; 108 Gb) were mapped onto the Nala_canu_arrow2_10x_racon_bionano_HiC_pbjelly_pilon assembly using Minimap2 v2.16 (-ax map-pb –secondary = no) [57]. Initial read depth analysis was performed with BBMap v38.51 pileup.sh [67]. Any scaffolds with median coverage <3 (e.g., <50% of the scaffold covered by ≥3 reads) were filtered out as low-coverage scaffolds. Of the 1,057 Pilon-polished scaffolds, 220 scaffolds were removed in the initial low-coverage filter, leaving 837 scaffolds.

### Purge Haplotigs analysis—round 1

Subreads were remapped on the remaining 837 scaffolds and processed with PurgeHaplotigs v20190612 [68] (implementing Perl v5.28.0, BEDTools v2.27.1 [69], R v3.5.3, and SAMTools v1.9 [70]). Based on the PurgeHaplotigs depth histogram, low-, mid- and high-depth thresholds were set to 5×, 30×, and 80×. Any scaffolds with <80% at diploid read depth were identified by PurgeHaplotigs for reassignment. Scaffolds with ≥80% bases in the low/haploid coverage bins and ≥95% of their length mapped by PurgeHaplotigs onto another scaffold were filtered as haplotigs or assembly artefacts. Any other scaffolds with ≥80% low-coverage bases were filtered as "low coverage." This analysis resulted in a further 11 scaffolds filtered for low coverage and 268 filtered as haplotigs or assembly artefacts, leaving 558 scaffolds.

### Purge Haplotigs analysis—round 2

Subreads were remapped onto the remaining 558 scaffolds, resulting in a further 128 scaffolds filtered as haplotigs or assembly artefacts, leaving 430 scaffolds. No additional scaffolds with ≥80% low-coverage bases were identified. Any scaffold with ≥80% bases in the low/haploid coverage bins were filtered as haplotigs or assembly artefacts. Scaffolds with ≥20% diploid coverage were marked as retention as probable diploids. Scaffolds with <20% diploid coverage and ≥50% high coverage were marked as probable collapsed repeats. A single remaining scaffold marked as "junk" by PurgeHaplotigs (>80% low/high coverage) was also filtered as a probable artefact.

### Purge Haplotigs analysis—round 3

Subreads were remapped onto the remaining 430 scaffolds for a third round of PurgeHaplotigs analysis. No further scaffolds were identified for filtering.

### CanFam3.1 chromosome mapping

The CanFam v3.1 reference genome was downloaded from Ensembl (Release 97, download date 5 August 2019). Full-length chromosomes were renamed with a CANFAMCHR prefix and used for reference mapping. The final Nala genome assembly was mapped onto the CanFam3.1 reference genome using Minimap2 v2.16 [57] (-x asm5 –secondary = no –cs) to generate PAF output. Scaffolds were assigned to CanFam3.1 chromosomes using PAFScaff v0.2.0 (PAFScaff, RRID:SCR_017976) [71] based on Minimap2-aligned assembly scaffold coverage against the reference chromosomes. Scaffolds were assigned to the chromosome with highest total coverage. Scaffolds failing to map onto a chromosome were rated as “unplaced.”

### Final scaffold classification

Subreads were remapped onto the renamed and reoriented scaffolds for a final round of PurgeHaplotigs analysis to classify scaffolds that may have escaped filtering or have unusual read depth profiles. Scaffolds were placed into 1 of 5 categories:

1. DIPLOID (core) scaffolds have <50% match to another Scaffold and the dominant PurgeHaplotigs coverage bin is Diploid depth
2. REPEAT scaffolds have >50% match to another Scaffold and the dominant PurgeHaplotigs coverage bin is Diploid depth
3. COLLAPSED_REPEAT scaffolds have high coverage PurgeHaplotigs bin dominant
4. HAPLOID regions have ≥50% match to another Scaffold and the dominant PurgeHaplotigs coverage bin is Haploid depth, but filtering criteria were not met
5. LOWQUALITY scaffolds have ≥50% match to another Scaffold and the dominant PurgeHaplotigs coverage bin is low coverage depth, but filtering criteria were not met

Finally, 20 REPEAT scaffolds corresponding to a PacBio control sequence were removed from the assembly, leaving the final 409 nuclear scaffolds plus mitochondrion. Seventeen scaffolds had small regions masked or trimmed by the NCBI Contamination screen, corresponding to a 3.4-kb chunk of *Escherichia coli*.

### Gene prediction including annotation of repetitive elements

The genome was annotated using the homology-based gene prediction program GeMoMa (GeMoMa, RRID:SCR_017646) v1.6.2beta [27] and 9 reference organisms. The 9 species used for the homology-based gene prediction analyses were *C. lupus familiaris* (CanFam3.1; GCF_000002285.3), *Vulpes vulpes* (VulVul2.2; GCF_003160815.1), *Felis catus* (Felis_catus_9.0; GCF_000181335.3), *Sus scrof* (Sscrofa11.1; GCF_000003025.6), *Bos taurus* (ARS-UCD1.2; GCF_002263795.1), *Ailuropoda melanoleuca* (ASM200744v1; GCF_000004335.2), *Ursus maritimus* (UrsMar_1.0; GCA_000687225.1), *Mus musculus* (GRCm38.p6; GCF_000001635.26), and *Homo sapiens* (GRCh38.p13; GCA_000001405.39), which were downloaded from NCBI.

For each reference organism, coding exons of full-length transcript were extracted and translated to peptides using the GeMoMa module Extractor. These peptides were searched in the GSD genome using mmseqs2 [72] (v5877873cbcd50a6d954607fc2df1210f8c2c3a4b). Based on the results of mmseqs2 and Extractor, transcripts were predicted for GSD from each reference organism independently. These 9 gene annotation sets were then combined into a final gene annotation using the GeMoMa module GAF.

Ribosomal RNA (rRNA) genes were predicted with Barrnap v0.9 (Barrnap, RRID:SCR_015995) [73] in the eukaryotic mode, HMMer v3.2.1 (Hmmer, RRID:SCR_005305) [74], and BEDTools v2.27.1 (BEDTools, RRID:SCR_006646) [69].

## Availability of Supporting Data and Materials

The complete genome build is available at NCBI (GenBank accession No. GCA_008641055.2). DNA Methylation data GEO accession is GSE136348. PAFScaff (PAFScaff, RRID:SCR_017976) is GPLv3 licensed and registered at bio.tools (biotools: PAFScaff_Pairwise_mApping_Format_reference-based_scaffold_anchoring_and_super-scaffolding.) [75]. All supporting data and materials are also available in the *GigaScience* GigaDB database [76].

## Additional Files

**Supplementary File 1**: Read depth analysis of *Amy2B* region

**Supplementary File 2**: Bionano *AMY2B* methods

**Supplementary File 3**: 10X chromium workflow details

**Supplementary Figure 1**: Schematic overview of project workflow. German Shepherd Dog (“Nala” or Jonkahra Nala) DNA was derived from blood of a single female . Her dam, Jonkahra Lets Elope, was from Australian breeding and the sire, CH Arkon Vom Altenberger Land was imported from Germany. Sequences were generated on the Pacific Biosciences Sequel instrument (V2 chemistry) and Oxford Nanopore PromethION instrument (guppy basecaller Version 3.0.6 + 9999d81) to ∼30× genome coverage, each, based on a genome size estimate of 2.4 Gb (this estimate is used for all coverage estimates). All long-read sequences were assembled with the Canu v1.8 algorithm then error corrected twice using the Arrow genomic consensus polishing module. The assembly was scaffolded with Chromium 10X linked reads (∼41× coverage excluding the barcode) using Long Ranger v2.1.6 using DNA from the same animal. Polishing of the assembly for residual indels was done by aligning the Illumina data with Minimap2 and the Racon algorithm. Single-molecule Bionano data (∼57× effective coverage) were then used to super-scaffold the sequence assembly using DNA extracted from the same canid. For this, single-molecule optical maps were first *de novo* assembled into consensus maps, which were than aligned to the sequence assembly *in silico* digested with the same labelling enzyme for hybrid scaffolding, using Bionano Solve (v3.2.2_0 802 2018) with RefAligner (7782.7865rel). This assembly was further scaffolded to chromosome length by the DNA Zoo following the methodology described here: www.dnazoo.org/methods. Briefly, an *in situ* Hi-C library was prepared from a blood sample of a purebred GSD male named Tydus and sequenced to 29× coverage. The Hi-C data were processed using Juicer and used as input into the 3D-DNA pipeline to produce a candidate chromosome-length genome assembly. We performed additional finishing on the scaffolds using Juicebox Assembly Tools. The assembly was then long-read gap filled with the PBJelly algorithm, and the additional data error corrected using Arrow. The Chromium data were mapped onto the assembly with the Long Ranger v2.1.6 program, and the final assembly was then polished using the Pilon algorithm. Of the 2.4-Gb assembled genome (German_Shepherd_breed-1.0), the total assembly N50 contig and scaffold lengths are 23.1 and 64.3 Mb, respectively. The genome was annotated using the homology-based gene prediction program GeMoMa (version 1.6.2beta) and 9 reference organisms. The assembled contigs were then aligned to CanFam3.1 for chromosome assignments.

**Supplementary Figure 2**: BUSCO improvements in assembly quality at each analysis step. **a**. BUSCO ratings for different stages of Nala assembly, compared to CanFam 3.1. See Supplementary Table 1 for descriptions of assembly stages. C: complete; S: single copy; D: duplicated; F: fragmented; M: missing; n: No. BUSCO genes. **b**. Missing BUSCO genes (%) vs scaffold NG50 (2.41-Gb genome size). Purple: original assembly; black: scaffolding/polishing steps; blue: final assembly; red: CanFam 3.1. Dashed red lines mark CanFam 3.1 statistics.

**Supplementary Figure 3**: KAT *k*-mer analysis of Nala assembly. 10X read *k*-mer frequency distributions for *k*-mers with different assembly copy numbers derived from **(A)** Read 1 (16 bp barcodes trimmed) and **(B)** Read 2 (barcodes not trimmed).

**Supplementary Figure 4**: Bionano consensus maps aligned to GSD contig NALACHR6.01. Overlay on the GSD contig (green bar) are 4 GeMoMa-predicted AMY2B transcripts, labelled R0–R3. Below and above the pair of Bionano consensus map alleles (blue bars) are single molecules (orange lines) supporting the genome map assembly. Dark blue and yellow vertical lines on the sequence contig and consensus map indicate matching and non-matching DLE1 enzymatic labels, respectively. DLE1 enzymatic labels on single molecules are shown as dark blue or light orange dots for matching and non-matching labels, respectively. Both genome map alleles harbour a ∼11-kb “insertion” upstream of the AMY2B repeat (highlighted in teal) and a ∼54.6-kb tandem duplication marked by DLE1 labels at positions 47,341,704 and 47,396,280 of the GSD NALACHR6.01 contig.

**Supplementary Figure 5**: Bionano consensus maps aligned to CanFam3 Chr6. Relative to CanFam3, the homozygous Bionano genome map alleles, Map Nos. 1111 and 1112, both contain an ∼100-kb insertion flanked by DLE1 labels at 46,954,644 and 46,999,962 on chromosome 6.

**Supplementary Figure 6**: Hip X-ray of the German Shepherd Dog Nala. Her combined hip score of 3 (1 on left-hand side and 2 on right-hand side) when the X-ray was taken at 5 years of age: each hip was measured on a 0–53 scale, with a total of 106 being crippling. This score is well below the current Australian average of 9 for GSDs.

**Supplementary Figure 7**: DNA methylation profiling of German Shepherd Dog Nala's whole blood. (**A**) Percentage of CpG dinucleotides with different levels of methylation. High, 80–100%; medium, 20–80%; low, >0–20%; no, 0. (**B**) Segmentation of hypomethylated regions into CpG-rich unmethylated regions (UMRs) and CpG-poor low-methylated regions (LMRs). The number of CpGs (log_2_) per region relative to its median methylation is shown. (**C**) Average DNA methylation profiles of UMRs and LMRs. (D) IGV browser track depicting mC profile and putative regulatory elements.

**Supplementary Table 1**: Summary assembly scaffold and BUSCO statistics for different Nala assembly stages, CanFam 3.1, and compiled best ratings.

**Supplementary Table 2**: BUSCO gene rating for different Nala assembly stages, CanFam 3.1, and compiled best ratings.

**Supplementary Table 3**: GSD SNVs and small indels summary by chromosome.

**Supplementary Table 4**: GSD copy number and structural variants (>100 bp) summary by chromosome.

giaa027_GIGA-D-19-00364_Original_Submission

giaa027_GIGA-D-19-00364_Revision_1

giaa027_GIGA-D-19-00364_Revision_2

giaa027_Response_to_Reviewer_Comments_Original_Submission

giaa027_Response_to_Reviewer_Comments_Revision_1

giaa027_Reviewer_1_Report_Original_Submission Jocelyn Plassais -- 12/6/2019 Reviewed

giaa027_Reviewer_2_Report_Original_Submission Klaus-Peter Koepfli, PhD -- 12/17/2019 Reviewed

giaa027_Reviewer_2_Report_Revision_1 Klaus-Peter Koepfli, PhD -- 2/1/2020 Reviewed

giaa027_Supplemental_Files

## Abbreviations

BLAST: Basic Local Alignment Search Tool; BMG: Bionano Genomics; bp: base pairs; BUSCO: Benchmarking Universal Single-Copy Orthologs; CHD: canine hip dysplasia; CNV: copy number variant; Gb: gigabase pairs; GPU: graphics processing unit; GSD: German Shepherd Dog; HMM: hidden Markov model; HMW: high molecular weight; kb: kilobase pairs; LMR: low-methylated region; Mb: megabase pairs; mRNA: messenger RNA; NCBI: National Center for Biotechnology Information; ONT: Oxford Nanopore Technologies; OR: olfactory receptor; ORF: open reading frame; PacBio: Pacific Biosciences; qPCR: quantitative polymerase chain reaction; rRNA: ribosomal RNA; SMRT: single-molecule real time; SNP: single-nucleotide polymorphism; SNV: single-nucleotide variant; SV: structural variant; UMR: unmethylated region.

## Ethics Approval and Consent to Participate

All experimentation was performed under the approval of the University of New South Wales Ethics Committee (ACEC ID: 18/18B).

## Competing Interests

The authors declare that they have no competing interests.

## Funding

This work was supported by the Australian Health Foundation award and by the Hip2Fit Crowdfunding initiative to J.W.O.B. and R.Z. Matching funds were provided by the University of New South Wales/School of Biotechnology and Biomolecular Sciences Genomics Initiative. V.M.H. funded the Bionano data collection. Hi-C scaffolding was performed and funded by the DNA Zoo Consortium. M.A.F. is funded by NHMRC APP5121190. E.L.A. was supported by an NSF Physics Frontiers Center Award (PHY1427654), the Welch Foundation (Q-1866), a USDA Agriculture and Food Research Initiative Grant (2017–05741), an NIH 4D Nucleome Grant (U01HL130010), and an NIH Encyclopedia of DNA Elements Mapping Center Award (UM1HG009375). The Ramaciotti Centre for Genomics acknowledges infrastructure funding from the Australian Research Council (LE150100031), the Australian Government NCRIS scheme administered by Bioplatforms Australia, and the New South Wales Government Research Attraction and Acceleration Program .

## Authors' Contributions

J.W.O.B. coordinated the project. M.A.F., B.D.R., T.P.L.S., and J.W.O.B. designed the study. J.W.O.B funded the project. R.A.Z. provided the GSD samples. R.L. and D.T. performed genomic DNA extractions. K.B. and M.A.S. performed the ONT sequencing, and R.L., the Bionano optical mapping. B.D.R. performed the initial assembly and polishing, A.E.R. performed the chromium scaffolding, and E.K.F.C and V.M.H. performed the Bionano super-scaffolding. O.D., A.O., and Z.C. performed the Hi-C experiment, and O.D. and E.L.A. conducted the Hi-C analyses. K.S. and O.B. conducted the DNA methylation analyses. M.A.F. and R.E performed all analyses of genome completeness. R.E. performed the final polishing, final assembly cleanup, and KAT analysis. J.K. performed the genome annotation. R.E. performed the rRNA annotation. R.E., E.K.F.C., and B.D.R. performed the *AMY2B* analyses. M.A.F., B.D.R., O.D., R.E., A.E.M., E.K.F.C., O.B., and J.W.O.B. wrote the manuscript. All authors edited and approved the final manuscript.
