# Supplementary material for: Canfam_GSD: De novo chromosome-length genome assembly of the German Shepherd Dog (Canis lupus familiaris) using a combination of long reads, optical mapping, and Hi-C
Source: Gigascience. 2020 Apr 1;9(4):giaa027. doi: 10.1093/gigascience/giaa027 (PMC7111595; doi:10.1093/gigascience/giaa027)
Supplement: giaa027_GIGA-D-19-00364_Revision_1 [file giaa027_giga-d-19-00364_revision_1.pdf]

GigaScience

Canfam_GSD: De novo chromosome-length genome assembly of the German Shepherd Dog (Canis lupus familiaris) using a combination of long reads, optical mapping and Hi-C

--Manuscript Draft--

Manuscript Number:	GIGA-D-19-00364R1
Full Title:	Canfam_GSD: De novo chromosome-length genome assembly of the German Shepherd Dog (Canis lupus familiaris) using a combination of long reads, optical mapping and Hi-C
Article Type:	Research
Funding Information:	
Abstract:	The German Shepherd Dog (GSD) is one of the most common breeds on earth and has been bred for its utility and intelligence. It is often first choice for police and military work, as well as protection, disability assistance and search-and-rescue. Yet, GSD's are well known to be afflicted with a range of genetic diseases that can interfere with their training. Such diseases are of particular concern when they occur later in life, and fully trained animals are not able to continue their duties.<br>Here, we provide the draft genome sequence of a healthy German Shepherd female as a reference for future disease and evolutionary studies We generated this improved canid reference genome utilising a combination of Pacific Bioscience, Oxford Nanopore,, 10X Genomics, Bionano, and Hi-C technologies. The GSD assembly is approximately 80 times as contiguous as the current canid reference genome (20.9 Mb vs 0.267 Mb contig N50), containing far fewer gaps (306 vs 23,876) and fewer scaffolds (429 vs 3,310) than the current canid reference genome CanFam v3.1. Two chromosomes (4 and 35) are assembled into single scaffolds with no gaps. Benchmarking Universal Single-Copy Orthologs analyses of the genome assembly results show 93.0% of the conserved single-copy genes are complete in the GSD assembly compared to 92.2% for CanFam v3.1. Homology-based gene annotation increases this value to about 99%. Detailed examination of the evolutionary important pancreatic amylase region reveals there are most likely seven copies of the gene indicative of a duplication of four ancestral copies and the disruption of one copy.<br>GSD genome assembly and annotation were produced with major improvement in completeness, continuity and quality over the existing canid reference. This resource will enable further research related to canine diseases, the evolutionary relationships of canids, and other aspects of canid biology.
Corresponding Author:	J. William O. Ballard, Ph.D.<br>University of New South Wales<br>Sydney, NSW AUSTRALIA
Corresponding Author Secondary Information:	
Corresponding Author's Institution:	University of New South Wales
Corresponding Author's Secondary Institution:	
First Author:	Matt A Field, PhD
First Author Secondary Information:	
Order of Authors:	Matt A Field, PhD
	Benjamin D. Rosen
	Olga Dudchenko
	Eva K.F. Chan
	Andre E. Minoche
	Richard J. Edwards

Powered by Editorial Manager® and ProduXion Manager® from Aries Systems Corporation

	Kirston Barton
	Ruth J. Lyons
	Daniel Enosi Tuipulotu
	Vanessa M. Hayes
	Arina Omer
	Zane Colaric
	Jens Keilwagen
	Ksenia Skvortsova
	Ozren Bogdanovic
	Martin A Smith
	Erez Lieberman Aiden
	Timothy P.L. Smith
	Robert A. Zammit
	J. William O. Ballard
**Order of Authors Secondary Information:**	

Response to Reviewers:

Dear Editor;

Thank you for giving us the opportunity to respond to the Reviewers comments. Our replies are in bold below and appropriately in text. We now hope the manuscript is acceptable for publication in GigaScience.

We found the reviewers comments highly constructive and so they are acknowledged. In the Acknowledgements we also recognise comments from James Ferguson.

Due to his scientific contribution to the Promethion sequencing Martin A. Smith is now included as a co-author. Additionally, author Richard J. Edwards position in the manuscript was adjusted to acknowledge his significant contribution during the review process. All affected authors have been notified and agreed to this change.

For clarity we have re-drawn Supplementary Figure 1.

Also, added key words to the manuscript. The words we have included are:
Hi-C, long read sequencing, optical mapping, de novo genome assembly, canine hip dysplasia, DNA Zoo.

Yours truly,
J. William O. Ballard
University of New South Wales
Sydney
Australia, 2052

GIGA-D-19-00364
De novo genome assembly of German Shepherd Dog (Canis lupus familiaris)
Matt A Field, PhD; Benjamin D. Rosen; Olga Dudchenko; Eva K.F. Chan; Andre E. Minoche; Kirston Barton; Ruth J. Lyons; Daniel Enosi Tuipulotu; Richard J. Edwards; Vanessa M. Hayes; Arina Omer; Zane Colaric; Jens Keilwagen; Ksenia Skvortsova; Ozren Bogdanovic; Erez Lieberman Aiden; Timothy P.L. Smith; Robert A. Zammit; J. William O. Ballard
GigaScience

Dear Professor Ballard,

Your manuscript "De novo genome assembly of German Shepherd Dog (Canis lupus

familiaris)" (GIGA-D-19-00364) has been assessed by our reviewers. Although it is of interest, we are unable to consider it for publication in its current form. The reviewers have raised a number of points which we believe would improve the manuscript and may allow a revised version to be published in GigaScience.

Their reports, together with any other comments, are below. Please also take a moment to check our website at https://www.editorialmanager.com/giga/ for any additional comments that were saved as attachments.

In addition, please register any new software application in the bio.tools and SciCrunch.org databases to receive RRID (Research Resource Identification Initiative ID) and biotoolsID identifiers, and include these in your manuscript. This will facilitate tracking, reproducibility and re-use of your tool.

If you are able to fully address these points, we would encourage you to submit a revised manuscript to GigaScience. Once you have made the necessary corrections, please submit online at:

https://www.editorialmanager.com/giga/

If you have forgotten your username or password please use the "Send Login Details" link to get your login information. For security reasons, your password will be reset.

Please include a point-by-point within the 'Response to Reviewers' box in the submission system. Please ensure you describe additional experiments that were carried out and include a detailed rebuttal of any criticisms or requested revisions that you disagreed with. Please also ensure that your revised manuscript conforms to the journal style, which can be found in the Instructions for Authors on the journal homepage. If the data and code has been modified in the revision process please be sure to update the public versions of this too.

The due date for submitting the revised version of your article is 17 Mar 2020.

I look forward to receiving your revised manuscript soon.

Best wishes,

Hongling Zhou
GigaScience
www.gigasciencejournal.com

Reviewer reports:

Reviewer #1: The manuscript" De novo genome assembly of German Shepherd Dog (Canis lupus familiaris)" presents a new genome assembly for the dog model community. The authors chose to sequence one of the most famous breeds, the German Shepherd, with recent technologies including long read sequencing using PacBio SMRT, and ONT PromethION sequencing. Their goal is to improve the genome reference used by the dog community. They also explored the methylome and produced HI-C library using a second dog. They provide detailed methodology and used the example of the AMY2B locus to illustrate how this new reference sequence can be used to capture missing data, particularly around structural variations such as copy number variations. As a resource paper, this work has great potential and will be heavily citable. Regarding the quality of the work, I have a few comments/questions:

1) Taking a look on NCBI, I noticed two other full high-quality genome assemblies available for Canis lupus familiaris, since CanFam3.1 was initially made available in 2011. Did the authors try to contact the two other groups to compare data, as you did with canfam3.1? This manuscript gives rise to a major question in the dog community concerning the nomenclature used for new reference genomes, i.e. since many new references are being produced, I recommend that the authors name their assembly in a way that is easily recognizable and discernable. Using CanFam 4 is far from ideal since all previous dog genome references (canfam1, 2, 3) were produced using the same Boxer, Tasha. An alternative would be to use a name like CanfamGSD.1, for

example? This sets the stage for other additional de novo assemblies. Each can retain the CanFam designation, but an abbreviation to indicate which dog/breed it is. This issue must be dealt with.

REPLY: We did approach other groups but were told unequivocally that they were not interested. We have added Canfam_GSD to the title and Findings section of the abstract.

I also wondering how these data will be shared with the scientific community. Do you also plan to submit your data to established genome browsers commonly used by the scientific community (i.e: Ensembl, UCSC...?). Presumably yes in order that it will be widely used. The paper should contain this information.

REPLY: The genome is present at http://www.dnazoo.org/assemblies/Canis_lupus_familiaris_German_Shepherd and is also in a locally hosted Apollo browser. Like our Dingo genome assembly it will be transferred to Ensembl.

2) I noticed that there is no data for the mitochondrial DNA. Is it a choice from the authors or a technical limitation? The actual Boxer reference CanFam3.1 has mitochondrial sequence as does the Great Dane sequence submitted to NCBI this year. Since the authors proposed their new genome as a future dog genome reference, can they explain in one or two sentences why they did not work on the mitochondrial DNA.

REPLY: In addition to the nuclear genome, the mitochondrial genome was assembled and has been uploaded to GigaScience for immediate download. It also has been deposited to NCBI and is being processed. Added to the manuscript L155
The mitochondrial genome assembly was also assembled and has been uploaded to GigaScience for immediate download. It has been deposited to NCBI and is being processed.

3) Concerning the annotation: did the authors improve the annotation of keratin clusters (CFA9, for example) or the olfactory receptor clusters that are typically poorly annotated? If yes, please discuss. These are some of the biggest flaws in the previous genome assembly and it is imperative that this be highlighted in this paper. Alternatively, if large paralog gene families continue to present a problem, it is worth mentioning. Also, in order to provide the most completely annotated genome reference, I recommend the authors include all non-coding RNA (miRNAs, lncRNAs) recently published data (See Wucher et al, Nuc Acid.Res 2017, Megquier K et al, Genes 2019). It could be also interesting to compare your data with the observations published last year (Holden et al, Sci Rep. 2018) which identified missing gene sequences associated with diseases using unmapped sequences.

REPLY regarding OF and keratin: We have added a new section on L269 in the Methods after “Pancreatic amylase (AMY2B) analysis”.

"Olfactory receptor and Keratin cluster analysis

Correct annotation of the olfactory and keratin clusters in canines has been problematic but it is important for research on canid health and evolution [21, 30, 31]. Dogs are macrosmatic animals that rely highly on their sense of smell. Yet, the molecular basis of such prominent chemosensory capacities remains largely unknown. The ability to detect and discriminate the multitude of odors in vertebrates is mediated by a superfamily of G-protein-coupled olfactory receptor (OR) proteins [32]. Based on the description in the reference annotations, we filtered all mRNAs of the references that contain in their description the regular expression “olfactory receptor”. We then extracted the number of mRNAs and genes per reference organism. Subsequently, we used the IDs to filter the GSD annotation and counted the number of predicted mRNAs and genes. This procedure identifies 1250 mRNAs and 933 genes in the GSD and 849 mRNAs and 804 genes in the boxer. Quignon et al. [30] identified five amino acid patterns characteristic of ORs in the canine genome and retrieved 1,094 dog genes (872 genes and 222 pseudogenes).

Powered by Editorial Manager® and ProduXion Manager® from Aries Systems Corporation

	Keratins are filament proteins of the epithelial cytoskeleton and are essential for normal skin homeostasis. Over time the genes encoding keratins have undergone multiple rounds of duplication with high similarity between different keratin paralogs [31]. Analogously to the olfactory receptor study, we filtered all mRNAs of the references that contain in their description the keyword "keratin \d". This procedure identifies 118 mRNAs and 83 genes in the GSD and 73 mRNAs and 55 genes in the boxer. Balmer et. al. [31] investigated the National Center for Biotechnology Information (NCBI) (dog annotation release 103) gene predictions for the canine gene clusters to RNA-seq data that were generated from adult skin of five dogs and adult hair follicle tissue of one dog and annotated 61 putatively functional keratin genes in the dog."<br><br>Also, to link with this inclusion we have added a new paragraph in the Discussion L323.<br><br>"The assembly is expected to enable the selection of GSDs for particular duties including police work where their sensitive nose is frequently used to discriminate odors. Robin et al. [30] analyzed the nucleotide sequences of 109 OR genes (102 genes and seven pseudogenes) in six different breeds including GSDs. Most generally they show that OR genes are highly polymorphic, with a mean of one SNP per 577 nucleotides. However, the degree of polymorphism observed is highly variable, with some OR genes having few if any SNPs and others being highly polymorphic (1 SNP/122 nt). Yang et al. [33] conducted a preliminary study of 22 SNPs from the exonic regions of 12 OR genes in GSDs and found a significant correlation between SNP genotypes of DR genes and olfactory abilities of dogs."<br><br>REPLY regarding rRNA: We have not included RNA data in this study and suggest that annotating all non-coding RNA is beyond the scope of the study. We have now, however, annotated the rRNA genes and added a section to the methods (L683) and a file with annotations is now available (rRNA_predictions.gff)<br><br>"rRNA genes were predicted with Barrnap v0.9 (https://github.com/tseemann/barrnap) in the eukaryotic mode, implementing Perl v5.28.0, HMMer v3.2.1 [66] and BEDTools v2.27.1 (https://github.com/arq5x/bedtools2). "<br><br>REPLY regarding Holden et al. contigs: We have mapped all of the contigs from the Holden et al. study onto both assemblies and demonstrated a marked improvement in coverage with GSD compared to CanFam3.1.<br>Analysis of novel contigs assembled by Holden et al. from canine reads that failed to map to CanFam 3.1 further supports the greater completeness of GSD. For all three dog breeds assembled by Holden et al., a greater proportion of the combined contigs map on to GSD than CanFam3.1 (94.3% vs 85.4% Border Collie; 77.0% vs 33.9% Bearded Collie; 76.8% vs 42.1% Entlebucher Sennenhund).<br>Note: This work was not included in the final manuscript.<br><br><br>4) Concerning the AMY2B story, the authors describe how Bionano technology analyzed the relevant CNV but did not discuss their results in an evolutionary context, such as what are the consequences of higher copy number. What hypothesis can be made concerning the complete loss of AMY2B locus observed in CanFam3.1 in comparison with the GSD genome? Is it related to the breed creation (Boxer vs GSD), or just a polymorphic CNV in modern breeds without any particular selection in current times? For example, it could be very interesting to see if, using your genome reference, you can use the GSD genomes already published and available on SRA and other related breeds to: 1) confirm your observations on this locus, and 2) explore breeds that you cite in your discussion (L490-496), checking how many copies each has on this locus. This will provide insight regarding about the evolutionary position of the GSD as regards the two cited papers L493-494 (Bigi et al 2015, Parker et al, 2017).<br><br>REPLY: The AMY2B story is an interesting and complex one. There is an expansion in all domestic breeds (including Boxer) relative to the wolf. We are currently de novo sequencing Desert and Alpine Australian dingoes to gain greater insight into this question. We suggest additional analyses is beyond the scope of the current manuscript.

Powered by Editorial Manager® and ProduXion Manager® from Aries Systems Corporation

5) This comment follows my first comment and is maybe beyond this paper: I noticed the first author released the Dingo genome (Canis lupus dingo), submitted on NCBI in June 2018 (Bioproject: PRJNA477859) and already available on Ensembl. I did not find an associated paper to this work, so I am wondering if authors could make a comparison between both Dingo and GSD in this paper, highlighting potential genomic differences which would support the last sentence of your abstract: "This resource will enable further research related to canine diseases, the evolutionary relationships of canids, and other aspects of canid biology".

REPLY: You are correct the Canis lupus dingo was submitted on NCBI in June 2018. Currently, we are in the process of improving the assembly of the Desert Dingo (v2) so that more robust comparisons can be made with the Wolf and domestic dogs (including the AMY2B expansion story, which is still unfolding). As indicated above we are also de novo sequencing the Alpine Dingo and and aim to also include a new de novo Basenji assembly.

I noticed some typos:

L98: "best-known".
REPLY. Corrected

L186: Pancreatic amylase (AMY2B) analysis.
REPLY. Corrected

L221-223: For a better understanding, I recommend your write this sentence like this: "Alignment results confirmed the presence of seven repeat units, showing a perfect alignment to the seven-copy sequence construct (Figure 2A), but a "deletion" of one repeat unit relative to the eight-copy construct (Figure 2B)."
REPLY. Corrected OK.

L290-292: Can you re-write this sentence please - I do not understand what is largely methylated and largely unmethylated?
REPLY: Rewritten as “In concordance with other adult vertebrates [40, 41], the GSD genome displays a typical bimodal DNA methylation pattern with over 60% of CpG dinucleotides being methylated at levels higher than 80% (hypermethylated) and 12% of CpG dinucleotides being methylated at 20% or lower (hypomethylated).”

L297-299: you should specify "in other models" because these references did not work on dogs.
REPLY: OK. added.

L315: Please correct " Saphyr instrument"
REPLY. Corrected

L465: remove uppercase for "annotation".
REPLY. Corrected

Supplementary Table 1: typo in title: "statistics" when you open the file.
REPLY. Corrected

Reviewer #2: General comments
The authors report on the de novo genome assembly and annotation of a female German shepherd, a popular and well-known breed of domestic dog. The chromosome-length genome was generated using a combination of sequences obtained from PacBio and ONT long reads,10X Genomics Chromium libraries, Bionano Genomics optical maps and Hi-C libraries. The assembly was produced using an iterative workflow in which the long-read data were first assembled into contigs and these were then successively scaffolded using the 10X Genomics Chromium data, optical maps and Hi-C data. Each stage of the assembly was evaluated in terms of continuity and completeness as judged by BUSCO scores. The hybrid assembly was subjected to three rounds of rigorous polishing to produce an accurate and highly continuous assembly. In fact, the German shepherd dog assembly is 80X more contiguous than the standard domestic dog reference genome, CanFam version 3.1 (based on the boxer breed). As such, the newly

Powered by Editorial Manager® and ProduXion Manager® from Aries Systems Corporation

reported assembly achieves an important benchmark in terms of genome assembly quality.

Specific comments
The manuscript is generally well written and the methods of the genome sequencing and assembly are thoroughly presented in detail in both the main text and the Supplementary Materials. The newly reported assembly represents one of the most contiguous genome assemblies yet generated for a mammalian species outside of humans and will no doubt provide an important resource for countless studies in addition to the CanFam version 3.1 assembly which has been the primary assembly for many years. The in-depth annotation and analysis of the AMY2B copy number is especially thorough and well done. The paper will no doubt stimulate great interest by researchers in mammalian genomics as well as the general public. I recommend the manuscript be accepted for publication once the authors have addressed specific comments that I think would improve the clarity and impact of the manuscript, as described below.

1. There are no page numbers or line numbers, which makes it unnecessarily difficult to provide comments on the manuscript. Therefore, I will reference my comments according to the page number and manuscript section based on the pdf version of the manuscript. Please add page numbers and line numbers in future versions of the manuscript.
REPLY: OK. added.

2. In the provided manuscript, the Methods section was placed before the Discussion section. As this has been submitted as a Research article, the Methods section should be moved to after the Discussion section.
REPLY: Moved (but did not include track changes in this cut-and-paste).

3. Contig assembly, scaffolding and polishing were evaluated using N50 and BUSCO scores. However, I recommend the authors also assess the German Shepherd dog assembly using the K-mer Analysis Toolkit (KAT; Mapleson et al. 2017 Bioinformatics 33: 574-576 and https://github.com/TGAC/KAT). KAT uses k-mer frequencies to profile errors, GC-bias, and other metrics along with providing quality control checking of assemblies at different stages. This would involve using the PacBio and ONT long reads as well as the de-barcoded raw reads from the 10X Genomics Chromium sequencing that were generated.

REPLY: We thank the reviewer for this suggestion and applied KAT to our assembly. The per-base error rates were too high for PacBio or ONT reads to yield useful kmer analysis, but the 10x reads provided useful additional QC. KAT does not provide an explicit way to compare different stages of the assembly, so instead we have used it for QC of our final assembly. We have added the following paragraph (L179) and included a new Supplementary Figure (Supplementary Figure 3). This addition necessitated renumbering of figures.

"Additional k-mer analysis of the final assembly was performed using KAT v2.4.2 [26]. KAT comp was used to compare k-mer frequencies from the 10x reads (16 bp barcode trimmed from read 1) with their copy number in the assembly. This comparison revealed no sign of missing data nor large duplications, including retention of haplotigs (Supplementary Figure 3)."

4. Title: I suggest revising the title of the manuscript so that it is less generic and more descriptive of the methods/technologies used:
"De novo chromosome-length genome assembly of the German Shepherd Dog (Canis lupus familiaris) using a combination of long reads, optical mapping and Hi-C".

REPLY: We have modified the title as you suggested and added “Canfam_GSD” to the start of the title at the suggestion of reviewer 1. The new title reads “Canfam_GSD: De novo chromosome-length genome assembly of the German Shepherd Dog (Canis lupus familiaris) using a combination of long reads, optical mapping and Hi-C”.

5. Page 8, Results, Workflow: "Contigs were assembled using SMRT and ONT sequencing and then polished to minimize error propagation." Although the authors

Powered by Editorial Manager® and ProduXion Manager® from Aries Systems Corporation

refer to Supplementary Figure 1 in this paragraph, for this sentence, the authors should reference the program(s) used for polishing and also the section of the Methods where this is detailed.

REPLY: OK, programs referenced and section of the Methods where this is detailed supplied.

6. Page 9, Results, Assembly stats / completeness: "…against Laurasiatheria_ob9 (n=6,253)…" This should be revised as "…against the Laurasiatheria_ob9 data set (n=6,253)…"
REPLY: OK. Added.

7. Page 10, Table 1: In column three (CanFam3.1) and the row, "BUSCO complete (genome)", the value 91.10.8% single copy doesn't make sense. I assume the authors mean 91.1%. Please correct.
REPLY: OK. Thanks.

8. Page 10, Results: "Based on the existing CanFam3.1 annotation and the GSD annotation provided by GeMoMa…" Please provide a reference for the GeMoMa software.
REPLY: OK add GeMoMa ref.

9. Page 11, Results: "Pacreatic amylase (AMY2B) analysis" should be corrected as "Pancreatic amylase (AMY2B) analysis".
REPLY: OK. Corrected.

10. Page 11, Results, Pancreatic amylase (AMY2B) analysis: "The longest read in the region covered three plus copies…" This description of the number of copies is vague. The following revision is suggested: "The longest read in the region covered between three to [insert maximum number observed] copies…"
REPLY: Replaced with The long sequencing reads in the region covered no more than three complete copies…

11. Page 11, Results, Pancreatic amylase (AMY2B) analysis: "Further examination of this region was attempted using both the Bionano genome map…" Suggested revision: "Further examination of this region was attempted using both the Bionano optical map…"
REPLY: OK

12. Page 13, Methods, Sampling: Nala the German Shepherd Dog: "Nala had a combined hip score of 3 (1 on LHS and 2 on RHS) when the x-ray was taken at 5 years of age…" Not all readers may understand the LHS and RHS notation. Suggested revision: "Nala had a combined hip score of 3 (1 on the left hand side and 2 on the right hand side) when the x-ray was taken at 5 years of age…"
REPLY: OK.

13. Page 14, Methods, Pacific Bioscience Single Molecule Real-Time (SMRT) sequencing: "…and molecular integrity was assessed using pulse-field gel electrophoresis." In the interests of repeatability, the authors should provide more details about the PFGE experiment(s) including the instrument used (if commercial instrument), gel type and concentration, the voltage, run time, and the amount of DNA run and DNA standards used.
REPLY: Added L382 "DNA integrity was assessed by the Sage Science Pippin Pulse. A 0.75% KBB gel was run on the 9hr 10-48kb (80v) program. DNA ladder used was the Invitrogen 1kb Extension DNA ladder (cat 10511-012). 150ng of DNA was loaded on the gel."

14. Page 14, Methods, Pacific Bioscience Single Molecule Real-Time (SMRT) sequencing:
"…and at the Arizona Genomic Institute, University of Arizona (four SMRT cells with a 11Gb of data: NOTE: short read lengths were due to DNA shearing of the DNA during shipping from Australia to Arizona)." Suggested revision: "…and at the Arizona Genomic Institute, University of Arizona (four SMRT cells with a total of 11Gb of data;

NOTE: short read lengths were due to DNA shearing of the DNA during shipping from Australia to Arizona)."
REPLY: OK. Thanks.

15. Page 15, Methods, 10X Genomics Chromium sequencing: The authors should reference Supplementary File 3 at the end of this paragraph, which provides a more detailed description of the methods used for the 10X Genomics Chromium sequencing. Also, Supplementary File 3 should be retitled as "10X Genomics Chromium sequencing: Detailed Methods."
REPLY: OK.

16. Page 15, Methods, 10X Genomics Chromium sequencing: "…was barcoded from high-molecular-weight DNA according to manufacturers recommended protocols." Suggested revision: "…was barcoded from high-molecular-weight DNA according to the manufacturer's recommended protocols." Also, the authors should provide a reference for these protocols (i.e., a manual document or website URL).
REPLY: Now reads (L423)
"Protocol used was the Chromium Genome Reagent Kits v2 User Guide, manual part number CG00043 Rev B available here: https://support.10xgenomics.com/genome-exome/library-prep/doc/user-guide-chromium-genome-reagent-kit-v2-chemistry."

17. Page 15, Methods, 10X Genomics Chromium sequencing: "QC was performed using LabChip GX and Qubit." Please provide manufacturer information and location for both of these instruments.
REPLY: Added on L426
"QC was performed using LabChip GX (PerkinElmer, MA, USA) and Qubit 2.0 Flurometer (Life Technologies, CA, USA) at the Kinghorn Centre for Clinical Genomics."

18. Page 15, Methods, Methylome: "…using MethylSeekR algorithm [27]." Suggested revision: "…using the MethylSeekR algorithm [27]."
REPLY: OK.

19. Page 16, Methods, Bionano optical mapping: "Multiple cycles were performed to reach average raw genome depth of coverage of 190X." Suggested revision: "Multiple cycles were performed to reach an average raw genome depth of coverage of 190X."
REPLY: OK.

20. Page 17, Methods, 10X Chromium linked-reads: "The arrow polished SMRT/ONT assembly was scaffolded…" Suggested revision: "The Arrow-polished SMRT/ONT assembly was scaffolded…"
REPLY: OK.

21. Page 17, Methods, 10X Chromium linked-reads: "The 10X data was aligned using the linked-read analysis software provided by 10X Genomics, Long Ranger, v2.1.6 (https://www.10xgenomics.com/), misaligned reads and reads not mapping to contig ends were removed, all possible connections between contigs were computed keeping best reciprocal connections." This sentence is overly complicated and doesn't make sense as written. I suggest breaking this into two sentences and revising as follows: "The 10X read data was aligned using the Long Ranger, v2.1.6 software (https://www.10xgenomics.com/). Misaligned reads and reads not mapping to contig ends were removed, and all possible connections between contigs were computed by keeping the best reciprocal connections."
REPLY: OK suggested correction implemented. Thanks, that's better now.

22. Page 18, Methods, Optical mapping for super-scaffolding using Bionano data: "Alignments indicating conflict between the sequence and optical maps, and hence suggestive of mis-assembly, were evaluated such that, conflicts supported by single-molecule optical maps (thus supporting optical map) would cause the sequence map to be "cut" at the conflict point, else the optical map was "cut"." This meaning and method described in the second part of this sentence is not clear (after "were evaluated such that"). Please revise to improve clarity.
REPLY: Replaced on L525 with "Alignments indicating conflict between the sequence and optical maps, and hence suggestive of mis-assembly, were resolved. Specifically,

	optical maps supported by at least ten single molecules at the conflict site were indicative of sequence mis-assembly, and so the sequence map would be "cut" (split) at the conflict point. In contrast, insufficient single molecule support for the optical map was indicative of optical map assembly error, and so the optical map would be "cut" at the conflict site.”<br><br>23. Page 19, Methods, Gap filling: "After scaffolding and correction, all raw reads were aligned to the assembly…" Which raw reads are the authors referring to here? The raw reads from the 10X Genomics Chromium sequencing? Please specify.<br>REPLY: "After scaffolding and correction, all raw SMRT and ONT reads were aligned to the assembly."<br><br>24. Page 20, Methods, Polishing Round 2: "…and correcting the single nucleotide polymorphism's (SNP) and indels using Pilon [43]." Suggested revision: "…and correcting the single nucleotide polymorphisms (SNPs) and indels using Pilon [43]."<br>REPLY: OK.<br><br>25. Page 20, Methods, Low-coverage filter: "Any scaffolds with median coverage less than 3 (e.g., less than 50% of the scaffold covered by at least three reads) were filtered as Low Coverage." To improve clarity, I suggest the following revision: "Any scaffolds with median coverage less than 3 (e.g., less than 50% of the scaffold covered by at least three reads) were filtered out as Low Coverage scaffolds."<br>REPLY: OK. Thanks.<br><br>26. Pages 20-21, Methods, Purge Haplotigs analysis - round 1 and round 2: "Subreads were re-mapped on to the remaining 837 scaffolds…" and "Subreads were re-mapped on to the remaining 558 scaffolds…" It's not clear where these remaining scaffolds originate from in the two paragraphs about purging of the haplotigs. Are these the scaffolds remaining after primary assembly using the PacBio+ONT+10X Genomics Chromium+Bionano optical map+Hi-C? Also, after the first round of purging, it seems only 279 scaffolds were processed, leaving 558 scaffolds to be processed in the second round. Is this correct? The authors need to better clarify these points so that they are more understandable for readers.<br>REPLY: Added/ clarified three sentences as described below. Also, we added a small section on our final contamination screening step.<br><br>[Low-coverage filter]<br>Of the 1,057 Pilon-polished scaffolds, 220 scaffolds were removed in the initial Low-coverage filter, leaving 837 scaffolds.<br><br>[Purge Haplotigs analysis - round 1]<br>This analysis resulted in a further 11 scaffolds filtered for low coverage and 268 filtered as haplotigs or assembly artefacts, leaving 558 scaffolds.<br><br>[Purge Haplotigs analysis - round 2]<br>Subreads were re-mapped on to the remaining 558 scaffolds resulting in a further 128 scaffolds filtered as haplotigs or assembly artefacts leaving 430 scaffolds.<br><br>[Final Scaffold Classification]<br>Twenty REPEAT scaffolds corresponding to a PacBio control sequence were removed from the assembly, leaving the final 409 nuclear scaffolds plus mitochondrion. Seventeen scaffolds had small regions masked or trimmed by the NCBI Contamination screen, corresponding to a 3.4kb chunk of Escherichia coli.<br><br>27. Page 21, Methods, Purge Haplotigs analysis - round 2: "A single remaining Scaffold marked as JUNK…" Scaffold in this sentence does not need to be capitalized.<br>REPLY: OK.<br><br>28. Page 21, Methods, CanFam3.1 Chromosome Mapping: The PAFScaff v0.2.0 program - please provide a reference or URL for this software.<br>REPLY: Reference to https://github.com/slimsuite/pafscaff added. The tool has also been registed at SciCrunch.org and bio.tools, as requested by the editor.

	29. Pages 22-23, Methods, Gene prediction including Annotation of repetitive elements: Please provide NCBI accession numbers for the assembly/annotation of the nine species used for the homology-based gene prediction analyses.<br>REPLY: Now reads on L659:<br>The nine species used for the homology-based gene prediction analyses were Canis lupus familiaris (CanFam3.1; GCF_000002285.3), Vulpes vulpes (VulVul2.2; GCF_003160815.1), Felis catus (Felis_catus_9.0; GCF_000181335.3), Sus scrof (Sscrofa11.1; GCF_000003025.6), Bos taurus (ARS-UCD1.2; GCF_002263795.1), Ailuropoda melanoleuca (ASM200744v1; GCF_000004335.2), Ursus maritimus (UrsMar_1.0; GCA_000687225.1), Mus musculus (GRCm38.p6; GCF_000001635.26), and Homo sapiens (GRCh38.p13; GCA_000001405.39), which were downloaded from NCBI.<br><br>30. Page 23, Discussion: "…commonly registered KC breeds…" Please spell out "KC" when used for the first time. I assume the authors mean Kennel Club here.<br>REPLY: OK.<br><br>31. Page 23, Discussion: I suggest the authors expand on the discussion in the first paragraph of the Discussion section by describing in a few sentences how the German shepherd dog genome assembly can be applied "for advancing knowledge of breed specific diseases." As currently written, the paragraph only provides a description of diseases that German shepherds are pre-disposed to. Surely, the genetic etiology of some of these conditions/diseases has been previously researched. The authors should reference some of these studies and then detail how the high-quality reference genome they have generated will advance such research.<br>REPLY: Added new paragraph L310<br>"The high quality genome assembly will advance knowledge of breed specific diseases such as CHD and extend to issues related to canine personality. The severity of CHD depends on both genetic and environmental factors. In GSDs, the heritability (h2) estimates have varied from 0.1 to 0.6 [53]. To date, different study populations and methods affect the results substantially, as the reported quantitative trait locus (QTL) association and candidate genes are inconsistent between studies [54-56]. While boxers are prone to CHD the hip scores of Tasha, used for CanFam, are unknown. Further, GSD specific SNPs as well as significant CNVs and SVs are difficult to detect. In a cohort containing over 10 000 behaviorally tested GSD and Rottweiler dogs Saetre et al. [57] examined how traits are transmitted between generations. In both breeds, the pattern of co-inheritance was found to be similar for a broad personality trait previously named shyness–boldness with heritability estimated to be 0.25 in the two breeds. Currently, the underling genes involved in these behaviours are not known."<br><br>--<br>Please also take a moment to check our website at https://www.editorialmanager.com/giga/l.asp?i=61160&l=IAAB5AEW for any additional comments that were saved as attachments. Please note that as GigaScience has a policy of open peer review, you will be able to see the names of the reviewers.<br><br>____<br>In compliance with data protection regulations, you may request that we remove your personal registration details at any time. (Use the following URL: https://www.editorialmanager.com/giga/login.asp?a=r). Please contact the publication office if you have any questions.
**Additional Information:**	
**Question**	**Response**
Are you submitting this manuscript to a special series or article collection?	No
**Experimental design and statistics**<br><br>Full details of the experimental design and statistical methods used should be given	Yes

in the Methods section, as detailed in our Minimum Standards Reporting Checklist. Information essential to interpreting the data presented should be made available in the figure legends.<br><br>Have you included all the information requested in your manuscript?	
**Resources**<br><br>A description of all resources used, including antibodies, cell lines, animals and software tools, with enough information to allow them to be uniquely identified, should be included in the Methods section. Authors are strongly encouraged to cite Research Resource Identifiers (RRIDs) for antibodies, model organisms and tools, where possible.<br><br>Have you included the information requested as detailed in our Minimum Standards Reporting Checklist?	Yes
**Availability of data and materials**<br><br>All datasets and code on which the conclusions of the paper rely must be either included in your submission or deposited in publicly available repositories (where available and ethically appropriate), referencing such data using a unique identifier in the references and in the "Availability of Data and Materials" section of your manuscript.<br><br>Have you have met the above requirement as detailed in our Minimum Standards Reporting Checklist?	Yes

Manuscript

Click here to access/download;Manuscript;Genome sequence of the German Shepherd (revised).docx

~~-~~Canfam_GSD*: De novo* chromosome-length genome assembly of the German Shepherd Dog (Canis lupus familiaris) using a combination of long reads, optical mapping and Hi-C~~***De novo*** **genome assembly of German Shepherd Dog (*****Canis lupus familiaris*****)**~~

Matt A. Field[1,2]*, Benjamin D. Rosen[3*], Olga Dudchenko[4,5,6*], Eva K.F. Chan[7,8], Andre E. Minoche[7], Richard J. Edwards[9]~~K,~~ Kirston Barton[7,8], Ruth J. Lyons[7], Daniel Enosi Tuipulotu[9], ~~Richard J. Edwards[9],~~ Vanessa M. Hayes[7,8,10], Arina Omer[4,5], Zane Colaric[4,5], Jens Keilwagen[11], Ksenia Skvortsova[7], Ozren Bogdanovic[7,9], Martin A. Smith[7,8], Erez Lieberman Aiden[4,5,6,12,13], Timothy P.L. Smith[14], Robert A. Zammit[15], J. William O. Ballard[9§]

1 Centre for Tropical Bioinformatics and Molecular Biology, Australian Institute of Tropical Health and Medicine, James Cook University, Cairns, QLD 4878, Australia. matt.field@jcu.edu.au

2 John Curtin School of Medical Research, Australian National University, Canberra, ACT 2600, Australia. matt.field@jcu.edu.au

3 Animal Genomics and Improvement Laboratory, Agricultural Research Service USDA, Beltsville, MD 20705. ben.rosen@usda.gov

4 The Center for Genome Architecture, Department of Molecular and Human Genetics, Baylor College of Medicine, Houston, TX, USA. Olga.Dudchenko@bcm.edu, Arina.Omer@bcm.edu, Zane.Colaric@bcm.edu, erez@erez.com

5 Department of Computer Science, Rice University, Houston, TX, USA. Olga.Dudchenko@bcm.edu, Arina.Omer@bcm.edu, Zane.Colaric@bcm.edu, erez@erez.com

6 Center for Theoretical and Biological Physics, Rice University, Houston, TX, USA. Olga.Dudchenko@bcm.edu, erez@erez.com

7 Garvan Institute of Medical Research, Darlinghurst, NSW, Australia.

a.minoche@garvan.org.au, e.chan@garvan.org.au, k.barton@garvan.org.au, r.lyons@garvan.org.au, v.hayes@garvan.org.au, o.bogdanovic@garvan.org.au, k.skvortsova@garvan.org.au, m.smith@garvan.org.au

8 Faculty of Medicine, University of New South Wales Sydney, Kensington, NSW, Australia. k.barton@garvan.org.au, e.chan@garvan.org.au

9 School of Biotechnology and Biomolecular Sciences, University of New South Wales, Sydney NSW 2052, Australia. D.enosi@unsw.edu.au, Richard.edwards@unsw.edu.au, o.bogdanovic@garvan.org.au, w.ballard@unsw.edu.au

10. Central Clinical School, University of Sydney, Camperdown, NSW, Australia. vanessa.hayes@sydney.edu.au

11 Julius Kühn-Institut**,** Erwin-Baur-Str. 27 06484 Quedlinburg, Germany. jens.keilwagen@julius-kuehn.de

12 Broad Institute of MIT and Harvard, Cambridge, MA, USA. erez@erez.com

13 Shanghai Institute for Advanced Immunochemical Studies, ShanghaiTech University, Shanghai, China. erez@erez.com

14. US Meat Animal Research Center, Agricultural Research Service USDA, Clay Center, NE 68933. Tim.Smith2@usda.gov

15. Vineyard Veterinary Hospital, 703 Windsor Rd, Vineyard, NSW, 2765. Robert@vineyardvet.com.au

[§]**Correspondence address**. J. William O. Ballard: School of Biotechnology and Biomolecular Sciences, University of New South Wales, Sydney, NSW, 2052, Australia. Tel: +61-293853780; Email: w.ballard@unsw.edu.au. Tel: +61 2 93852021; Fax: +61 293851483.

***** These authors contributed equally to this work.

Abstract

Background

The German Shepherd Dog (GSD) is one of the most common breeds on earth and has been bred for its utility and intelligence. It is often first choice for police and military work, as well as protection, disability assistance and search-and-rescue. Yet, GSD's are well known to be afflicted with a range of genetic diseases that can interfere with their training. Such diseases are of particular concern when they occur later in life, and fully trained animals are not able to continue their duties.

Findings

Here, we provide the draft genome sequence of a healthy German Shepherd female as a reference for future disease and evolutionary studies. We generated this improved canid reference genome (CanFam_GSD) utilising a combination of Pacific Bioscience, Oxford Nanopore, 10X Genomics, Bionano, and Hi-C technologies. The GSD assembly is approximately 80 times as contiguous as the current canid reference genome (20.9 Mb vs 0.267 Mb contig N50), containing far fewer gaps (306 vs 23,876) and fewer scaffolds (429 vs 3,310) than the current canid reference genome CanFam v3.1. Two chromosomes (4 and 35) are assembled into single scaffolds with no gaps. Benchmarking Universal Single-Copy Orthologs analyses of the genome assembly results show 93.0% of the conserved single-copy genes are complete in the GSD assembly compared to 92.2% for CanFam v3.1. Homology-based gene annotation increases this value to about 99%. Detailed examination of the evolutionary important pancreatic amylase region reveals there are most likely seven copies of the gene indicative of a duplication of four ancestral copies and the disruption of one copy.

Conclusions

GSD genome assembly and annotation were produced with major improvement in completeness, continuity and quality over the existing canid reference. This resource will enable further research related to canine diseases, the evolutionary relationships of canids, and other aspects of canid biology.

Key Words: Hi-C,; long read sequencing, optical mapping, de novo genome assembly, canine hip dysplasia, DNA Zoo,

canine hip dysplasia.

Introduction

Arising from wild grey wolves on the Eurasian continent over 15,000 years ago, the dog (*Canis lupus familiaris*) was the first species to be domesticated [1-3]. Mitochondrial DNA evidence suggests that seats of canine domestication may have been China [3], Europe [4], and the Middle East [5]. Since domestication, canids have undergone thousands of years of selective breeding, giving rise to a myriad of phenotypic variants. However, most modern breeds are less than 200 years old and are of European ancestry [6, 7].

The German Shepherd Dog (GSD) is a medium to large working dog and was developed from common livestock dogs late in the 19th century in continental Europe [7]. In 1899, Captain Max von Stephanitz attended a dog exhibition event and was shown a dog named *Hektor Linksrhein*. *Hektor* satisfied what von Stephanitz believed a working dog should be, and he bought him immediately. After purchasing the dog, von Stephanitz changed his name to *Horand von Grafrath* and founded the Verein für Deutsche Schäferhunde (Society for the German Shepherd Dog). *Horand* was declared to be the first GSD and was the first dog added to the society's breed register [8]. Von Stephanitz is reported to have kept a strong reign over the early

development of the GSD and this likely resulted in a degree of inbreeding. However, it also enabled the fixation of qualities that are now features of the breed.

Subsequent roles for the GSD, which included guarding and police work, contributed to selective breeding for larger and more confident dogs [9]. Over recent decades, further selection towards characteristics deemed desirable in the show-ring have further altered the GSD conformation [10]. Perhaps the ~~best~~ best-known disease is Canine Hip Dysplasia (CHD), which is a complex disease combining genetic and environmental factors. Genetic factors, such as shallow acetabulum, subluxation, and poorly forming femoral heads will manifest early in a dog's life if severe. Environmental factors such as overweight or poor exercise area (many stairs and much jumping in juvenile life) will manifest in later life. Other common health problems include elbow dysplasia, bloat, degenerative myelopathy, epilepsy, haemophilia, diabetes, inflammatory bowel disease, and a variety of cancers including osteosarcoma, lymphoma, and melanoma [11-16].

In Australia, early imports of GSD’s were known to have arrived from 1904. In October 1928, the Federal Government of Australia placed an importation ban on the breed, which was enforced in 1929. During the course of the import ban, which was to stretch for another 43 years, few imports were smuggled into the country. The import ban was lifted in 1972 with some restrictions remaining until 1976. With the lifting of the import ban, German, New Zealand, England –and some American dogs were imported into Australia, and the breed enjoyed a surge in popularity. Currently, the GSD is the largest breed (purebred) dog population in Australia [17].

The aim of this study is to is to provide a high resolution long read *de novo* assembly of the genome of a GSD female that is free of known genetic diseases (**Figure 1**). This *de novo* genome assembly will be an invaluable tool for advancing knowledge of both simple and polygenic genetic diseases and also the evolutionary affinities of the GSD.

Figure 1 title: *Nala* the German Shepherd

Figure 1 legend: The female selected was *Nala*, or formally "Jonkahra Nala" (Australian Registration #2100398550). *Nala* was born in 2013 and she is free of all known genetic diseases. Her sire was imported from Germany, and her dam is from Australian lines.

Results

Workflow

The genome was assembled using Pacific Bioscience (PacBio) Single Molecule Real-Time (SMRT) sequencing, Oxford Nanopore (ONT) PromethION sequencing, 10X Genomics Chromium genome sequencing with Bionano and Hi-C scaffolding (**Supplementary Figure 1**). Contigs were assembled using SMRT and ONT sequencing [18] and then polished [19, 20] to minimise error propagation (see ***Long rRead gGenome assembly for details***). The assembled sequence contigs were scaffolded sequentially using 10X linked-reads, Bionano optical mapping and Hi-C proximity ligation scaffolding. To increase the contiguity of the assembly we used the SMRT and ONT reads to fill gaps, which was then followed by a final round of polishing. Homology-based gene prediction was performed using *Canis lupus familiaris* and eight related mammals. The resulting chromosome-length genome assembly and its gene annotation was deposited to NCBI with accession number GCA_008641055.1. In addition to the nuclear genome, the mitochondrial genome was assembled and has been uploaded to GigaScience for immediate download. It also has been deposited to NCBI and is

Formatted: Left, None, Don't keep with next

Formatted: Font: Not Italic

Formatted: Font: Not Italic

being processed. Finally, comparisons to the canine genome of the boxer (CanFam3.1) were made [21].

Formatted: Font color: Text 1

Formatted: Font: -webkit-standard, Not Bold, Not Italic, Font color: Black, English (Australia)

Assembly stats / completeness

The final submission contains 2,407,291,559 total bp (2,401,147,102 ungapped), 429 scaffolds with a contig N50 length of 20.9 Mb and a scaffold N50 length of 64.3 Mb. The full-length chromosome scaffolds in the assembly accounted for 98.3% of the genome with only 0.95% of all sequence not aligning to a CanFam3.1 chromosome. Evaluation by Benchmarking Universal Single-Copy Orthologs (BUSCO v3.0.2b [22], short mode, implementing BLAST+ v2.2.31 [23], HMMer v3.2.1 [24], AUGUSTUS v3.3.2 [25] and EMBOSS v6.6.0) against Laurasiatheria_ob9 data set (n=6,253) indicated that 93.0% of the conserved single-copy genes were complete (**Table 1, Supplementary Table 1, Supplementary Figure 2**). Each analysis step in assembly, scaffolding and polishing improved scaffold N~~G~~50 and/or BUSCO scores, consistent with improving assembly quality (**Supplementary Table 1, Supplementary Figure 2**). BUSCO predictions are sensitive to changes in sequence and assembly size, with scaffolding and polishing causing losses as well as gains (**Supplementary Table 1**). Compiling BUSCO results across all assembly stages (BUSCOMP v0.8.0) reveals that at least 6,085 (97.3%) are present and complete in the assembly, with only 118 genes (1.9%) not found at any stage.

Additional k-mer analysis of the final assembly was performed using KAT v2.4.2 [26]. KAT comp was used to compare k-mer frequencies from the 10x reads (16 bp barcode trimmed from read 1) with their copy number in the assembly. This comparison revealed no sign of missing data nor large duplications, including retention of haplotigs (**Supplementary Figure 3**).

Comparison to CanFam3.1

The GSD assembly was compared to the current reference genome CanFam3.1. Results are summarised in Table 1.

Table 1: G~~Nuclear g~~enome assembly and annotation statistics for GSD assembly vs CanFam3.1

**Statistic**	**GSD**	**CanFam3.1**
Total sequence length	2,407,291,559	2,410,976,875
Total ungapped length	2,401,147,102	2,392,715,236
Number of contigs	735	27,106
Contig N50	20,914,347	267,478
Contig L50	37	2,436
Number of scaffolds	~~429~~410	3,268~~310~~
Scaffold N50	64,346,267	63,241,923~~45,876,610~~
Scaffold L50	15	15
Number of gaps	306	23,876
BUSCO complete (genome)	93.0% (91.6% single copy, 1.4% duplicate copy)	92.2% (91.1% single copy, 1.1% duplicate copy)
BUSCO fragmented (genome)	3.6%	4.0%
BUSCO missing (genome)	3.4%	3.8%
BUSCO complete (annotation)	98.9% (96.5% single copy, 2.4% duplicate copy)	95.1% (94.1% single copy, 1.0% duplicate copy)
BUSCO fragmented (annotation)	1.0%	1.9%

Formatted: Font: (Default) Times New Roman
Formatted: Left, Line spacing: single
Formatted: English (Australia)
Formatted Table
Formatted: Left
Formatted Table

BUSCO missing (annotation)	0.1%	3.0%

The GSD assembly offers improvements over CanFam3.1 using a wide variety of metrics. The GSD assembly has a contig N50 that is almost 80 times greater than CanFam3.1, contains 78 times fewer gaps, and 2,881 fewer scaffolds. BUSCO results on the genome also indicate an improvement in the GSD assembly with 47 more complete genes (25 fewer fragmented genes and 22 fewer missing genes).

Based on the existing CanFam3.1 annotation and the GSD annotation provided by GeMoMa [27], the longest full-length transcript per gene was selected to avoid an overestimation of duplicated genes by BUSCO v3.02. Comparing the BUSCO statistics for the annotations, a clear improvement from 95.1% to 98.9% complete single copy orthologs could be observed.

Variation relative to CanFam3.1

All 39 full-length chromosomes in the final assembly were aligned to the corresponding chromosomes in CanFam3.1 using MUMmer4 [28]. Single-nucleotide polymorphisms (SNPs) ~~, single-nucleotide variations (SNVs)~~ and small indels (deletions and insertions <50bp) were called using MUMmer4 call-SNPs module. In total 3,137,227 SNVs and 5,111,356 small indels were detected (~~**Supplemental**~~ **Table 2**). Copy number (CNV) and structural variants (SV) were called using svmu (v0.2) available at https://github.com/mahulchak/svmu. Variants greater than 100 bp were extracted resulting in a total of 66,673 total CNV/SVs. By variant type, this was broken down into 39,742 CNVs, 13,552 insertions, 13,150 deletions, and 229 inversions (~~**Supplemental**~~**Supplementary** **Table 3**).

~~*Pacreatic*~~*Pancreatic* ***amylase*** **(AMY2B)** ***analysis***

AMY2B is important in canid evolution, with variation in copy number being linked to starch diet adaptations in ancient European dogs. Ollivier *et al.* looked at both ancient and modern dogs finding the expansion as early as the seventh century with between 4-16 copies in modern dogs [29]. No long reads were found to span the entire region. The longest read in the region covered no more than three complete~~three plus~~ copies, with four copies ultimately submitted in the GSD assembly. Further examination of this region was attempted using both the Bionano ~~genome~~ optical map and read depth analysis from the SMRT and ONT reads (**Supplementary File 1)**. The read depth results estimate that there are between 7-8 copies of the gene while the Bionano map indicated the most likely copy number is seven~~7~~ (**Figure 2**). For the Bionano analysis, single molecules of Bionano data were *de novo* assembled using a haplotype-aware algorithm (**Supplementary File 2**) to obtain a phased consensus genome map set. Alignment of the resulting genome maps to the GSD assembly identified two homozygous alleles (Map ID #1111 & #1112) spanning the *AMY2B* region as predicted by GeMoMa (**Supplementary Figure 4**). The alignment shows a ~11 kb "insertion", flanked by DLE1 enzymatic labels at positions 47,325,815 and 47,333,432 of NALACHR6.01, suggesting that this fragment, which is upstream of the *AMY2B* region, is either lost or collapsed in the GSD assembly. Additionally, the region flanked by DLE1 labels at 47,341,704 and 47,396,280, which encompasses three of the GeMoMa-predicted *AMY2B* copies, are tandemly duplicated, suggesting seven possible copies of *AMY2B* in *Nala*. The two alleles are supported by an average of 40X and 23X single long molecules, with 12 spanning the full repeat structure, of which eight also span the 11 kb insertion. It should be noted here that, due to sequence similarity between the four GeMoMa-predicted *AMY2B* copies and associated inherent alignment ambiguities, it is unclear exactly which repeat units are duplicated.

Formatted: Font: Italic

Compared to CanFam3.1, the pair of homozygous genome map alleles show an insertion of ~ 100 kb flanked by DLE1 labels at positions 46,954,644 and 46,999,962 of Chr6 (**Supplementary Figure 5**), which is indicative of a complete loss of the *AMY2B* locus in CanFam3.1.

To better determine whether Bionano data supports seven or eight copies of the *AMY2B* repeat, we compared the two genome map alleles against two synthetic sequence constructs containing either seven (amy2b_dom7copyext) or eight (amy2b_dom8copyext) copies of the 14,862 bp *AMY2B* repeat with the highest read depth support (namely the third copy) from the GSD assembly, flanked by ~401.5 kb sequences assembled from SMRT and ONT reads (**Supplementary File 1**). Alignment results confirmed the presence of seven repeat units, showing a perfect alignment to the seven-copy sequence construct (**Figure 2A**), but a "deletion" of one repeat unit relative to the eight-copy construct (**Figure 2B**).

Figure 2 Title: Bionano genome map alleles aligned to hypothetical sequence constructs

Figure 2 Legend: The hypothetical sequence constructs (green bars) contain either seven (labelled **amy2b_dom7copyext**) or eight (labelled **amy2b_dom8copyext**) copies of the repeat unit (highlighted by coloured boxes within the green bar and numbered in white font). Dark blue and yellow vertical lines on the sequence contig and consensus map indicate matching and non-matching DLE1 enzymatic labels respectively.

Olfactory receptor and Keratin cluster analyses

Correct annotation of the olfactory and keratin clusters in canines has been problematic but it is important for research on canid health and evolution [21, 30, 31]. Dogs are macrosmatic animals that rely highly on their sense of smell. Yet, the molecular basis of such prominent

chemosensory capacities remains largely unknown. The ability to detect and discriminate the multitude of odors in vertebrates is mediated by a superfamily of G-protein-coupled olfactory receptor (OR) proteins [32]. Based on the description ~~(product)~~ in the reference annotations, we filtered all mRNAs of the references that contain in their description the regular expression “olfactory receptor”. We then extracted the number of mRNAs and genes per reference organism. Subsequently, we used the IDs to filter the GSD annotation and counted the number of predicted ~~ions (=~~mRNAs~~)~~ and genes. This procedure identifies 1250 mRNAs and 933 genes in the GSD and 849 mRNAs and 804 genes in the boxer. Quignon et al. [30] identified five amino acid patterns characteristic of ~~of~~ ORs in the canine genome and retrieved 1,094 dog genes (872 genes and 222 pseudogenes).

Keratins are filament proteins of the epithelial cytoskeleton and are essential for normal skin homeostasis. Over time the genes encoding keratins have undergone multiple rounds of duplication with high similarity between different keratin paralogs [31]. Analogously to the olfactory receptor study, we filtered all mRNAs of the references that contain in their description the keyword “keratin \d”. This procedure identifies 118 mRNAs and 83 genes in the GSD and 73 mRNAs and 55 genes in the boxer. Balmer et. al. [31] investigated the National Center for Biotechnology Information (NCBI) (dog annotation release 103) gene predictions for the canine gene clusters to RNA-seq data that were generated from adult skin of five dogs and adult hair follicle tissue of one dog and annotated 61 putatively functional keratin genes in the dog.

Discussion

Concerns for the health welfare of the GSD have been widely aired [33, 34]. The GSD had the highest number of published predispositions to inherited diseases overall among the fifty most

commonly registered Kennel Club breeds and had the second-highest number of disorders exacerbated by conformation, exceeded only by the Great Dane [35]. The British KC Breed Watch system categorises the GSD as a Category Three breed "requiring particular monitoring and additional support" and considered to be more susceptible to developing specific health conditions associated with exaggerated conformation. Breed Watch points of concern include cow hocks, excessive turn of stifle, nervous temperament, sickle hock, and weak hindquarters [36].

The high-quality genome assembly will ~~facilitate~~ advance knowledge of breed specific diseases such as CHD and extend to issues related to canine personality. The severity of CHD depends on both genetic and environmental factors. In GSD~~'~~s, the heritability (h^2) estimates have varied from 0.1 to 0.6 [37]. To date, different study populations and methods affect the results substantially, as the reported quantitative trait locus (QTL) association and candidate genes are inconsistent between studies [38-40]. While boxers are prone to CHD the hip scores of *Tasha*, used for CanFam, are unknown. Further, GSD ~~breed~~ specific SNP~~'~~s as well as significant CNV~~'~~s and SV~~'~~s are difficult to detect. In a cohort containing over 10 000 behaviorally tested GSD and Rottweiler dogs Saetre et al. [41] examined how traits are transmitted between generations. In both breeds, the pattern of co-inheritance was found to be similar for a broad personality trait previously named shyness–boldness with heritability estimated to be 0.25 in the two breeds. Currently, the underling genes involved in these behaviors are not known.

The assembly is expected to enable the selection of GSDs for particular duties including police work where their sensitive nose is frequently used to discriminate odors. Robin et al. [42] analyzed the nucleotide sequences of 109 OR genes (102 genes and seven pseudogenes)

in six different breeds including GSD's. In this study, they showed that OR genes are highly polymorphic, with a mean of one SNP per 577 nucleotides. However, the degree of polymorphism observed is highly variable, with some OR genes having few if any SNPs and others being highly polymorphic (1 SNP/122 nt). Yang et al. [43] conducted a preliminary study of 22 SNPs from the exonic regions of 12 OR genes in GSDs and found a significant correlation between SNP genotypes of OR genes and olfactory abilities of dogs.

We envisage these data will also facilitate understanding of the evolution of dog breeds and canids in general. The evolutionary position of the GSD among extant breeds is not firmly established. The Federation Cynologique International places it in Group 1 as part of the Herding group. Bigi et al. [44] hypothesiszed that the German shepherd dog was closely related to the Czechoslovakian Wolfdog. More recently Parker et al. [6] proposed that the GSD is distinct from other herding breeds and in a clade along with the French Berger Picard, New Hampshire Chinook, Peruvian Hairless and Mexican Xoloitzcuinti.

Conclusions

This *de novo* genome assembly and annotation will be an invaluable tool for advancing knowledge of breed specific diseases and the evolutionary affinities of the GSD. Here, we present an improved canid genome assembly and annotation relative to CanFam 3.1.

Methods

DNA extraction, sequencing, and scaffolding

Sampling: Nala the German Shepherd Dog

In selecting an animal for the project, it was considered essential to select a female that had been cleared, as much as possible, of any recognis~~z~~able inherited conditions. The animal needed to display all the hallmarks of a good quality representative of the breed but need not necessarily be a show-winning specimen. *Nala* is an easy going and approachable 5.5 year old female (born 05 December 2013) and a treasured family pet that showed typical appearance for a GSD. She has had no sign of hip dysplasia, that appears in GSD (**Supplementary Figure 6**), or any other known genetic diseases. *Nala* had a combined hip score of three~~3~~ (one~~1~~ on the left hand side and two~~2~~ on the right hand side) when the x-ray was taken at five~~5~~ years of age: each hip was measured on a 0 – 53 scale, wih a total of 106 being crippling. The score of three~~3~~ is well below the current Australian average of nine~~9~~ for GSD's. She is registered with the Australian National Kennel Council (2100398550) with her dam from Australian bred lines and sire imported from Germany. Her dam and sire remain healthy aging adults without disease. *Nala's* dam has seven ~~7~~ progeny radiographed from four~~4~~ sires with no failures. Her sire had 31 progeny radiographed from 13 different dames resulting in four~~4~~ failures and 27 passes recorded for the GSD National Council hip scheme. In the Australian 53 point scoring scheme a pass is no more than eight ~~8~~ in any one hip, no point gets a three~~3~~ and not more than 16 in total.

Pacific Bioscience Single Molecule Real-Time (SMRT) sequencing

Genomic DNA was prepared from 1–2 mL~~l~~ of fresh blood using the genomic-tip 100/G kit (Qiagen, Hilden, Germany). This was performed with supplemental RNase (Astral Scientific, Taren Point, Australia) and proteinase K (NEB, Ipswich, MA, USA) treatment, as per the manufacturer's instructions. Isolated gDNA was further purified using AMPure XP beads (Beckman Coulter, Brea, CA, USA) to eliminate sequencing inhibitors. DNA purity was calculated using a Nanodrop spectrophotometer (Thermo Fisher Scientific), and molecular

integrity was assessed using pulse-field gel electrophoresis. DNA integrity was assessed by the Sage Science Pippin Pulse. A 0.75% KBB gel was run on the 9hr 10-48kb (80 V~~v~~) program. DNA ladder used was the Invitrogen 1kb Extension DNA ladder (cat 10511-012). 150ng of DNA was loaded on the gel.

We generated two libraries that were size selected on Sage BluePippin gels (Sage Science, Beverly, MA, USA). Libraries were sequenced on Sequel machines with 2.0 chemistry recording 10 h movies. Sequencing was conducted at the Ramaciotti Center for Comparative Genomics at University of New South Wales (TOW5157A1, 15 SMRT cells with a total polymerase read length 108.48 Gb~~B~~) and at the Arizona Genomic Institute, University of Arizona (four SMRT cells with a total of 11Gb of data: NOTE: short read lengths were due to DNA shearing of the DNA during shipping from Australia to Arizona).

Oxford Nanopore (ONT) PromethION sequencing

DNA (1 µg) was prepared for ONT sequencing using the 1D genomic DNA by ligation kit (SQK-LSK109, ONT) according to the standard protocol. Long fragment buffer was used for the final elution to exclude fragments shorter than 1000 bp. In total, 119 ng of adapted DNA was loaded onto a FLO-PRO002 PromethION flow cell and run on an ONT PromethION sequencing device using MinKNOW (18.08.2) with MinKNOW core (v1. 14.2).

Base-calling was performed after sequencing with the GPU-enabled guppy basecaller (v3.0.3) using the PromethION high accuracy flip-flop model with config ‘dna_r9.4.1_450bps_hac.cfg’.

10X Genomics Chromium sequencing

DNA was prepared following the protocol described above for SMRT sequencing. A 10X GEM library was barcoded from high-molecular-weight DNA according to the manufacturers recommended protocols. Protocol used was the Chromium Genome Reagent Kits v2 User Guide, manual part number CG00043 Rev B available here: https://support.10xgenomics.com/genome-exome/library-prep/doc/user-guide-chromium-genome-reagent-kit-v2-chemistry. QC was performed using LabChip GX (PerkinElmer, MA, USA) and Qubit 2.0 Flurometer (Life Technologies, CA, USA) at the Kinghorn Centre for Clinical Genomics. The library was run on a single lane of a v2 patterned flowcell. Paired-end sequencing with 150 bp read length was performed using the Illumina HiSeq X within the Kinghorn Centre for Clinical Genomics at the Garvan Institute of Medical Research, Sydney, Australia.

DNA m~~M~~ethylome

To explore the regulatory landscape of the GSD, we performed whole genome bisulfite sequencing [45] on genomic DNA extracted from whole blood. In concordance with other adult vertebrates [46, 47], the GSD genome displays a typical bimodal DNA methylation pattern with over 60% of CpG dinucleotides being methylated at levels higher than 80% (hypermethylated) ~~–~~and 12% of CpG dinucleotides being methylated at 20% or lower (hypomethylated). ~~over 60% of CpG dinucleotides are largely methylated (>80%) with ~12% being largely unmethylated (<20%).~~ Next, to determine the number and genomic distribution of putative regulatory regions, we segmented the methylome into unmethylated regions (UMRs) and low-methylated regions (LMRs), using the MethylSeekR algorithm [48]. UMRs are fully unmethylated and largely coincide with CpG island promoters whereas LMRs display partial DNA methylation, which is characteristic of distal regulatory elements such as

enhancers in other mammalian models [49]. These analyses resulted in the identification of ~ 21,000 UMRs and ~53,000 LMRs in line with previously reported numbers of promoters and enhancers [48, 50] (**Supplementary Figure 78**).

Bionano optical mapping

High molecular weight (HMW) DNA was isolated from fresh blood (stored at 4°C) using the Bionano Prep Blood DNA Isolation Protocol (Bionano Genomics (BNG), Document #30033 revision C). Briefly, after lysing the red blood cells, white blood cells were recovered and embedded in agarose plugs. These plugs were subjected to Proteinase K (Qiagen Cat# 158920) digestion for two rounds (2 hours, then overnight) at 50°C. Following extensive washing as prescribed in the protocol, the plugs were melted and treated with GELase enzyme (Epicentre, Catalog # G31200). The resulting HMW DNA was subjected to drop dialysis, left to equilibrate at room temperature for four days and was then quantified using the Qubit Broad Range dsDNA Assay Kit (Thermo Fisher Scientific).

HMW DNA (~190 ng/µL) was labelled (BNG, Part #20351) at DLE-1 recognition sites, following the Bionano PrepTM Direct Label and Stain Protocol (BNG, Document #30206 revision C). Labelled DNA was loaded directly onto Bionano Saphyr Chips (BNG, Part #20319), without further fragmentation or amplification, and imaged using a Sahphyr instrument to generate single-molecule optical maps. Multiple cycles were performed to reach an average raw genome depth of coverage of 190X.

Hi-C chromosome length scaffolding

The Bionano assembly was further scaffolded to chromosome-length by the DNA Zoo following the methodology described here: www.dnazoo.org/methods. Briefly, an *in situ* Hi-C

library was prepared [51] from a blood sample of a purebred male individual named Tydus (American Kennel Club Registration DN5364660) provided by the Cornell Veterinary Biobank and sequenced to 29Xx coverage (assuming 2.6 Gb genome size).

Genome Assembly Workflow

Long rRead gGenome assembly

The SMRT and ONT reads were corrected and assembled with the Canu assembler (Canu, RRID:SCR_015880) [18] (v1.8.0). The resulting contigs were polished by aligning the raw reads to the assembly and correcting the sequencing errors using two rounds of Arrow polishing [19]. There were ~10 million fixes in the first round and ~284,000 fixes k in the second. The assembled GSD genome, with a total length of 2.39 Gb, consisted of 1389 contigs with an N50 length of 15.68 Mb. Following the Arrow polishing there were 1389 sequences, total length 2.39 Gb (including 111 repeats of total length 13,145,025 bp) with no bubbles. There were 2,560,498 unassembled sequences of total length 17,998,063,955 bp.

10X Chromium linked-reads

The Arrow-polished SMRT/ONT assembly was scaffolded using GSD 10X linked-reads as in ARCS [52]. The 10X data was aligned using the linked-read analysis software provided by 10X Genomics, Long Ranger, v2.1.6 (https://www.10xgenomics.com/). Misaligned reads and reads not mapping to contig ends were removed, and all possible connections between contigs were computed keeping best reciprocal connections. Finally, contig sequences were joined, spaced by 10kb with stretches of N's, and if required reverse complemented (**Supplementary File 3**). In total 128 connections between the SMRT/ONT contigs could be established increasing the assembly N50 length by from 15.46 Mb by 4.6 Mb to 20.06 Mb **(Supplementary File 3).**

Polishing round 1

To further improve the assembly, another round of polishing was performed by aligning the Illumina short reads from the 10X Chromium sequencing to the assembly using minimap2 [53] (v2.16) and correcting the sequencing errors using Racon [54] (v1.3.3).

Optical mapping for super-scaffolding using Bionano data

Single-molecule optical maps were filtered on minimum molecule length of 150 kb and minimum of nine label sites per molecule. *De novo* assembly of single molecules into consensus maps were performed using the Bionano Solve (v3.2.2_08022018) software with aligner RefAligner (7782.7865rel) [55, 56]. Assembly was "haplotype-unaware" such that heterozygous alleles were collapsed into haploid representation. In all, approximately two million single-molecules with N50 of 220 kb were assembled into 1245 optical genome maps with N50 of 3.1 Mb. The final assembly was in CMAP format (v0.2).

This genome map set was used to scaffold the sequence contigs using BNG's Hybrid Scaffold pipeline (v10252018). In brief, the 1261 sequence contigs were *in silico* digested based on the DLE-1 motif (CTTAAG) creating sequence maps (CMAP). Sequence maps were then aligned to the assembled optical maps based on DLE-1 labels using RefAligner. Discrete sequence maps that can be linked via a Bionano genome map were scaffolded. Alignments indicating conflict between the sequence and optical maps, and hence suggestive of mis-assembly, were resolved. Specifically, optical maps supported by at least ten single molecules at the conflict site were indicative of sequence mis-assembly, and so the sequence map would be "cut" (split) at the conflict point. In contrast, insufficient single molecule support for the optical map was indicative of optical map assembly error, and so the optical

map would be "cut" at the conflict site. Details of the method are provided in the Bionano Solve Theory of Operation: Hybrid Scaffold (Document #30073). Following hybrid scaffolding, 21 arbitrary 10 kb N-gaps (introduced during the sequence assembly process) were re-sized based on estimated inter-label distances from the optical maps. In all, 160 sequence contigs were hybrid-scaffolded into 109 hybrid scaffolds with N50 of ~46.3 Mb. The remaining 1,004 sequence contigs with an N50 of ~78.8 kb could not be scaffolded either because they are too short (< 100 kb) for hybrid-scaffolding with Bionano maps or because they did not align to any optical maps.

Chromosome-length assembly using Hi-C data

The Hi-C data was processed using Juicer [57], and used as input into the 3D-DNA pipeline [58] to produce a candidate chromosome-length genome assembly. We performed additional finishing on the scaffolds using Juicebox Assembly Tools [59]. **Figure 3** shows the contact matrices generated by aligning the Hi-C data set to the genome assembly before the Hi-C upgrade (on the left), and after Hi-C scaffolding (on the right). The matrices are visualised in Juicebox.js, a cloud-based visualisation system for Hi-C data [60] and are available for browsing at multiple resolutions on www.dnazoo.org/assemblies/Canis_lupus_familiaris_German_Schepherd.

Figure 3 title: GSD assembly before and after Hi-C correction

Figure 3 legend: Contact matrices (visualised in Juicebox.js) comparing the GSD assembly before and after the chromosome-length Hi-C upgrade.

Gap filling

After scaffolding and correction, all raw SMRT and ONT reads were aligned to the assembly with Minimap2 (v2.16) (-ax map-pb/map-ont) and used by PBJelly (pbsuite v.15.8.24) [61] to fill gaps. It was able to completely close 210 gaps, increasing contig N50 to the final figure of 20.9 Mb.

Polishing rRound 2

Following scaffolding, another round of polishing was done to further improve the assembly. Polishing was performed by aligning the Illumina short reads from the Chromium sequencing to the assembly using Long Ranger v2.2.2 and correcting the SNPs and indels using Pilon [20].

Final cleanup

The Pilon-polished genome underwent a final scaffold clean-up to generate a high-quality core assembly, remove low-coverage artefacts and haplotig sequences, and annotate remaining scaffolds with potential issues.

Low-coverage filter

The TOW5157A1 library PacBio subreads (12.5M subreads; 108Gb) were mapped onto the Nala_canu_arrow2_10x_racon_bionano_HiC_pbjelly_pilon assembly using Minimap2 v2.16 (-ax map-pb --secondary=no) [53]. Initial read depth analysis was performed with BBMap v38.51 pileup.sh (https://github.com/BioInfoTools/BBMap/). Any scaffolds with median coverage less than three3 (e.g., less than 50% of the scaffold covered by at least three reads) were filtered out as lLow--cCoverage scaffolds. Of the 1,057 Pilon-polished scaffolds, 220 scaffolds were removed in the initial lLow-coverage filter, leaving 837 scaffolds.

Formatted: Justified

Purge Haplotigs analysis - round 1

~~Subreads were re-mapped on to the remaining 837 scaffolds and processed w~~Subread were re-mapped on the remaining 837 scaffolds and processed with PurgeHaplotigs v20190612 [62] (implementing Perl v5.28.0, BEDTools v2.27.1 [63], R v3.5.3, and SAMTools v1.9 [64]). Based on the PurgeHaplotigs depth histogram, low-, mid- and high-depth thresholds were set to 5X, 30X and 80X. Any scaffolds with <80% at diploid read depth were identified by PurgeHaplotigs for reassignment. Scaffolds with 80%+ bases in the low/haploid coverage bins and 95%+ of their length mapped by PurgeHaplotigs onto another scaffold were filtered as haplotigs or assembly artefacts. Any other scaffolds with 80%+ low coverage bases were filtered as Low Coverage. This analysis resulted in a further 11 scaffolds filtered for low coverage and 268 filtered as haplotigs or assembly artefacts, leaving 558 scaffolds.

Purge Haplotigs analysis - round 2

Subreads were re-mapped on to the remaining 558 scaffolds resulting in ~~and~~ a further 128 scaffolds ~~were~~ filtered as haplotigs or assembly artefacts leaving 430 scaffolds~~for a second round of slightly more stringent PurgeHaplotigs analysis~~. No additional scaffolds with 80%+ low coverage bases were identified. Any scaffold with 80%+ bases in the low/haploid coverage bins were filtered as haplotigs or assembly artefacts. Scaffolds with 20%+ diploid coverage were marked as retention as probable diploids. Scaffolds with <20% diploid coverage and 50%+ high coverage were marked as probable collapsed repeats. A single remaining ~~Scaffold~~ scaffold marked as JUNK by PurgeHaplotigs (over 80% low/high coverage) was also filtered as a probable artefact.

Purge Haplotigs analysis - round 3

Subreads were re-mapped on to the remaining 430 scaffolds for a third round of PurgeHaplotigs analysis. No further scaffolds were identified for filtering.

CanFam3.1 c~~C~~hromosome m~~M~~apping

The CanFam v3.1 reference genome was downloaded from Ensembl (Release 97, download date 05/08/2019). Full length chromosomes were renamed with a CANFAMCHR prefix and used for reference mapping. The final *Nala* genome assembly was mapped onto the CanFam3.1 reference genome using Minimap2 v2.16 [53] (-x asm5 --secondary=no --cs) to generate PAF output. Scaffolds were assigned to CanFam3.1 chromosomes using PAFScaff v0.2.0 [(https://github.com/slimsuite/pafscaff)](https://github.com/slimsuite/pafscaff) based on Minimap2-aligned assembly scaffold coverage against the reference chromosomes. Scaffolds were assigned to the chromosome with highest total coverage. Scaffolds failing to map onto a chromosome were rated as "Unplaced".

Formatted: Line spacing: Double

Final s~~S~~caffold c~~C~~lassification

Subreads were re-mapped on to the renamed and reoriented scaffolds for a final round of PurgeHaplotigs analysis to classify scaffolds that may have escaped filtering or have unusual read depth profiles. Scaffolds were placed into one of five categories:

1. DIPLOID (core) scaffolds have <50% match to another Scaffold and the dominant PurgeHaplotigs coverage bin is Diploid depth
2. REPEAT scaffolds have >50% match to another Scaffold and the dominant PurgeHaplotigs coverage bin is Diploid depth
3. COLLAPSED_REPEAT scaffolds have high coverage PurgeHaplotigs bin dominant

4. HAPLOID regions have >=50% match to another Scaffold and the dominant PurgeHaplotigs coverage bin is Haploid depth, but filtering criteria were not met
5. LOWQUALITY scaffolds have >=50% match to another Scaffold and the dominant PurgeHaplotigs coverage bin is low coverage depth, but filtering criteria were not met

Finally, twenty REPEAT scaffolds corresponding to a PacBio control sequence were removed from the assembly, leaving the final 409 nuclear scaffolds plus mitochondrion. Seventeen scaffolds had small regions masked or trimmed by the NCBI Contamination screen, corresponding to a 3.4kb chunk of Escherichia coli.

Formatted: Normal, Left, Don't keep with next

Formatted: Font color: Gray-80%

Formatted: Font color: Gray-80%, English (Australia)

Gene prediction including Annotation annotation of repetitive elements

The genome was annotated using the homology-based gene prediction program GeMoMa [27] (v1.6.2beta) and nine reference organisms. The nine species used for the homology-based gene prediction analyses were *Canis lupus familiaris* (CanFam3.1; GCF_000002285.3), *Vulpes vulpes* (VulVul2.2; GCF_003160815.1), *Felis catus* (Felis_catus_9.0; GCF_000181335.3), *Sus scrof* (Sscrofa11.1; GCF_000003025.6), *Bos taurus* (ARS-UCD1.2; GCF_002263795.1), *Ailuropoda melanoleuca* (ASM200744v1; GCF_000004335.2), *Ursus maritimus* (UrsMar_1.0; GCA_000687225.1), *Mus musculus* (GRCm38.p6; GCF_000001635.26), and *Homo sapiens* (GRCh38.p13; GCA_000001405.39), These reference organisms comprise *Canis lupus familiaris* (boxer dog), *Vulpes vulpes* (red fox), *Felis catus* (domestic Abyssinian cat), *Sus scrofa* (pig), *Bos taurus* (Hereford cattle), *Ailuropoda melanoleuca* (giant panda), *Mus musculus* (house mouse), *Homo sapiens* (human), and *Ursus maritimus* (polar bear), which were downloaded from NCBI.

Formatted: Justified

For each reference organism, coding exons of full-length transcript were extracted and translated to peptides using the GeMoMa module Extractor. These peptides were searched in the GSD genome using mmseqs2 [65] (v-5877873cbcd50a6d954607fc2df1210f8c2c3a4b). Based on the results of mmseqs2 and Extractor, transcripts were predicted for GSD from each reference organism independently. These nine gene annotation sets were then combined into a final gene annotation using the GeMoMa module GAF.

rRNA gGenes for rRNA were predicted with Barrnap v0.9 ((https://github.com/tseemann/barrnap) in the eukaryotic mode, HMMer v3.2.1 [66] and BEDTools v2.27.1 (https://github.com/arq5x/bedtools2).

Acknowledgements

Comments from two reviewers improved the manuscript. We would like to thank Helaya-Henderson Smith for providing frequent access to *Nala*. Staff at the Vineyard Veterinary Hospital provided constant encouragement. Martin SmithJames Ferguson was instrumental in facilitating the ONT data collection. A whole blood sample for Hi-C library preparation was provided by Susan Garrison LVT, BT, Sample Collection Coordinator, Cornell Veterinary Biobank. SMRT sequencing was conducted at the Ramaciotti Center for Comparative Genomics at University of New South Wales and at the Arizona Genomic Institute, University of Arizona. The ONT, 10X Chromium and Bionano genomics data were collected at the Garvan Institute and the Hi-C data at Baylor College of Medicine.

Commented [w1]: Martin Smith is now included as an author.

Availability of supporting data and materials

The complete genome build is available at NCBI (GenBank accession number GCA_008641055.1; https://www.ncbi.nlm.nih.gov/assembly/GCA_008641055.1). DNA Methylation data GEO entry Series is GSE136348 with reviewer token: mhyhmwwuzxonjcj*. PAFScaff is registered at SciCrunch.org (SCR_017976) and bio.tools (https://bio.tools/PAFScaff_Pairwise_mApping_Format_reference-based_scaffold_anchoring_and_super-scaffolding.).*

Additional Files

Supplementary File 1: Read depth analysis of *Amy2B* region

Supplementary File 2: Bionano *AMY2B* methods

Supplementary File 3: 10X chromium workflow details

Supplementary Figure 1. Schematic overview of project workflow

Supplementary Figure 2: BUSCO improvements in assembly quality at each analysis step

Supplementary Figure 3: KAT k-mer analysis of Nala assembly

Supplementary Figure 4: Bionano consensus maps aligned to GSD contig NALACHR6.01

Supplementary Figure 5: Bionano consensus maps aligned to CanFam3 Chr6

Supplementary Figure 6: Hip X~~x~~-ray of the German Shepherd Dog *Nala*

Supplementary Figure 7: DNA methylation profiling of German Shepherd Dog *Nala's* whole blood

~~Supplemental~~Supplementary Table 1. Summary assembly scaffold and BUSCO stati~~si~~stics for different *Nala* assembly stages, CanFam 3.1, and compiled best ratings.

~~Supplemental~~Supplementary Table 2. GSD SNVs and small indels summary by chromosome

~~Supplemental~~Supplementary Table 3. GSD copy number and structural variants (>100bp-) summary by chromosome.

Abbreviations

BLAST: Basic Local Alignment Search Tool**; BMG:** Bionano Genomics**; bp:** base pairs**; BUSCO:** Benchmarking Universal Single-Copy Orthologs**; CHD:** Canine hip dysplasia**;** ~~**d.p.:** decimal point;~~ **CNV:** Copy number variant**; d.p.:** decimal point; **gDNA:** genomic DNA**; GSD:** German Shepherd Dog**; HMM:** hidden Markov model**; HME:** High Molecular Weight**; ONT:** Oxford Nanopore Technologies**; ORF:** open reading frame**; PacBio:** Pacific Biosciences**; PCR:** polymerase chain reaction**; qPCR:** quantitative polymerase chain reaction**; RNA-seq:** RNA sequencing**; s.f.:** significant figure**; SMRT:** single-molecule real time; **SNV:** single-nucleotide variant; **SV**; s~~S~~tructural v~~V~~ariant

Ethics approval and consent to participate

All experimentation was performed under the approval of the University of New South Wales Ethics Committee (ACEC ID: 18/18B).

Competing interests

The authors declare that they have no competing interests.

Funding

This work was supported by the Australian Health Foundation award and to the Hip2Fit Crowdfunding initiative to J.W.O.B. and R.Z. Matching funds were provided by the University of New South Wales/ School of Biotechnology and Biomolecular Sciences Genomics Initiative. V.M.H. funded the Bionano data collection and the DNA Zoo initiative of E.A.L. funded the Hi-C data collection and analyses. M.A.F. is funded by NHMRC APP5121190. E.L.A. was supported by an NSF Physics Frontiers Center Award (PHY1427654), the Welch

Formatted: Font: 12 pt

Formatted: Font: 12 pt

Formatted: Font: 12 pt

Formatted: Font: 12 pt

Formatted: Font: 12 pt

Foundation (Q-1866), a USDA Agriculture and Food Research Initiative Grant (2017-05741), an NIH 4D Nucleome Grant (U01HL130010), and an NIH Encyclopedia of DNA Elements Mapping Center Award (UM1HG009375). The Ramaciotti Centre for Genomics acknowledge infrastructure funding from the Australian Research Council (LE150100031), the Australian Government NCRIS scheme administered by Bioplatforms Australia, and the New South Wales Government RAAP scheme.

Author contributions

J.W.O.B. coordinated the project. M.A.F., B.D.R., T.P.L.S and J.W.O.B. designed the study. J.W.O.B funded the project. R.A.Z. provided the GSD samples. R.L., and D.T., performed genomic DNA extractions. K.B. and M.A.S. performed the ONT sequencing and R.L. the Bionano optical mapping. B.D.R. performed the initial assembly and polishing, A.E.R. performed the chromium scaffolding, E.K.F.C and V.M.H. performed the Bionano super-scaffolding. O.D., A.O. and Z.C. performed the Hi-C experiment, and O.D. and E.L.A. conducted the Hi-C analyses. K.S. and O.B. conducted the DNA methylation analyses. M.A.F. and R.E performed all analyses of genome completeness. R.E. performed the final polishing, final assembly clean up, and KAT analysis. J.K. performed the genome annotation. R.E. performed the rRNA annotation~~J.K. performed the genome annotation~~. R.E., E.K.F.C. and B.D.R. performed the *AMY2B* analyses. M.F., B.D.R., O.D., R.E., A.E.M., E.K.F.C., O.B. and J.W.O.B. wrote the manuscript. All authors edited and approved the final manuscript.

References

1. Frantz LA, Mullin VE, Pionnier-Capitan M, Lebrasseur O, Ollivier M, Perri A, et al. Genomic and archaeological evidence suggest a dual origin of domestic dogs. Science. 2016;352 6290:1228-31. doi:10.1126/science.aaf3161.

2. Freedman AH, Gronau I, Schweizer RM, Ortega-Del Vecchyo D, Han E, Silva PM, et al. Genome sequencing highlights the dynamic early history of dogs. PLoS Genet. 2014;10 1:e1004016. doi:10.1371/journal.pgen.1004016.
3. Savolainen P, Zhang YP, Luo J, Lundeberg J and Leitner T. Genetic evidence for an East Asian origin of domestic dogs. Science. 2002;298 5598:1610-3. doi:10.1126/science.1073906.
4. Thalmann O, Shapiro B, Cui P, Schuenemann VJ, Sawyer SK, Greenfield DL, et al. Complete mitochondrial genomes of ancient canids suggest a European origin of domestic dogs. Science. 2013;342 6160:871-4. doi:10.1126/science.1243650.
5. Vonholdt BM, Pollinger JP, Lohmueller KE, Han E, Parker HG, Quignon P, et al. Genome-wide SNP and haplotype analyses reveal a rich history underlying dog domestication. Nature. 2010;464 7290:898-902. doi:10.1038/nature08837.
6. Parker HG, Dreger DL, Rimbault M, Davis BW, Mullen AB, Carpintero-Ramirez G, et al. Genomic Analyses Reveal the Influence of Geographic Origin, Migration, and Hybridization on Modern Dog Breed Development. Cell Rep. 2017;19 4:697-708. doi:10.1016/j.celrep.2017.03.079.
7. Talenti A, Dreger DL, Frattini S, Polli M, Marelli S, Harris AC, et al. Studies of modern Italian dog populations reveal multiple patterns for domestic breed evolution. Ecol Evol. 2018;8 5:2911-25. doi:10.1002/ece3.3842.
8. Willis MB. The German Shepherd Dog: Its history, development and genetics. New York: Arco Publishing Company; 1977.
9. Samms S. German Shepherd Dog: a comprehensive guide to owning and caring for your dog. London: Kennel Club Books; 2003.
10. Benninger MI, Seiler GS, Robinson LE, Ferguson SJ, Bonel HM, Busato AR, et al. Three-dimensional motion pattern of the caudal lumbar and lumbosacral portions of the vertebral column of dogs. Am J Vet Res. 2004;65 5:544-51.
11. Shaffer LG, Ramirez CJ, Phelps P, Aviram M, Walczak M, Bar-Gal GK, et al. An International Genetic Survey of Breed-Specific Diseases in Working Dogs from the United States, Israel, and Poland. Cytogenet Genome Res. 2017;153 4:198-204. doi:10.1159/000486774.
12. Boge GS, Moldal ER, Dimopoulou M, Skjerve E and Bergstrom A. Breed susceptibility for common surgically treated orthopaedic diseases in 12 dog breeds. Acta Vet Scand. 2019;61 1:19. doi:10.1186/s13028-019-0454-4.
13. Peiravan A, Bertolini F, Rothschild MF, Simpson KW, Jergens AE, Allenspach K, et al. Genome-wide association studies of inflammatory bowel disease in German shepherd dogs. PLoS One. 2018;13 7:e0200685. doi:10.1371/journal.pone.0200685.
14. Soo M, Lopez-Villalobos N and Worth AJ. Heritabilities and genetic trends for elbow score as recorded by the New Zealand Veterinary Association Elbow Dysplasia Scheme (1992-2013) in four breeds of dog. N Z Vet J. 2018;66 3:154-61. doi:10.1080/00480169.2018.1440652.
15. Wah lJM, Herbst SM, Clark LA, Tsai KL and Murphy KE. A review of hereditary diseases of the German shepherd dog. J Vet Behav. 2008; 3:255-65.
16. Christopherson PW, Bacek LM, King KB and Boudreaux MK. Two novel missense mutations associated with hemophilia A in a family of Boxers, and a German Shepherd dog. Vet Clin Pathol. 2014;43 3:312-6. doi:10.1111/vcp.12172.
17. Shariflou MR, James JW, Nicholas FW and Wade CM. A genealogical survey of Australian registered dog breeds. Vet J. 2011;189 2:203-10. doi:10.1016/j.tvjl.2011.06.020.

18. Koren S, Walenz BP, Berlin K, Miller JR, Bergman NH and Phillippy AM. Canu: scalable and accurate long-read assembly via adaptive k-mer weighting and repeat separation. Genome Res. 2017;27 5:722-36. doi:10.1101/gr.215087.116.
19. GenomicConsensus P. https://github.com/PacificBiosciences/GenomicConsensus.
20. Walker BJ, Abeel T, Shea T, Priest M, Abouelliel A, Sakthikumar S, et al. Pilon: an integrated tool for comprehensive microbial variant detection and genome assembly improvement. PLoS One. 2014;9 11:e112963. doi:10.1371/journal.pone.0112963.
21. Lindblad-Toh K, Wade CM, Mikkelsen TS, Karlsson EK, Jaffe DB, Kamal M, et al. Genome sequence, comparative analysis and haplotype structure of the domestic dog. Nature. 2005;438 7069:803-19. doi:10.1038/nature04338.
22. Simao FA, Waterhouse RM, Ioannidis P, Kriventseva EV and Zdobnov EM. BUSCO: assessing genome assembly and annotation completeness with single-copy orthologs. Bioinformatics. 2015;31 19:3210-2. doi:10.1093/bioinformatics/btv351.
23. Altschul SF, Gish W, Miller W, Myers EW and Lipman DJ. Basic local alignment search tool. J Mol Biol. 1990;215 3:403-10. doi:10.1016/S0022-2836(05)80360-2.
24. Finn RD, Clements J and Eddy SR. HMMER web server: interactive sequence similarity searching. Nucleic Acids Res. 2011;39 Web Server issue:W29-37. doi:10.1093/nar/gkr367.
25. Stanke M and Morgenstern B. AUGUSTUS: a web server for gene prediction in eukaryotes that allows user-defined constraints. Nucleic Acids Res. 2005;33 Web Server issue:W465-7. doi:10.1093/nar/gki458.
26. Mapleson D, Garcia Accinelli G, Kettleborough G, Wright J and Clavijo BJ. KAT: a K-mer analysis toolkit to quality control NGS datasets and genome assemblies. Bioinformatics. 2017;33 4:574-6. doi:10.1093/bioinformatics/btw663.
27. Keilwagen J, Hartung F and Grau J. GeMoMa: Homology-Based Gene Prediction Utilizing Intron Position Conservation and RNA-seq Data. Methods Mol Biol. 2019;1962:161-77. doi:10.1007/978-1-4939-9173-0_9.
28. Marcais G, Delcher AL, Phillippy AM, Coston R, Salzberg SL and Zimin A. MUMmer4: A fast and versatile genome alignment system. PLoS Comput Biol. 2018;14 1:e1005944. doi:10.1371/journal.pcbi.1005944.
29. Ollivier M, Tresset A, Bastian F, Lagoutte L, Axelsson E, Arendt ML, et al. Amy2B copy number variation reveals starch diet adaptations in ancient European dogs. R Soc Open Sci. 2016;3 11:160449. doi:10.1098/rsos.160449.
30. Quignon P, Giraud M, Rimbault M, Lavigne P, Tacher S, Morin E, et al. The dog and rat olfactory receptor repertoires. Genome Biol. 2005;6 10:R83. doi:10.1186/gb-2005-6-10-r83.
31. Balmer P, Bauer A, Pujar S, McGarvey KM, Welle M, Galichet A, et al. A curated catalog of canine and equine keratin genes. PLoS One. 2017;12 8:e0180359. doi:10.1371/journal.pone.0180359.
32. Olender T, Fuchs T, Linhart C, Shamir R, Adams M, Kalush F, et al. The canine olfactory subgenome. Genomics. 2004;83 3:361-72. doi:10.1016/j.ygeno.2003.08.009.
33. Bateson P. Independent inquiry into dog breeding. Cambridge: University of Cambridge; 2010.
34. Rooney N and Sargan D. Pedigree dog breeding in the UK: a major welfare concern? . Horsham, West Sussex: RSPCA; 2008.
35. Asher L, Diesel G, Summers JF, McGreevy PD and Collins LM. Inherited defects in pedigree dogs. Part 1: disorders related to breed standards. Vet J. 2009;182 3:402-11. doi:10.1016/j.tvjl.2009.08.033.

36. Petazzoni M, Piras A, Jaeger GH and Marioni C. Correction of rotational deformity of the pes with external skeletal fixation in four dogs. Vet Surg. 2009;38 4:506-14. doi:10.1111/j.1532-950X.2009.00519.x.
37. Hamann H, Kirchhoff T and Distl O. Bayesian analysis of heritability of canine hip dysplasia in German Shepherd Dogs. J Anim Breed Genet. 2003;120:258-68.
38. Sanchez-Molano E, Woolliams JA, Pong-Wong R, Clements DN, Blott SC and Wiener P. Quantitative trait loci mapping for canine hip dysplasia and its related traits in UK Labrador Retrievers. BMC Genomics. 2014;15:833. doi:10.1186/1471-2164-15-833.
39. Zhu L, Zhang Z, Friedenberg S, Jung SW, Phavaphutanon J, Vernier-Singer M, et al. The long (and winding) road to gene discovery for canine hip dysplasia. Vet J. 2009;181 2:97-110. doi:10.1016/j.tvjl.2009.02.008.
40. Mikkola LI, Holopainen S, Lappalainen AK, Pessa-Morikawa T, Augustine TJP, Arumilli M, et al. Novel protective and risk loci in hip dysplasia in German Shepherds. PLoS Genet. 2019;15 7:e1008197. doi:10.1371/journal.pgen.1008197.
41. Saetre P, Strandberg E, Sundgren PE, Pettersson U, Jazin E and Bergstrom TF. The genetic contribution to canine personality. Genes, Brain and Behavior. 2006;5:240-8.
42. Robin S, Tacher S, Rimbault M, Vaysse A, Dreano S, Andre C, et al. Genetic diversity of canine olfactory receptors. BMC Genomics. 2009;10:21. doi:10.1186/1471-2164-10-21.
43. Yang M, Geng GJ, Zhang W, Cui L, Zhang HX and Zheng JL. SNP genotypes of olfactory receptor genes associated with olfactory ability in German Shepherd dogs. Anim Genet. 2016;47 2:240-4. doi:10.1111/age.12389.
44. Bigi D, Marelli SP, Randi E and Polli M. Genetic characterization of four native Italian shepherd dog breeds and analysis of their relationship to cosmopolitan dog breeds using microsatellite markers. Animal. 2015;9 12:1921-8. doi:10.1017/S1751731115001561.
45. Urich MA, Nery JR, Lister R, Schmitz RJ and Ecker JR. MethylC-seq library preparation for base-resolution whole-genome bisulfite sequencing. Nat Protoc. 2015;10 3:475-83. doi:10.1038/nprot.2014.114.
46. Meissner A, Mikkelsen TS, Gu H, Wernig M, Hanna J, Sivachenko A, et al. Genome-scale DNA methylation maps of pluripotent and differentiated cells. Nature. 2008;454 7205:766-70. doi:10.1038/nature07107.
47. Bogdanovic O, Smits AH, de la Calle Mustienes E, Tena JJ, Ford E, Williams R, et al. Active DNA demethylation at enhancers during the vertebrate phylotypic period. Nat Genet. 2016;48 4:417-26. doi:10.1038/ng.3522.
48. Burger L, Gaidatzis D, Schubeler D and Stadler MB. Identification of active regulatory regions from DNA methylation data. Nucleic Acids Res. 2013;41 16:e155. doi:10.1093/nar/gkt599.
49. Stadler MB, Murr R, Burger L, Ivanek R, Lienert F, Scholer A, et al. DNA-binding factors shape the mouse methylome at distal regulatory regions. Nature. 2011;480 7378:490-5. doi:10.1038/nature10716.
50. Mo A, Mukamel EA, Davis FP, Luo C, Henry GL, Picard S, et al. Epigenomic Signatures of Neuronal Diversity in the Mammalian Brain. Neuron. 2015;86 6:1369-84. doi:10.1016/j.neuron.2015.05.018.
51. Rao SS, Huntley MH, Durand NC, Stamenova EK, Bochkov ID, Robinson JT, et al. A 3D map of the human genome at kilobase resolution reveals principles of chromatin looping. Cell. 2014;159 7:1665-80. doi:10.1016/j.cell.2014.11.021.

52. Yeo S, Coombe L, Warren RL, Chu J and Birol I. ARCS: scaffolding genome drafts with linked reads. Bioinformatics. 2018;34 5:725-31. doi:10.1093/bioinformatics/btx675.
53. Li H. Minimap2: pairwise alignment for nucleotide sequences. Bioinformatics. 2018;34 18:3094-100. doi:10.1093/bioinformatics/bty191.
54. Vaser R, Sovic I, Nagarajan N and Sikic M. Fast and accurate de novo genome assembly from long uncorrected reads. Genome Res. 2017;27 5:737-46. doi:10.1101/gr.214270.116.
55. Hastie AR, Dong L, Smith A, Finklestein J, Lam ET, Huo N, et al. Rapid genome mapping in nanochannel arrays for highly complete and accurate de novo sequence assembly of the complex Aegilops tauschii genome. PLoS One. 2013;8 2:e55864. doi:10.1371/journal.pone.0055864.
56. Lam ET, Hastie A, Lin C, Ehrlich D, Das SK, Austin MD, et al. Genome mapping on nanochannel arrays for structural variation analysis and sequence assembly. Nat Biotechnol. 2012;30 8:771-6. doi:10.1038/nbt.2303.
57. Durand NC, Robinson JT, Shamim MS, Machol I, Mesirov JP, Lander ES, et al. Juicebox Provides a Visualization System for Hi-C Contact Maps with Unlimited Zoom. Cell Syst. 2016;3 1:99-101. doi:10.1016/j.cels.2015.07.012.
58. Dudchenko O, Batra SS, Omer AD, Nyquist SK, Hoeger M, Durand NC, et al. *De novo* assembly of the *Aedes aegypti* genome using Hi-C yields chromosome-length scaffolds. Science. 2017;356 6333:92-5. doi:10.1126/science.aal3327.
59. Dudchenko O, Shamim MS, Batra SS, Durand NC, Musial NT, Mostofa R, et al. The Juicebox Assembly Tools module facilitates de novo assembly of mammalian genomes with chromosome-length scaffolds for under $1000. bioRxiv. 2018:254797. doi:10.1101/254797.
60. Robinson JT, Turner D, Durand NC, Thorvaldsdottir H, Mesirov JP and Aiden EL. Juicebox.js Provides a Cloud-Based Visualization System for Hi-C Data. Cell Syst. 2018;6 2:256-8 e1. doi:10.1016/j.cels.2018.01.001.
61. English AC, Richards S, Han Y, Wang M, Vee V, Qu J, et al. Mind the gap: upgrading genomes with Pacific Biosciences RS long-read sequencing technology. PLoS One. 2012;7 11:e47768. doi:10.1371/journal.pone.0047768.
62. Roach MJ, Schmidt SA and Borneman AR. Purge Haplotigs: allelic contig reassignment for third-gen diploid genome assemblies. BMC Bioinformatics. 2018;19 1:460. doi:10.1186/s12859-018-2485-7.
63. Quinlan AR and Hall IM. BEDTools: a flexible suite of utilities for comparing genomic features. Bioinformatics. 2010;26 6:841-2. doi:10.1093/bioinformatics/btq033.
64. Li H, Handsaker B, Wysoker A, Fennell T, Ruan J, Homer N, et al. The Sequence Alignment/Map format and SAMtools. Bioinformatics. 2009;25 16:2078-9. doi:10.1093/bioinformatics/btp352.
65. Steinegger M and Soding J. MMseqs2 enables sensitive protein sequence searching for the analysis of massive data sets. Nat Biotechnol. 2017;35 11:1026-8. doi:10.1038/nbt.3988.
66. Wheeler TJ and Eddy SR. nhmmer: DNA homology search with profile HMMs. Bioinformatics. 2013;29 19:2487-9. doi:10.1093/bioinformatics/btt403.

Figure 1

Click here to access/download;Figure;Fig1.jpg

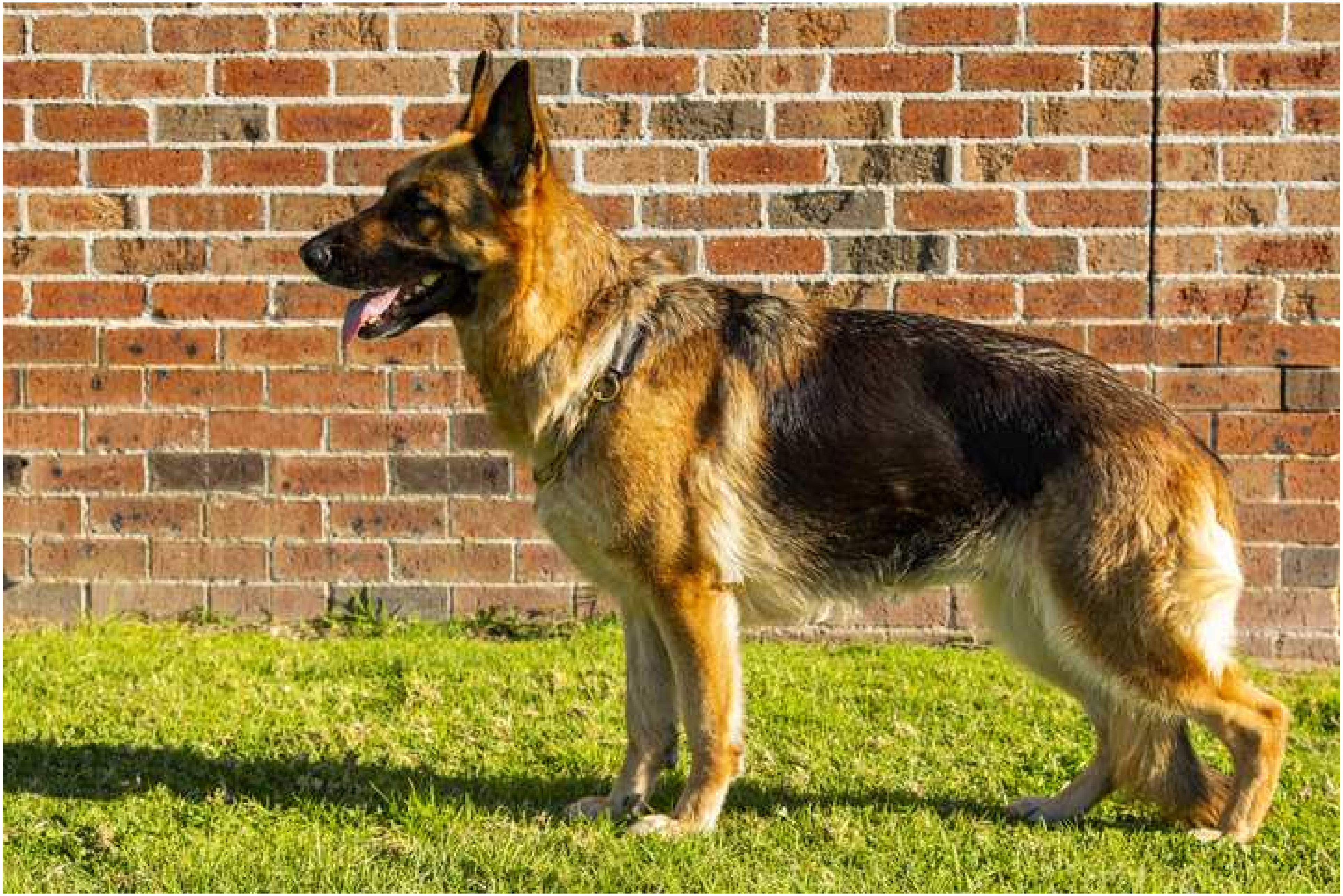

Figure 2

Click here to access/download;Figure;Fig2.pdf

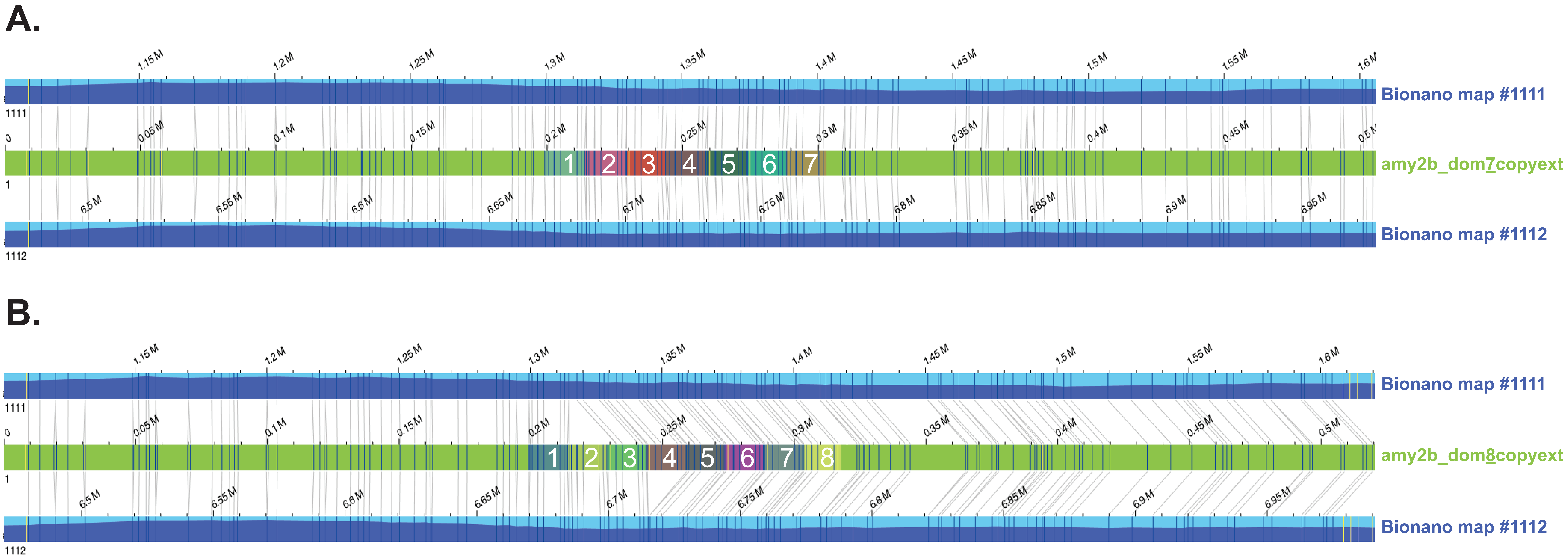

Figure 3

Click here to access/download;Figure;Fig3.tiff

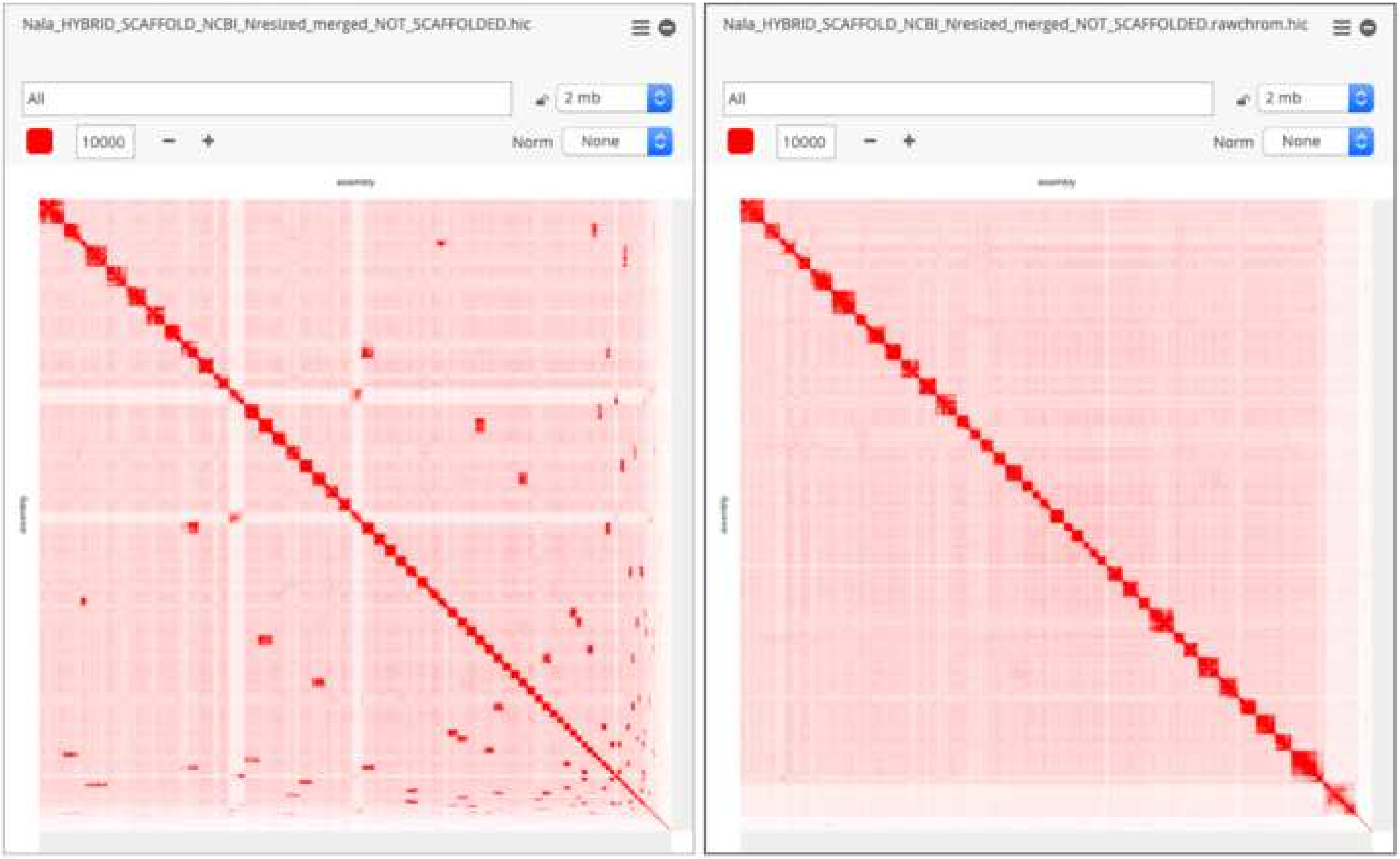

Supplementary Table 1

Click here to access/download
Supplementary Material
Supp_Table1.xlsx

Supplementary Table 2

Click here to access/download
Supplementary Material
Supp_Table2.xlsx

Supplementary Table 3

Click here to access/download
Supplementary Material
Supp_Table3.xlsx

Supplementary File 1

Click here to access/download
Supplementary Material
Supp_File1.docx

Supplementary File 2

Click here to access/download
Supplementary Material
Supp_File2.docx

Supplementary File 3

Click here to access/download
Supplementary Material
Supp_File3.docx

Supplementary Figure 1

Click here to access/download
Supplementary Material
Supp_Fig1.docx

Supplementary Figure 2

Click here to access/download
Supplementary Material
Supp_Fig2.docx

Supplementary Figure 3

Click here to access/download
Supplementary Material
Supp_Fig3.docx

Supplementary Figure 4

Click here to access/download
Supplementary Material
Supp_Fig4.docx

Supplementary Figure 5

Click here to access/download
Supplementary Material
Supp_Fig5.docx

Supplementary Figure 6

Click here to access/download
Supplementary Material
Supp_Fig6.docx

Supplementary Figure 7

Click here to access/download
Supplementary Material
Supp_Fig7.docx
