# Supplementary material for: Canfam_GSD: De novo chromosome-length genome assembly of the German Shepherd Dog (Canis lupus familiaris) using a combination of long reads, optical mapping, and Hi-C
Source: Gigascience. 2020 Apr 1;9(4):giaa027. doi: 10.1093/gigascience/giaa027 (PMC7111595; doi:10.1093/gigascience/giaa027)
Supplement: giaa027_Supplemental_Files [file giaa027_supplemental_files.zip › Supp_File1.docx]

**Supplementary File 1: AMY2B Dominant haplotype copy number depth analysis**

**Reassembly of AMY2B region**

A region of Nala Chromosome 6 (NALACHR6.01, 47330001-47406000) spanning the AMY2B repeats and including an apparent upstream missassembly was selected for reassembly. PacBio and ONT reads mapping to the AMY2B region and covering at least 3kb of the reference were extracted using samtools v1.9 (-m 3000) [1]. In total, 484 PacBio subreads (6.51 Mb) and 210 ONT reads (3.97 Mb) were extracted. These reads were then reassembled using Flye v2.4.2 and a predicted genome size of 132 kb. Flye generated 3 contigs representing (1) 86.5 kb upstream of the AMY2B repeat, including a 10.9 kb insertion versus NALACHR6.01, (2) a 14.8 kb AMY2B repeat unit, and (3) a 38.0 kb downstream region.

**Construction of variable repeat copy number regions**

A modified version of the AMY2B region was constructed with a variable number of AMY2B repeat sequences in attempt to predict the true copy number using read depths. To minimise the chance of biases due to inconsistent read mapping, as identical repeat unit was used in this analysis. Based on read depth of long reads mapped onto the original NALACHR6.01 region, repeat 3 (R0) was identified as the dominant AMY2B repeat sequence. An expanded region of NALACHR6.01 (47150001-47600000) was extracted and modified to include the 10.9 kb Flye insertion. The AMY2B repeat region was then replaced with 7 or 8 tandem repeats of AMY2B repeat unit 3. The final re-assemblies consisted of 7 or 8 AMY2B repeats and approx. 200kb flanking each side (Supplementary File 1: Figure 1A).

**Calculation of expected read depth**

PacBio subreads were mapped onto each re-assembly individually, using minimap v2.16 (no secondary alignments) [2] and filtered with samtools v1.9 [1] to retain only those alignments with min. 3kb reference coverage. bbtools v38.51 pileup.sh was used to calculate the mean read depth across 500 bp bins (Supplementary File 1 – Figure 2). Single copy diploid (2n) read depths were derived using median read depth of the flanking regions. Some flanking regions were observed to be at possible haploid (1n) read depths, which would deflate median flanking depth (Supplementary File 1 - Figure 1). There was also a spike in coverage near the end of 3’ flanking region, which may correspond to a repetitive sequence. Analysis was therefore restricted to 35 kb each side of the repeat region, which appeared to have quite stable diploid (2n) read depth (median=29.0X). Median read depths across the repeat region were 32.8X for 7N and 27.9X for 8N (Supplementary File 1 – Figure 1 and Figure 2). Repeat copy number can also be calculated directly by dividing the total repeat read coverage by the predicted single copy read depth, which produced values of 7.83X (7N data) and 7.85X (8N data).

**(a)**


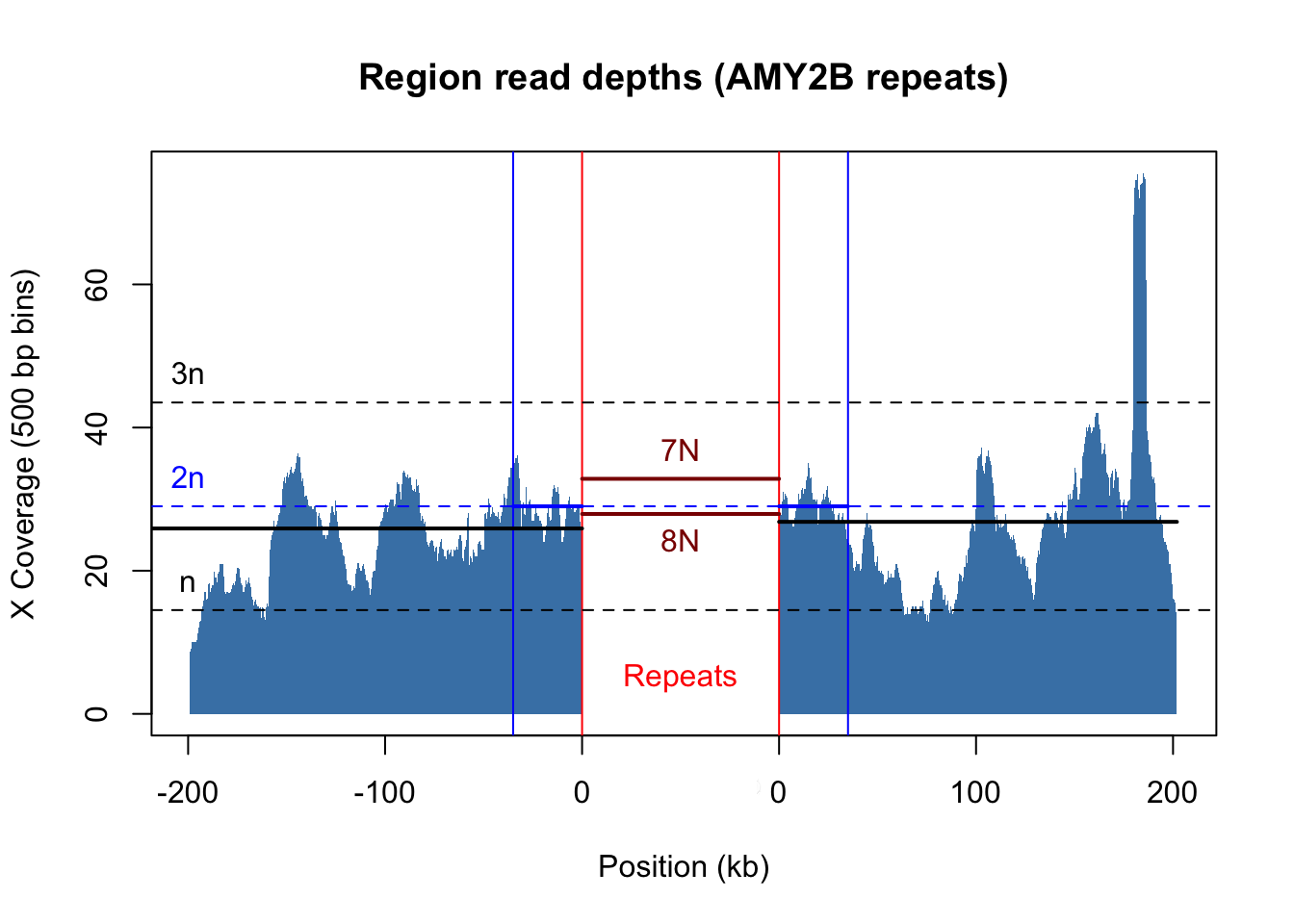


**(b)**


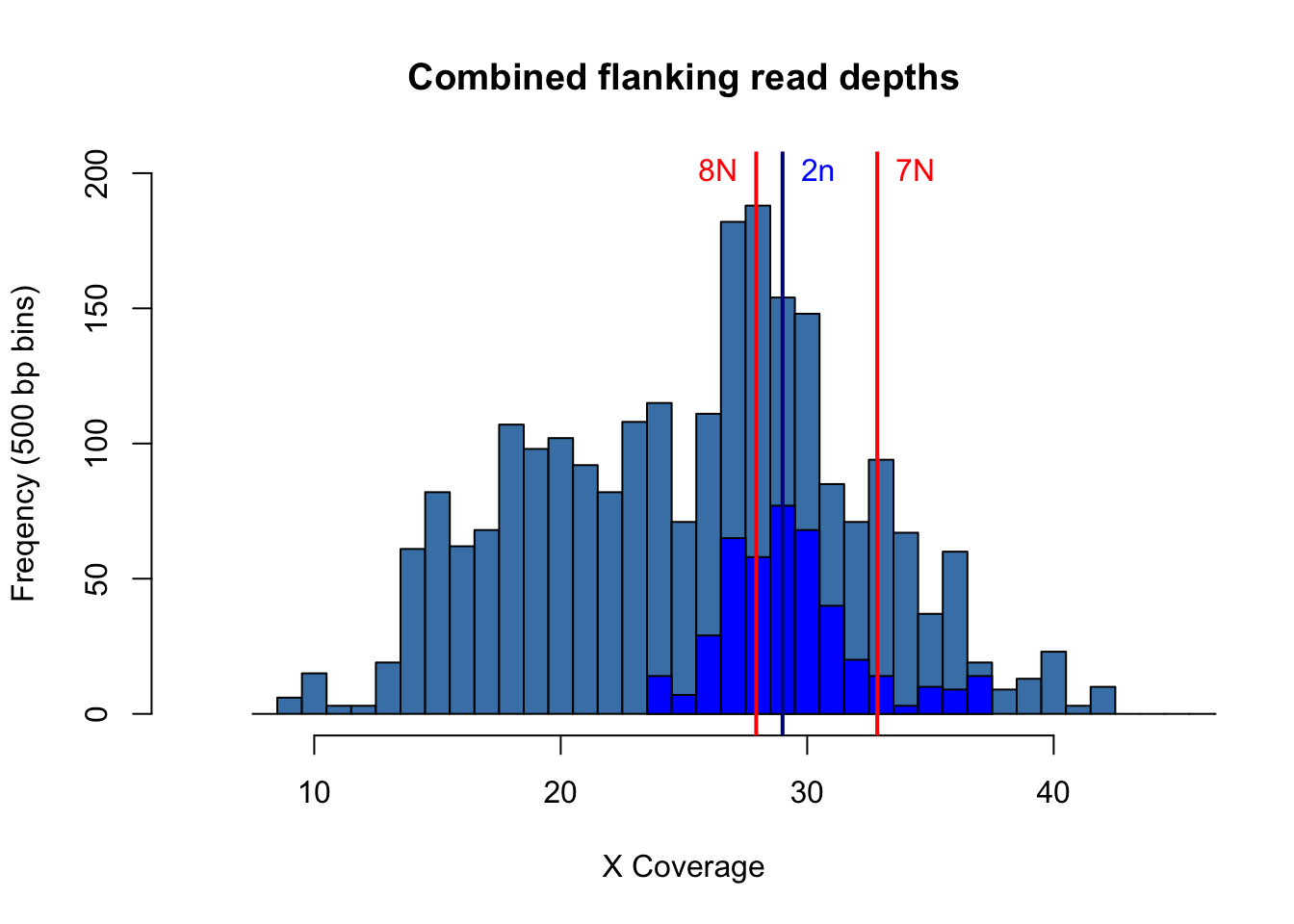


**Supplementary File 1 - Figure 1. PacBio read depth (mean coverage over 500 bp bins) across a reconstructed AMY2B repeat region +/- approx. 200kb flanks.** Read mapping was restricted to alignments covering at least 3kb of the reference sequence. **(a)** Depth plot of flanks. Vertical blue lines mark a reduced window of 35 kb flanking the repeats, which appears to be more consistently single copy. Solid horizontal lines mark median read depths for flanks (black), variable numbers of repeats (dark red, marked 7N and 8N), or restricted +/-35 kb flanks (blue). Dashed lines indicate n (haploid), 2n (diploid) and 3n (triploid) read depths as predicted from 35 kb flanks. **(b)** Histogram of flanking read depth for all flanks (light blue) and +/-35 kb flanks (blue). Solid red lines mark the predicted single copy (2n) read depth for different repeat copy numbers (7N, 8N). The solid blue line marks the +/-35 kb median depth.

**(a)**


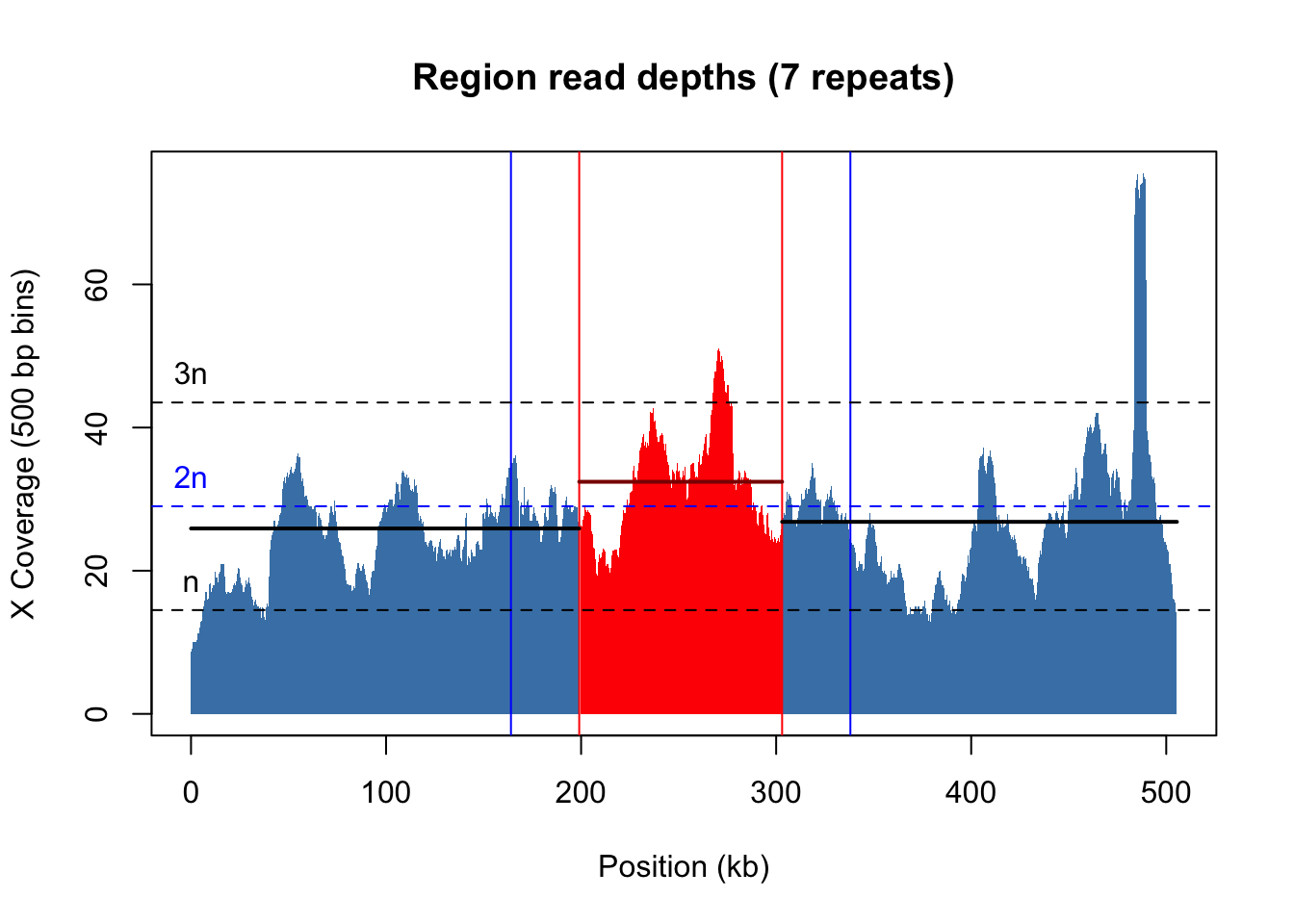


**(b)**


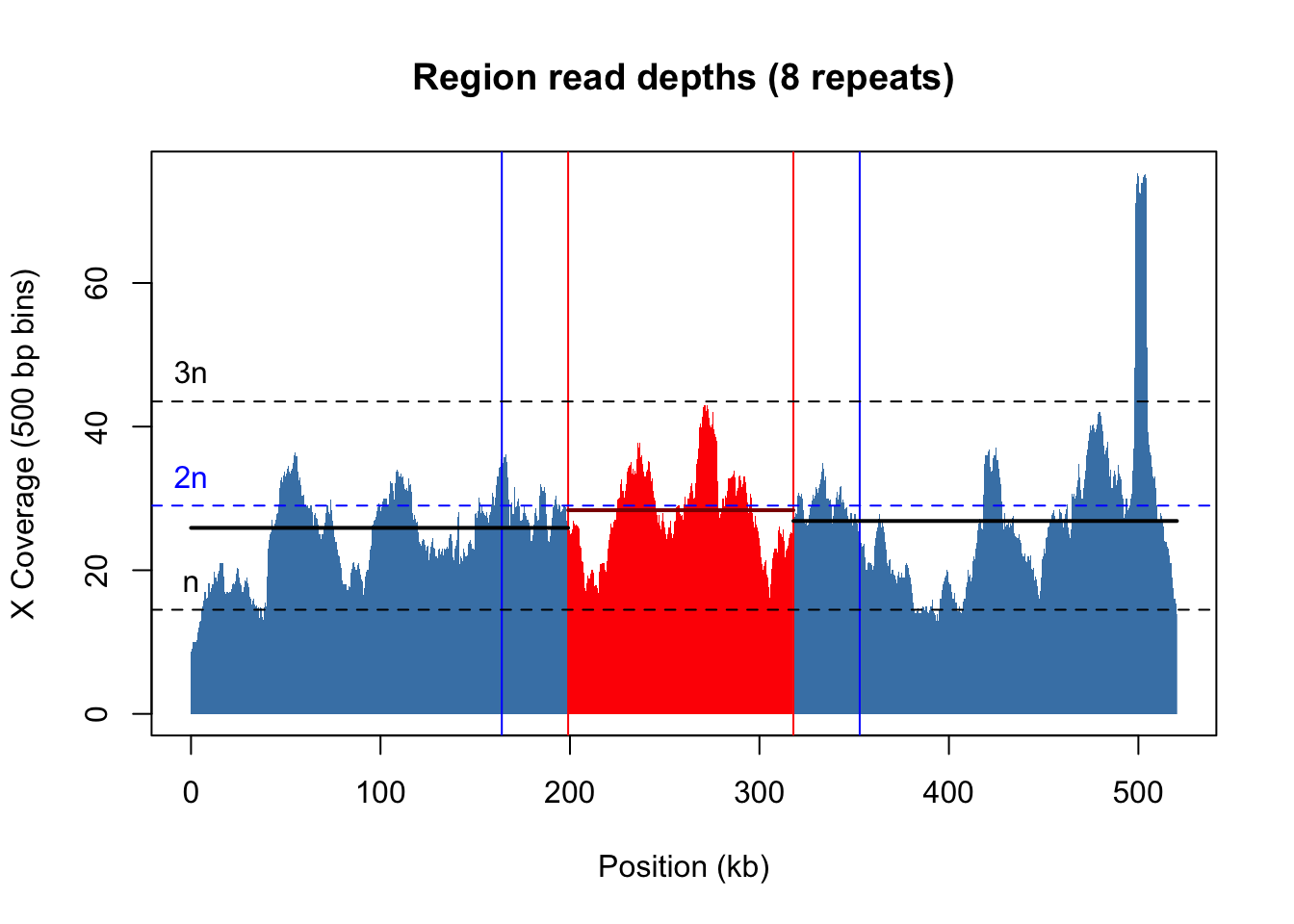


**Supplementary File 1 - Figure 2. PacBio read depth (mean coverage over 500 bp bins) across a reconstructed AMY2B repeat region +/- approx. 200kb flanks.** Read mapping was restricted to alignments covering at least 3kb of the reference sequence. Repeat regions are marked in red, with flanks in blue.Vertical blue lines mark a reduced window of 35 kb flanking the repeats, which appears to be more consistently single copy. Solid horizontal lines mark median read depths for flanks (black), repeat region (dark red), or restricted +/-35 kb flanks (blue). Dashed lines indicate n (haploid), 2n (diploid) and 3n (triploid) read depths as predicted from 35 kb flanks. **(a)** Seven AMY2B repeat copies (7N). **(b)** Eight AMY2B repeat copies (8N).

## AMY2B full assembly copy number depth analysis

**Using Single Copy BUSCO read depth to infer AMY2B copy number**

PacBio subreads and raw ONT reads were mapped onto the complete Nala assembly using minimap v2.16 [2] (no secondary alignments). Single Copy Complete (SCC) genes identified from the BUSCO v3.0.2b [3] (laurasiatheria_odb9) search were used to identify stretches of the genome that should be predominantly single copy. The region corresponding to each SCC BUSCO gene was extracted from the PacBio and ONT BAM files and read depth for each base calculated using samtools v1.9 [1] depth mode. This was repeated for the AMY2B region of NALACHR6.01 (47338205-47397584), which consisted of 4 copies of the 14.9 kb AMY2B-containing repeat.

Different proportions of hemizygous regions and collapsed repeat sequences in SCC BUSCO regions will affect mean and median read depths. Because the dominant signal is expected to correspond to single copy diploid read depth, the modal read depth for each SSC BUSCO region were calculated and the expected single copy read depth for each technology estimated as the median modal read depth (Supplementary File 1 - Figure 3). To estimate AMY2B copy number, the mean read depth for a single repeat copy (e.g. mean AMY2B region read depth multiplied by four) was calculated and divided by the median modal read depth. Similarly, the expected single copy read depth was calculated by dividing the repeat copy mean read depth by predicted repeat counts of seven (7N) or eight (8N) (Supplementary File 1 - Figure 3). The median modal SCC BUSCO read depths were 38X (PacBio) and 23X (ONT). Mean AMY2B repeat copy read depths were 285.1X (PacBio) and 182.6X (ONT). This gave AMY2B copy number predictions of 7.50 (PacBio) or 7.94 (ONT). These data predict one allele of 8 copies, and a second allele of 7 or 8 copies (Supplementary File 1 - Figure 3).

**(a)**
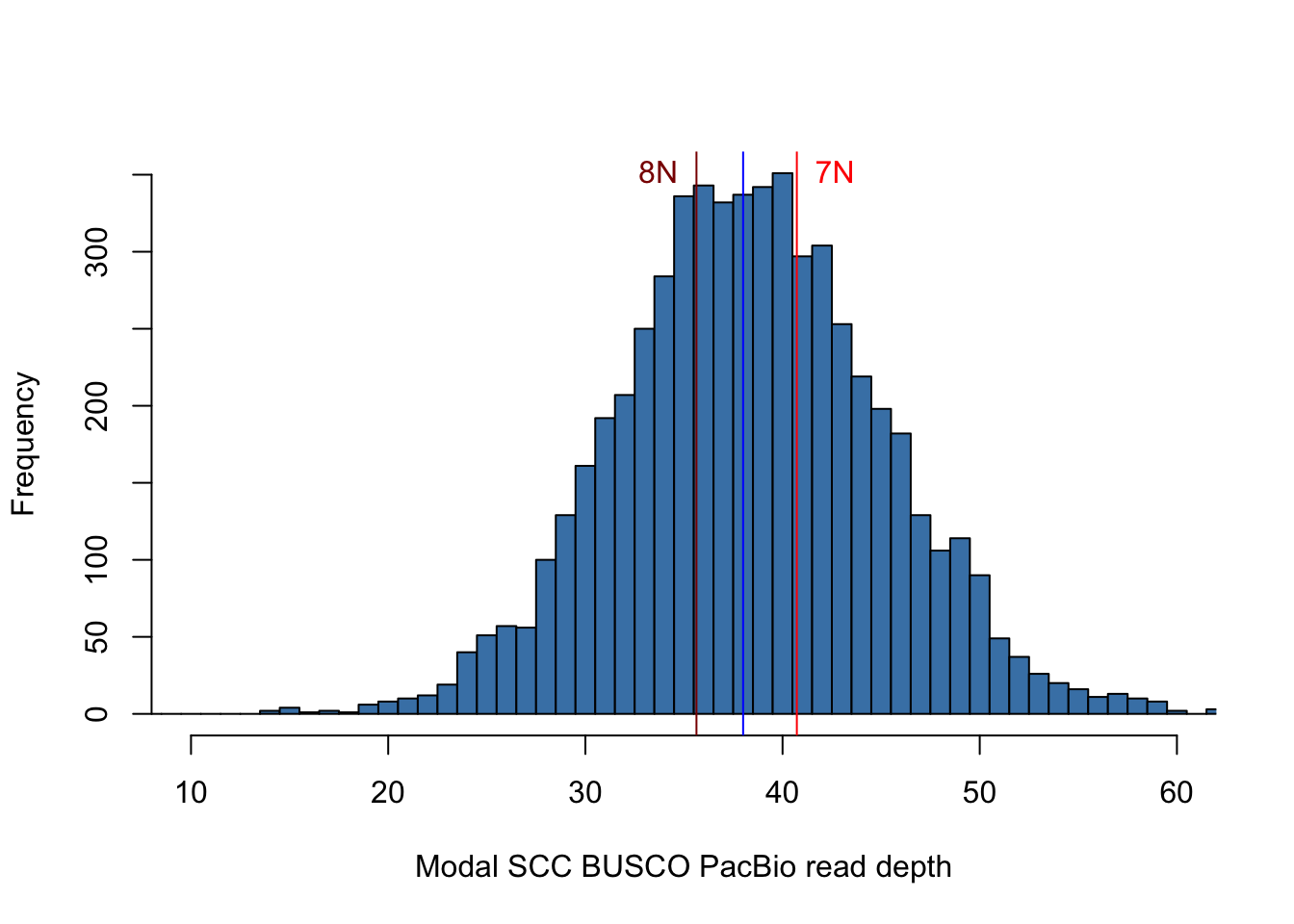


**(b)**


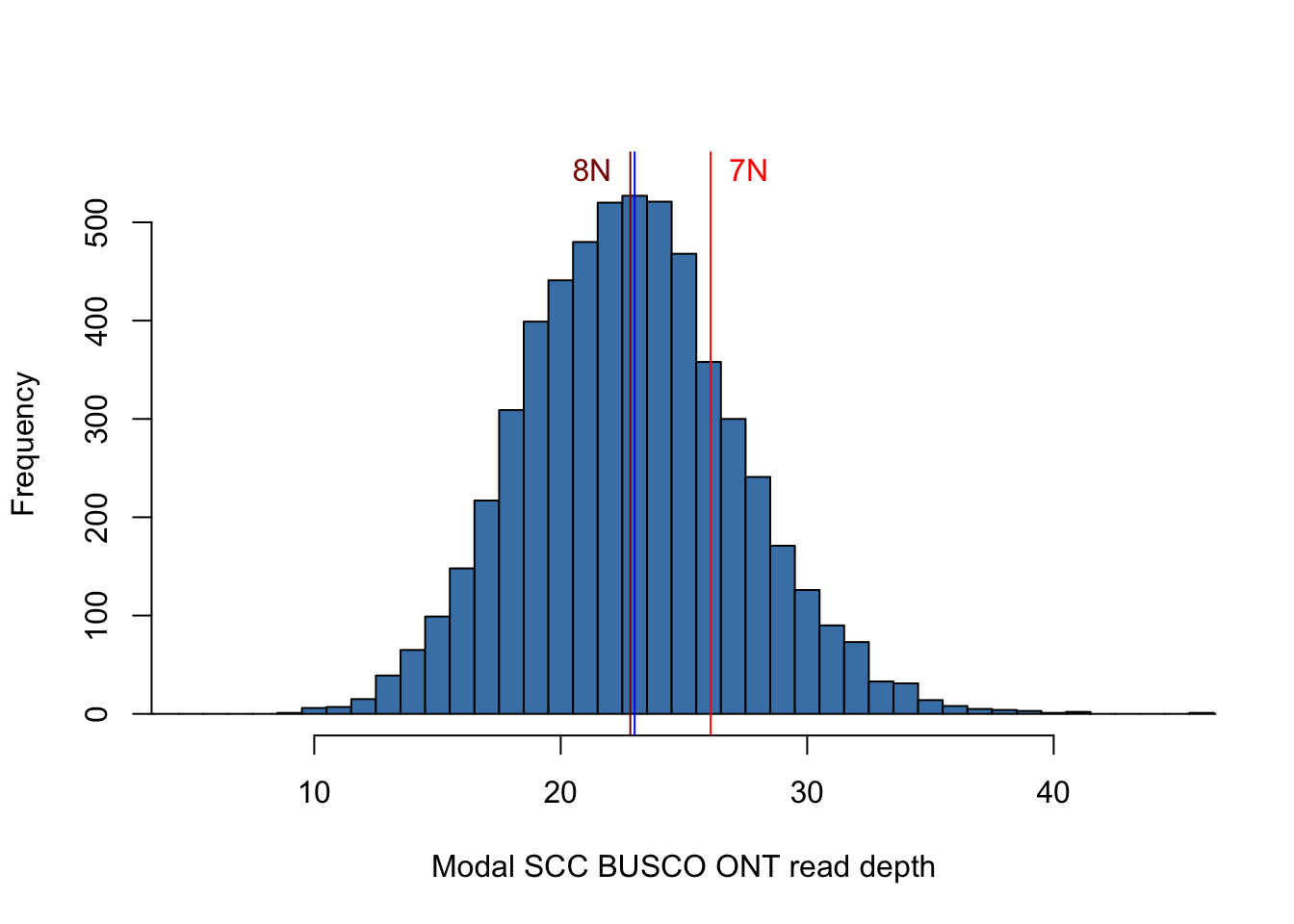


**Supplementary File 1 - Figure 3. Modal read depths across Single Copy Complete BUSCO gene regions.** Blue line, median modal read depth; Red line, predicted single copy read depth for 7 or 8 repeat units. **(a)** PacBio subreads. **(b)** ONT reads.

1. Li H, Handsaker B, Wysoker A, Fennell T, Ruan J, Homer N, et al. The Sequence Alignment/Map format and SAMtools. Bioinformatics. 2009;25 16:2078-9. doi:10.1093/bioinformatics/btp352.

2. Li H. Minimap2: pairwise alignment for nucleotide sequences. Bioinformatics. 2018;34 18:3094-100. doi:10.1093/bioinformatics/bty191.

3. Simao FA, Waterhouse RM, Ioannidis P, Kriventseva EV and Zdobnov EM. BUSCO: assessing genome assembly and annotation completeness with single-copy orthologs. Bioinformatics. 2015;31 19:3210-2. doi:10.1093/bioinformatics/btv351.
