# Supplementary material for: Canfam_GSD: De novo chromosome-length genome assembly of the German Shepherd Dog (Canis lupus familiaris) using a combination of long reads, optical mapping, and Hi-C
Source: Gigascience. 2020 Apr 1;9(4):giaa027. doi: 10.1093/gigascience/giaa027 (PMC7111595; doi:10.1093/gigascience/giaa027)
Supplement: giaa027_Supplemental_Files [file giaa027_supplemental_files.zip › Supp_File2.docx]

**Supplementary File 2: Bionano AMY2B Methods**

Single molecules of Bionano data was *de novo* assembled using Bionano Solve v3.4_06042019a and RefAligner v8949.9232rel with 20 iterations of extend and split. The assembly was phased using the -Haplotype flag along with a customised set of parameters. The resulting consensus map set was aligned to the GSD assembly as well as the two sequence constructs containing seven or eight copies of the AMY2B repeat. Alignments were performed following *in silico* digestion of the sequence data into genome maps using the DLE1 enzyme motif. Visualisations of Bionano data were performed using Bionano Access v1.4.
