# Supplementary material for: Canfam_GSD: De novo chromosome-length genome assembly of the German Shepherd Dog (Canis lupus familiaris) using a combination of long reads, optical mapping, and Hi-C
Source: Gigascience. 2020 Apr 1;9(4):giaa027. doi: 10.1093/gigascience/giaa027 (PMC7111595; doi:10.1093/gigascience/giaa027)
Supplement: giaa027_Supplemental_Files [file giaa027_supplemental_files.zip › Supp_File3.docx]

**Supplementary File 3: 10X Genomics Chromium sequencing: Detailed Methods**

One chromium read consists of multiple standard Illumina read-pairs that share the same barcode and that originating from the same roughly 100 kb DNA fragment. The chromium read alignment file was filtered for misaligned read-pairs and read-pairs mapping outside of contig start and end regions. I.e. Read-pairs were removed that did not align within their expected distance and orientation (SAM flag proper pairs, 8% of initial 795 million), were secondary or supplementary (additional 2%), had no barcode (additional 0.6%) and were mapping more than 200k away from contig start and end positions (additional 79%), resulting in 78 million read-pairs (10%) and 10 million linked-read alignments. A chromium linked-read being aligned to a contig's start or end has the potential to reach into another contig providing evidence for both contigs to be next to each other in the genome. Only strongly anchored linked-reads were considered, by discarding alignments on contigs with less than 5 read-pairs (62% of 10 million) and less than 50% of read-pairs being uniquely aligned (XA tag, additional 7%). Further, linked-reads that are likely already fully contained in a contig were removed by discarding those not reaching into the first or last 20kb of a contig (additional 18%). The observed distance between read-pairs of a chromium linked-read was mostly smaller than 20kb. From the filtering we retained 1,2 million linked-read fragments used for scaffolding. Because the same barcode can occur in multiple linked-reads, all possible contig connections suggested per barcode were computed. Connections between two contigs were established if they reciprocally shared the majority of links, i.e. the majority of terminal linked-reads of contig A pointing to contig B and vice versa, the majority being defined as more than 50% of the terminal linked-reads of a contig and more than 50% of total read-pairs that make up the linked-reads.
