# Supplementary figures and images for: Canfam_GSD: De novo chromosome-length genome assembly of the German Shepherd Dog (Canis lupus familiaris) using a combination of long reads, optical mapping, and Hi-C
Source: Gigascience. 2020 Apr 1;9(4):giaa027. doi: 10.1093/gigascience/giaa027 (PMC7111595; doi:10.1093/gigascience/giaa027)

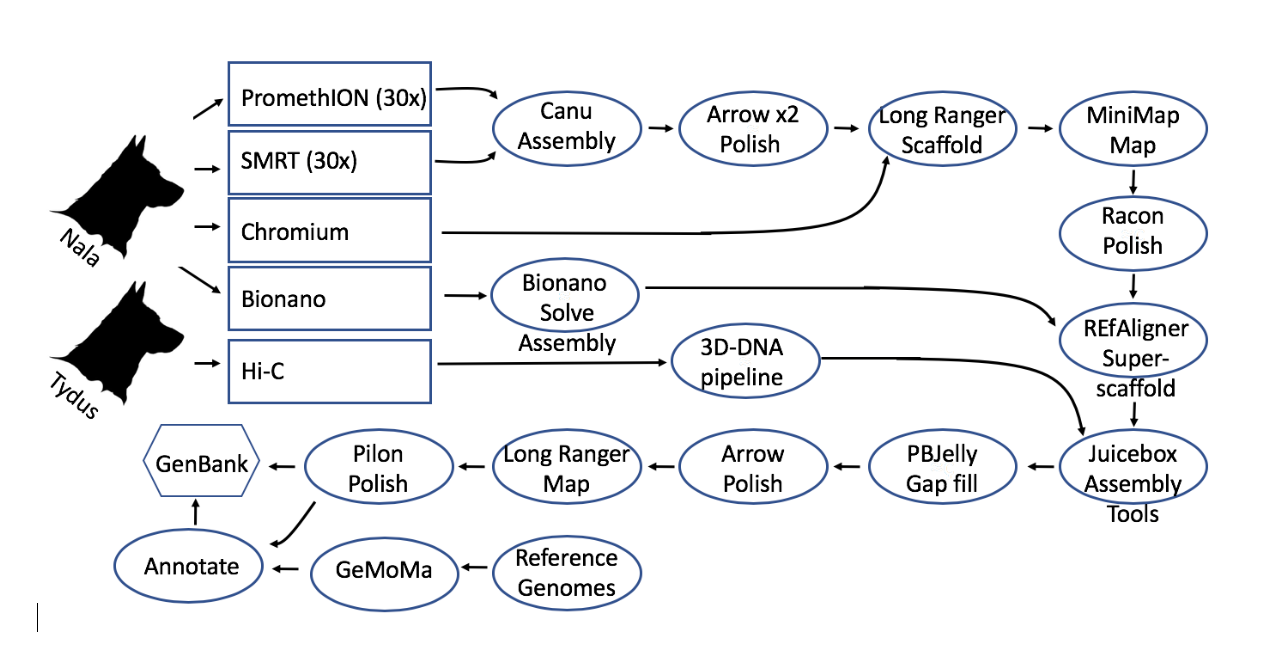

Supplement: giaa027_Supplemental_Files [file giaa027_supplemental_files.zip › Supp_Fig1.tiff]

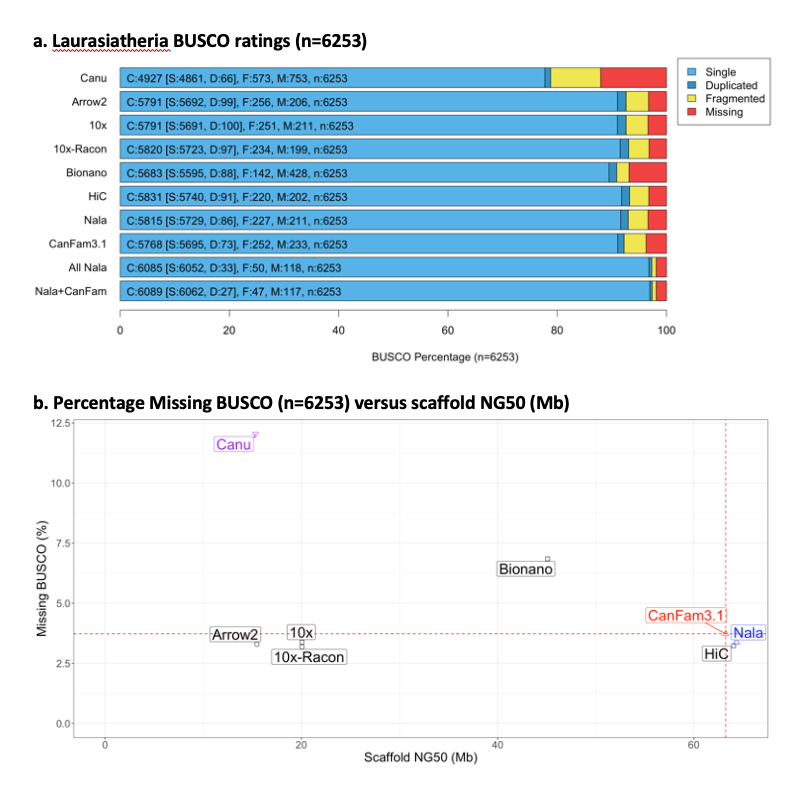

Supplement: giaa027_Supplemental_Files [file giaa027_supplemental_files.zip › Supp_Fig2.tiff]

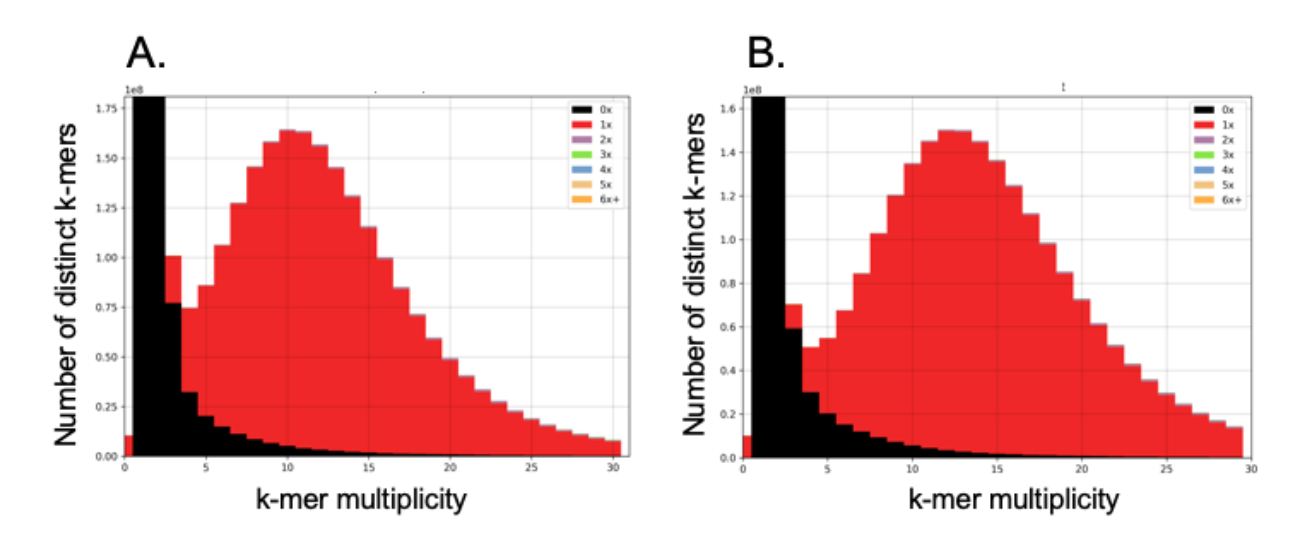

Supplement: giaa027_Supplemental_Files [file giaa027_supplemental_files.zip › Supp_Fig3.tiff]

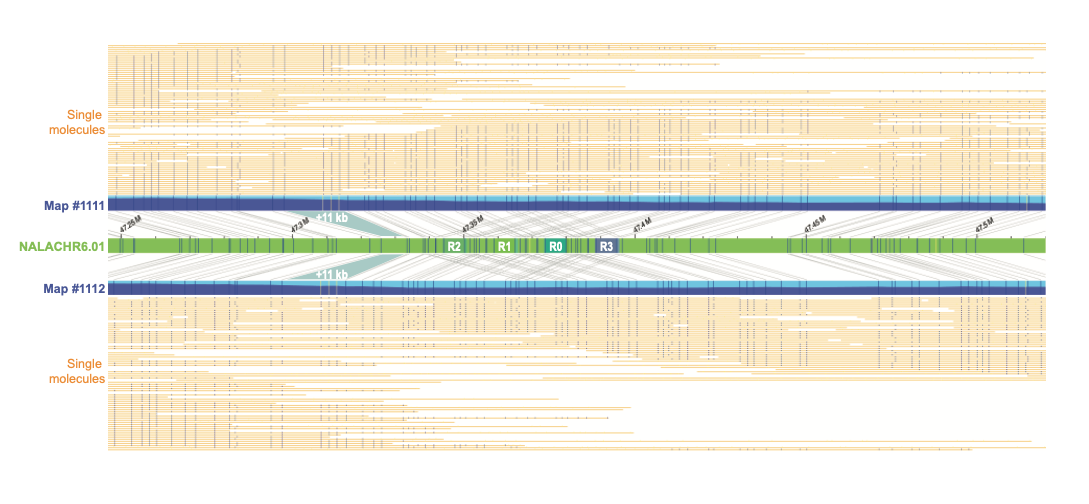

Supplement: giaa027_Supplemental_Files [file giaa027_supplemental_files.zip › Supp_Fig4.tiff]

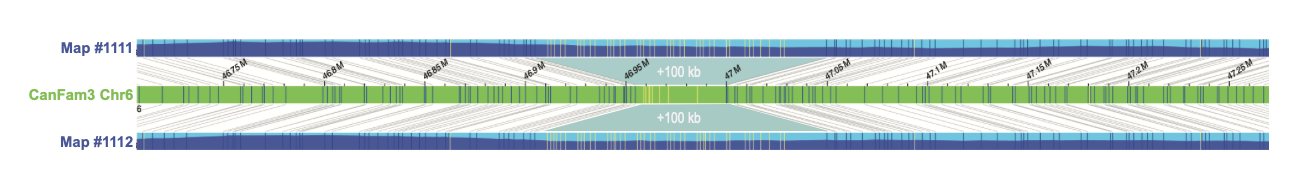

Supplement: giaa027_Supplemental_Files [file giaa027_supplemental_files.zip › Supp_Fig5.tiff]

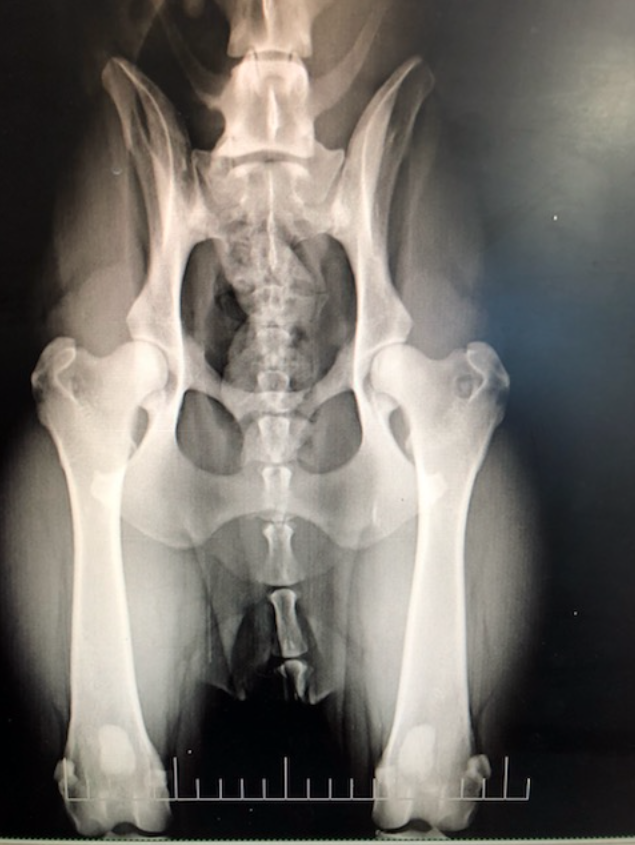

Supplement: giaa027_Supplemental_Files [file giaa027_supplemental_files.zip › Supp_Fig6.tiff]

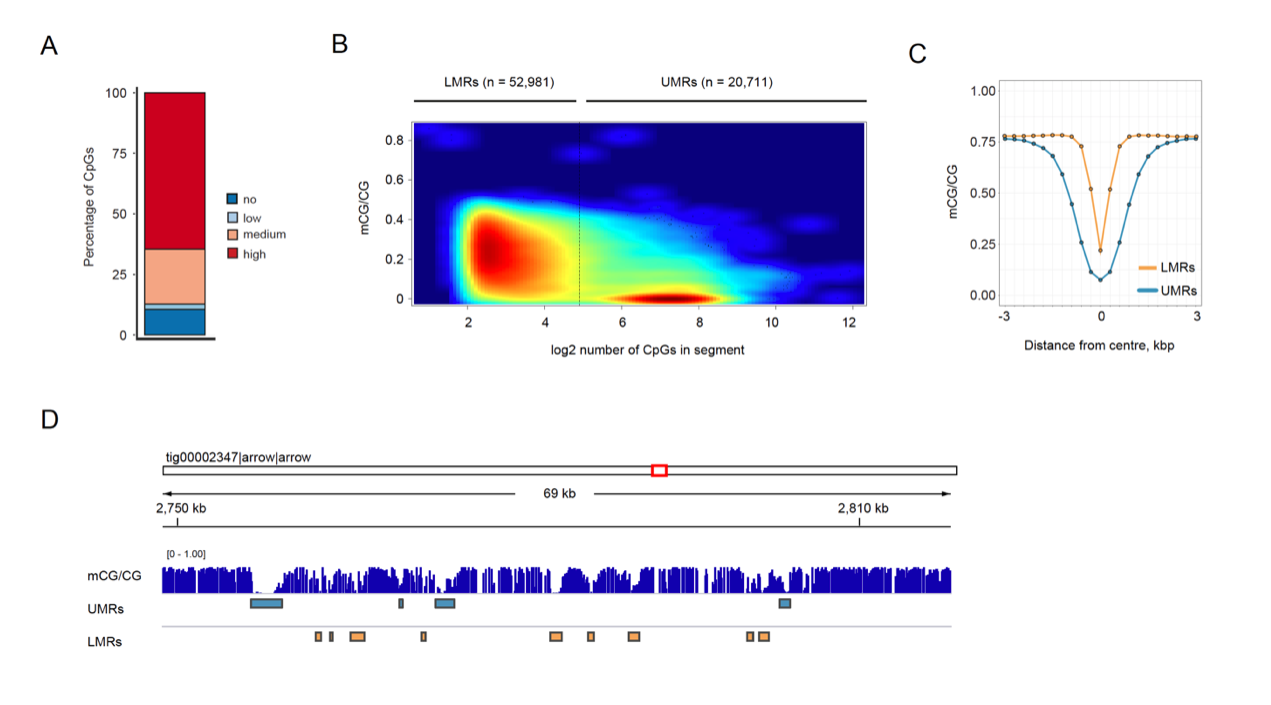

Supplement: giaa027_Supplemental_Files [file giaa027_supplemental_files.zip › Supp_Fig7.tiff]
