# Supplementary material for: Canfam_GSD: De novo chromosome-length genome assembly of the German Shepherd Dog (Canis lupus familiaris) using a combination of long reads, optical mapping, and Hi-C
Source: Gigascience. 2020 Apr 1;9(4):giaa027. doi: 10.1093/gigascience/giaa027 (PMC7111595; doi:10.1093/gigascience/giaa027)
Supplement: giaa027_Response_to_Reviewer_Comments_Original_Submission [file giaa027_response_to_reviewer_comments_original_submission.pdf]

Author's Response To Reviewer Comments

Close

Dear Editor;

Thank you for giving us the opportunity to respond to the Reviewers comments. Our replies are in bold below and appropriately in text. We now hope the manuscript is acceptable for publication in GigaScience.

We found the reviewers comments highly constructive and so they are acknowledged. In the Acknowledgements we also recognise comments from James Ferguson.

Due to his scientific contribution to the Promethion sequencing Martin A. Smith is now included as a co-author. Additionally, author Richard J. Edwards position in the manuscript was adjusted to acknowledge his significant contribution during the review process. All affected authors have been notified and agreed to this change.

For clarity we have re-drawn Supplementary Figure 1.

Also, added key words to the manuscript. The words we have included are:
Hi-C, long read sequencing, optical mapping, de novo genome assembly, canine hip dysplasia, DNA Zoo.

Yours truly,
J. William O. Ballard
University of New South Wales
Sydney
Australia, 2052

GIGA-D-19-00364
De novo genome assembly of German Shepherd Dog (Canis lupus familiaris)
Matt A Field, PhD; Benjamin D. Rosen; Olga Dudchenko; Eva K.F. Chan; Andre E. Minoche; Kirston Barton; Ruth J. Lyons; Daniel Enosi Tuipulotu; Richard J. Edwards; Vanessa M. Hayes; Arina Omer; Zane Colaric; Jens Keilwagen; Ksenia Skvortsova; Ozren Bogdanovic; Erez Lieberman Aiden; Timothy P.L. Smith; Robert A. Zammit; J. William O. Ballard
GigaScience

Dear Professor Ballard,

Your manuscript "De novo genome assembly of German Shepherd Dog (Canis lupus familiaris)" (GIGA-D-19-00364) has been assessed by our reviewers. Although it is of interest, we are unable to consider it for publication in its current form. The reviewers have raised a number of points which we believe would improve the manuscript and may allow a revised version to be published in GigaScience.

Their reports, together with any other comments, are below. Please also take a moment to check our website at https://www.editorialmanager.com/giga/ for any additional comments that were saved as attachments.

In addition, please register any new software application in the bio.tools and SciCrunch.org databases to receive RRID (Research Resource Identification Initiative ID) and biotoolsID identifiers, and include these in your manuscript. This will facilitate tracking, reproducibility and re-use of your tool.

If you are able to fully address these points, we would encourage you to submit a revised manuscript to GigaScience. Once you have made the necessary corrections, please submit online at:

https://www.editorialmanager.com/giga/

If you have forgotten your username or password please use the "Send Login Details" link to get your login information. For security reasons, your password will be reset.

Please include a point-by-point within the 'Response to Reviewers' box in the submission system. Please ensure you describe additional experiments that were carried out and include a detailed rebuttal of any criticisms or requested revisions that you disagreed with. Please also ensure that your revised manuscript conforms to the journal style, which can be found in the Instructions for Authors on the journal homepage. If the data and code has been modified in the revision process please be sure to update the public versions of this too.

The due date for submitting the revised version of your article is 17 Mar 2020.

I look forward to receiving your revised manuscript soon.

Best wishes,

Hongling Zhou
GigaScience
www.gigasciencejournal.com

Reviewer reports:

Reviewer #1: The manuscript" De novo genome assembly of German Shepherd Dog (Canis lupus familiaris)" presents a new genome assembly for the dog model community. The authors chose to sequence one of the most famous breeds, the German Shepherd, with recent technologies including long read sequencing using PacBio SMRT, and ONT PromethION sequencing. Their goal is to improve the genome reference used by the dog community. They also explored the methylome and produced HI-C library using a second dog. They provide detailed methodology and used the example of the AMY2B locus to illustrate how this new reference sequence can be used to capture missing data, particularly around structural variations such as copy number variations. As a resource paper, this work has great potential and will be heavily citable. Regarding the quality of the work, I have a few comments/questions:

1) Taking a look on NCBI, I noticed two other full high-quality genome assemblies available for Canis lupus familiaris, since CanFam3.1 was initially made available in 2011. Did the authors try to contact the two other groups to compare data, as you did with canfam3.1? This manuscript gives rise to a major question in the dog community concerning the nomenclature used for new reference genomes, i.e. since many new references are being produced, I recommend that the authors name their assembly in a way that is easily recognizable and discernable. Using CanFam 4 is far from ideal since all previous dog genome references (canfam1, 2, 3) were produced using the same Boxer, Tasha. An alternative would be to use a name like CanfamGSD.1, for example? This sets the stage for other additional de novo assemblies. Each can retain the CanFam designation, but an abbreviation to indicate which dog/breed it is. This issue must be dealt with.

REPLY: We did approach other groups but were told unequivocally that they were not interested. We have added Canfam_GSD to the title and Findings section of the abstract.

I also wondering how these data will be shared with the scientific community. Do you also plan to submit your data to established genome browsers commonly used by the scientific community (i.e: Ensembl, UCSC...?). Presumably yes in order that it will be widely used. The paper should contain this information.

REPLY: The genome is present at http://www.dnazoo.org/assemblies/Canis_lupus_familiaris_German_Shepherd and is also in a locally hosted Apollo browser. Like our Dingo genome assembly it will be transferred to Ensembl.

2) I noticed that there is no data for the mitochondrial DNA. Is it a choice from the authors or a technical limitation? The actual Boxer reference CanFam3.1 has mitochondrial sequence as does the Great Dane sequence submitted to NCBI this year. Since the authors proposed their new genome as a future dog genome reference, can they explain in one or two sentences why they did not work on the

mitochondrial DNA.

REPLY: In addition to the nuclear genome, the mitochondrial genome was assembled and has been uploaded to GigaScience for immediate download. It also has been deposited to NCBI and is being processed. Added to the manuscript L155
The mitochondrial genome assembly was also assembled and has been uploaded to GigaScience for immediate download. It has been deposited to NCBI and is being processed.

3) Concerning the annotation: did the authors improve the annotation of keratin clusters (CFA9, for example) or the olfactory receptor clusters that are typically poorly annotated? If yes, please discuss. These are some of the biggest flaws in the previous genome assembly and it is imperative that this be highlighted in this paper. Alternatively, if large paralog gene families continue to present a problem, it is worth mentioning. Also, in order to provide the most completely annotated genome reference, I recommend the authors include all non-coding RNA (miRNAs, lncRNAs) recently published data (See Wucher et al, Nuc Acid.Res 2017, Megquier K et al, Genes 2019). It could be also interesting to compare your data with the observations published last year (Holden et al, Sci Rep. 2018) which identified missing gene sequences associated with diseases using unmapped sequences.

REPLY regarding OF and keratin: We have added a new section on L269 in the Methods after “Pancreatic amylase (AMY2B) analysis”.

"Olfactory receptor and Keratin cluster analysis

Correct annotation of the olfactory and keratin clusters in canines has been problematic but it is important for research on canid health and evolution [21, 30, 31]. Dogs are macrosmatic animals that rely highly on their sense of smell. Yet, the molecular basis of such prominent chemosensory capacities remains largely unknown. The ability to detect and discriminate the multitude of odors in vertebrates is mediated by a superfamily of G-protein-coupled olfactory receptor (OR) proteins [32]. Based on the description in the reference annotations, we filtered all mRNAs of the references that contain in their description the regular expression “olfactory receptor”. We then extracted the number of mRNAs and genes per reference organism. Subsequently, we used the IDs to filter the GSD annotation and counted the number of predicted mRNAs and genes. This procedure identifies 1250 mRNAs and 933 genes in the GSD and 849 mRNAs and 804 genes in the boxer. Quignon et al. [30] identified five amino acid patterns characteristic of ORs in the canine genome and retrieved 1,094 dog genes (872 genes and 222 pseudogenes).

Keratins are filament proteins of the epithelial cytoskeleton and are essential for normal skin homeostasis. Over time the genes encoding keratins have undergone multiple rounds of duplication with high similarity between different keratin paralogs [31]. Analogously to the olfactory receptor study, we filtered all mRNAs of the references that contain in their description the keyword “keratin \d”. This procedure identifies 118 mRNAs and 83 genes in the GSD and 73 mRNAs and 55 genes in the boxer. Balmer et. al. [31] investigated the National Center for Biotechnology Information (NCBI) (dog annotation release 103) gene predictions for the canine gene clusters to RNA-seq data that were generated from adult skin of five dogs and adult hair follicle tissue of one dog and annotated 61 putatively functional keratin genes in the dog."

Also, to link with this inclusion we have added a new paragraph in the Discussion L323.

"The assembly is expected to enable the selection of GSDs for particular duties including police work where their sensitive nose is frequently used to discriminate odors. Robin et al. [30] analyzed the nucleotide sequences of 109 OR genes (102 genes and seven pseudogenes) in six different breeds including GSDs. Most generally they show that OR genes are highly polymorphic, with a mean of one SNP per 577 nucleotides. However, the degree of polymorphism observed is highly variable, with some OR genes having few if any SNPs and others being highly polymorphic (1 SNP/122 nt). Yang et al. [33] conducted a preliminary study of 22 SNPs from the exonic regions of 12 OR genes in GSDs and found a significant correlation between SNP genotypes of DR genes and olfactory abilities of dogs."

REPLY regarding rRNA: We have not included RNA data in this study and suggest that annotating all non-coding RNA is beyond the scope of the study. We have now, however, annotated the rRNA genes and added a section to the methods (L683) and a file with annotations is now available (rRNA_predictions.gff)

"rRNA genes were predicted with Barrnap v0.9 (https://github.com/tseemann/barrnap) in the eukaryotic mode, implementing Perl v5.28.0, HMMer v3.2.1 [66] and BEDTools v2.27.1 (https://github.com/arq5x/bedtools2). "

REPLY regarding Holden et al. contigs: We have mapped all of the contigs from the Holden et al. study onto both assemblies and demonstrated a marked improvement in coverage with GSD compared to CanFam3.1.
Analysis of novel contigs assembled by Holden et al. from canine reads that failed to map to CanFam 3.1 further supports the greater completeness of GSD. For all three dog breeds assembled by Holden et al., a greater proportion of the combined contigs map on to GSD than CanFam3.1 (94.3% vs 85.4% Border Collie; 77.0% vs 33.9% Bearded Collie; 76.8% vs 42.1% Entlebucher Sennenhund).
Note: This work was not included in the final manuscript.

4) Concerning the AMY2B story, the authors describe how Bionano technology analyzed the relevant CNV but did not discuss their results in an evolutionary context, such as what are the consequences of higher copy number. What hypothesis can be made concerning the complete loss of AMY2B locus observed in CanFam3.1 in comparison with the GSD genome? Is it related to the breed creation (Boxer vs GSD), or just a polymorphic CNV in modern breeds without any particular selection in current times? For example, it could be very interesting to see if, using your genome reference, you can use the GSD genomes already published and available on SRA and other related breeds to: 1) confirm your observations on this locus, and 2) explore breeds that you cite in your discussion (L490-496), checking how many copies each has on this locus. This will provide insight regarding about the evolutionary position of the GSD as regards the two cited papers L493-494 (Bigi et al 2015, Parker et al, 2017).

REPLY: The AMY2B story is an interesting and complex one. There is an expansion in all domestic breeds (including Boxer) relative to the wolf. We are currently de novo sequencing Desert and Alpine Australian dingoes to gain greater insight into this question. We suggest additional analyses is beyond the scope of the current manuscript.

5) This comment follows my first comment and is maybe beyond this paper: I noticed the first author released the Dingo genome (Canis lupus dingo), submitted on NCBI in June 2018 (Bioproject: PRJNA477859) and already available on Ensembl. I did not find an associated paper to this work, so I am wondering if authors could make a comparison between both Dingo and GSD in this paper, highlighting potential genomic differences which would support the last sentence of your abstract: "This resource will enable further research related to canine diseases, the evolutionary relationships of canids, and other aspects of canid biology".

REPLY: You are correct the Canis lupus dingo was submitted on NCBI in June 2018. Currently, we are in the process of improving the assembly of the Desert Dingo (v2) so that more robust comparisons can be made with the Wolf and domestic dogs (including the AMY2B expansion story, which is still unfolding). As indicated above we are also de novo sequencing the Alpine Dingo and and aim to also include a new de novo Basenji assembly.

I noticed some typos:

L98: "best-known".
REPLY. Corrected

L186: Pancreatic amylase (AMY2B) analysis.
REPLY. Corrected

L221-223: For a better understanding, I recommend your write this sentence like this:
"Alignment results confirmed the presence of seven repeat units, showing a perfect alignment to the seven-copy sequence construct (Figure 2A), but a "deletion" of one repeat unit relative to the eight-copy construct (Figure 2B)."
REPLY. Corrected OK.

L290-292: Can you re-write this sentence please - I do not understand what is largely methylated and largely unmethylated?
REPLY: Rewritten as “In concordance with other adult vertebrates [40, 41], the GSD genome displays a typical bimodal DNA methylation pattern with over 60% of CpG dinucleotides being methylated at levels

higher than 80% (hypermethylated) and 12% of CpG dinucleotides being methylated at 20% or lower (hypomethylated)."

L297-299: you should specify "in other models" because these references did not work on dogs.
REPLY: OK. added.

L315: Please correct " Saphyr instrument"
REPLY. Corrected

L465: remove uppercase for "annotation".
REPLY. Corrected

Supplementary Table 1: typo in title: "statistics" when you open the file.
REPLY. Corrected

Reviewer #2: General comments
The authors report on the de novo genome assembly and annotation of a female German shepherd, a popular and well-known breed of domestic dog. The chromosome-length genome was generated using a combination of sequences obtained from PacBio and ONT long reads,10X Genomics Chromium libraries, Bionano Genomics optical maps and Hi-C libraries. The assembly was produced using an iterative workflow in which the long-read data were first assembled into contigs and these were then successively scaffolded using the 10X Genomics Chromium data, optical maps and Hi-C data. Each stage of the assembly was evaluated in terms of continuity and completeness as judged by BUSCO scores. The hybrid assembly was subjected to three rounds of rigorous polishing to produce an accurate and highly continuous assembly. In fact, the German shepherd dog assembly is 80X more contiguous than the standard domestic dog reference genome, CanFam version 3.1 (based on the boxer breed). As such, the newly
reported assembly achieves an important benchmark in terms of genome assembly quality.

Specific comments
The manuscript is generally well written and the methods of the genome sequencing and assembly are thoroughly presented in detail in both the main text and the Supplementary Materials. The newly reported assembly represents one of the most contiguous genome assemblies yet generated for a mammalian species outside of humans and will no doubt provide an important resource for countless studies in addition to the CanFam version 3.1 assembly which has been the primary assembly for many years. The in-depth annotation and analysis of the AMY2B copy number is especially thorough and well done. The paper will no doubt stimulate great interest by researchers in mammalian genomics as well as the general public. I recommend the manuscript be accepted for publication once the authors have addressed specific comments that I think would improve the clarity and impact of the manuscript, as described below.

1. There are no page numbers or line numbers, which makes it unnecessarily difficult to provide comments on the manuscript. Therefore, I will reference my comments according to the page number and manuscript section based on the pdf version of the manuscript. Please add page numbers and line numbers in future versions of the manuscript.
REPLY: OK. added.

2. In the provided manuscript, the Methods section was placed before the Discussion section. As this has been submitted as a Research article, the Methods section should be moved to after the Discussion section.
REPLY: Moved (but did not include track changes in this cut-and-paste).

3. Contig assembly, scaffolding and polishing were evaluated using N50 and BUSCO scores. However, I recommend the authors also assess the German Shepherd dog assembly using the K-mer Analysis Toolkit (KAT; Mapleson et al. 2017 Bioinformatics 33: 574-576 and https://github.com/TGAC/KAT). KAT uses k-mer frequencies to profile errors, GC-bias, and other metrics along with providing quality control checking of assemblies at different stages. This would involve using the PacBio and ONT long reads as well as the de-barcoded raw reads from the 10X Genomics Chromium sequencing that were generated.

REPLY: We thank the reviewer for this suggestion and applied KAT to our assembly. The per-base error rates were too high for PacBio or ONT reads to yield useful kmer analysis, but the 10x reads provided useful additional QC. KAT does not provide an explicit way to compare different stages of the assembly,

so instead we have used it for QC of our final assembly. We have added the following paragraph (L179) and included a new Supplementary Figure (Supplementary Figure 3). This addition necessitated renumbering of figures.

"Additional k-mer analysis of the final assembly was performed using KAT v2.4.2 [26]. KAT comp was used to compare k-mer frequencies from the 10x reads (16 bp barcode trimmed from read 1) with their copy number in the assembly. This comparison revealed no sign of missing data nor large duplications, including retention of haplotigs (Supplementary Figure 3)."

4. Title: I suggest revising the title of the manuscript so that it is less generic and more descriptive of the methods/technologies used:
"De novo chromosome-length genome assembly of the German Shepherd Dog (Canis lupus familiaris) using a combination of long reads, optical mapping and Hi-C".

REPLY: We have modified the title as you suggested and added “Canfam_GSD” to the start of the title at the suggestion of reviewer 1. The new title reads “Canfam_GSD: De novo chromosome-length genome assembly of the German Shepherd Dog (Canis lupus familiaris) using a combination of long reads, optical mapping and Hi-C”.

5. Page 8, Results, Workflow: "Contigs were assembled using SMRT and ONT sequencing and then polished to minimize error propagation." Although the authors refer to Supplementary Figure 1 in this paragraph, for this sentence, the authors should reference the program(s) used for polishing and also the section of the Methods where this is detailed.

REPLY: OK, programs referenced and section of the Methods where this is detailed supplied.

6. Page 9, Results, Assembly stats / completeness: "…against Laurasiatheria_ob9 (n=6,253)…" This should be revised as "…against the Laurasiatheria_ob9 data set (n=6,253)…"
REPLY: OK. Added.

7. Page 10, Table 1: In column three (CanFam3.1) and the row, "BUSCO complete (genome)", the value 91.10.8% single copy doesn't make sense. I assume the authors mean 91.1%. Please correct.
REPLY: OK. Thanks.

8. Page 10, Results: "Based on the existing CanFam3.1 annotation and the GSD annotation provided by GeMoMa…" Please provide a reference for the GeMoMa software.
REPLY: OK add GeMoMa ref.

9. Page 11, Results: "Pacreatic amylase (AMY2B) analysis" should be corrected as "Pancreatic amylase (AMY2B) analysis".
REPLY: OK. Corrected.

10. Page 11, Results, Pancreatic amylase (AMY2B) analysis: "The longest read in the region covered three plus copies…" This description of the number of copies is vague. The following revision is suggested: "The longest read in the region covered between three to [insert maximum number observed] copies…"
REPLY: Replaced with The long sequencing reads in the region covered no more than three complete copies…

11. Page 11, Results, Pancreatic amylase (AMY2B) analysis: "Further examination of this region was attempted using both the Bionano genome map…" Suggested revision: "Further examination of this region was attempted using both the Bionano optical map…"
REPLY: OK

12. Page 13, Methods, Sampling: Nala the German Shepherd Dog: "Nala had a combined hip score of 3 (1 on LHS and 2 on RHS) when the x-ray was taken at 5 years of age…" Not all readers may understand the LHS and RHS notation. Suggested revision: "Nala had a combined hip score of 3 (1 on the left hand side and 2 on the right hand side) when the x-ray was taken at 5 years of age…"
REPLY: OK.

13. Page 14, Methods, Pacific Bioscience Single Molecule Real-Time (SMRT) sequencing: "…and molecular integrity was assessed using pulse-field gel electrophoresis." In the interests of repeatability,

the authors should provide more details about the PFGE experiment(s) including the instrument used (if commercial instrument), gel type and concentration, the voltage, run time, and the amount of DNA run and DNA standards used.
REPLY: Added L382 "DNA integrity was assessed by the Sage Science Pippin Pulse. A 0.75% KBB gel was run on the 9hr 10-48kb (80v) program. DNA ladder used was the Invitrogen 1kb Extension DNA ladder (cat 10511-012). 150ng of DNA was loaded on the gel."

14. Page 14, Methods, Pacific Bioscience Single Molecule Real-Time (SMRT) sequencing: "…and at the Arizona Genomic Institute, University of Arizona (four SMRT cells with a 11Gb of data: NOTE: short read lengths were due to DNA shearing of the DNA during shipping from Australia to Arizona)." Suggested revision: "…and at the Arizona Genomic Institute, University of Arizona (four SMRT cells with a total of 11Gb of data; NOTE: short read lengths were due to DNA shearing of the DNA during shipping from Australia to Arizona)."
REPLY: OK. Thanks.

15. Page 15, Methods, 10X Genomics Chromium sequencing: The authors should reference Supplementary File 3 at the end of this paragraph, which provides a more detailed description of the methods used for the 10X Genomics Chromium sequencing. Also, Supplementary File 3 should be retitled as "10X Genomics Chromium sequencing: Detailed Methods."
REPLY: OK.

16. Page 15, Methods, 10X Genomics Chromium sequencing: "…was barcoded from high-molecular-weight DNA according to manufacturers recommended protocols." Suggested revision: "…was barcoded from high-molecular-weight DNA according to the manufacturer's recommended protocols." Also, the authors should provide a reference for these protocols (i.e., a manual document or website URL).
REPLY: Now reads (L423)
"Protocol used was the Chromium Genome Reagent Kits v2 User Guide, manual part number CG00043 Rev B available here: https://support.10xgenomics.com/genome-exome/library-prep/doc/user-guide-chromium-genome-reagent-kit-v2-chemistry."

17. Page 15, Methods, 10X Genomics Chromium sequencing: "QC was performed using LabChip GX and Qubit." Please provide manufacturer information and location for both of these instruments.
REPLY: Added on L426
"QC was performed using LabChip GX (PerkinElmer, MA, USA) and Qubit 2.0 Flurometer (Life Technologies, CA, USA) at the Kinghorn Centre for Clinical Genomics."

18. Page 15, Methods, Methylome: "…using MethylSeekR algorithm [27]." Suggested revision: "…using the MethylSeekR algorithm [27]."
REPLY: OK.

19. Page 16, Methods, Bionano optical mapping: "Multiple cycles were performed to reach average raw genome depth of coverage of 190X." Suggested revision: "Multiple cycles were performed to reach an average raw genome depth of coverage of 190X."
REPLY: OK.

20. Page 17, Methods, 10X Chromium linked-reads: "The arrow polished SMRT/ONT assembly was scaffolded…" Suggested revision: "The Arrow-polished SMRT/ONT assembly was scaffolded…"
REPLY: OK.

21. Page 17, Methods, 10X Chromium linked-reads: "The 10X data was aligned using the linked-read analysis software provided by 10X Genomics, Long Ranger, v2.1.6 (https://www.10xgenomics.com/), misaligned reads and reads not mapping to contig ends were removed, all possible connections between contigs were computed keeping best reciprocal connections." This sentence is overly complicated and doesn't make sense as written. I suggest breaking this into two sentences and revising as follows: "The 10X read data was aligned using the Long Ranger, v2.1.6 software (https://www.10xgenomics.com/). Misaligned reads and reads not mapping to contig ends were removed, and all possible connections between contigs were computed by keeping the best reciprocal connections."
REPLY: OK suggested correction implemented. Thanks, that’s better now.

22. Page 18, Methods, Optical mapping for super-scaffolding using Bionano data: "Alignments indicating conflict between the sequence and optical maps, and hence suggestive of mis-assembly, were evaluated

such that, conflicts supported by single-molecule optical maps (thus supporting optical map) would cause the sequence map to be "cut" at the conflict point, else the optical map was "cut"." This meaning and method described in the second part of this sentence is not clear (after "were evaluated such that"). Please revise to improve clarity.
REPLY: Replaced on L525 with "Alignments indicating conflict between the sequence and optical maps, and hence suggestive of mis-assembly, were resolved. Specifically, optical maps supported by at least ten single molecules at the conflict site were indicative of sequence mis-assembly, and so the sequence map would be "cut" (split) at the conflict point. In contrast, insufficient single molecule support for the optical map was indicative of optical map assembly error, and so the optical map would be "cut" at the conflict site."

23. Page 19, Methods, Gap filling: "After scaffolding and correction, all raw reads were aligned to the assembly…" Which raw reads are the authors referring to here? The raw reads from the 10X Genomics Chromium sequencing? Please specify.
REPLY: "After scaffolding and correction, all raw SMRT and ONT reads were aligned to the assembly."

24. Page 20, Methods, Polishing Round 2: "…and correcting the single nucleotide polymorphism's (SNP) and indels using Pilon [43]." Suggested revision: "…and correcting the single nucleotide polymorphisms (SNPs) and indels using Pilon [43]."
REPLY: OK.

25. Page 20, Methods, Low-coverage filter: "Any scaffolds with median coverage less than 3 (e.g., less than 50% of the scaffold covered by at least three reads) were filtered as Low Coverage." To improve clarity, I suggest the following revision: "Any scaffolds with median coverage less than 3 (e.g., less than 50% of the scaffold covered by at least three reads) were filtered out as Low Coverage scaffolds."
REPLY: OK. Thanks.

26. Pages 20-21, Methods, Purge Haplotigs analysis - round 1 and round 2: "Subreads were re-mapped on to the remaining 837 scaffolds…" and "Subreads were re-mapped on to the remaining 558 scaffolds…" It's not clear where these remaining scaffolds originate from in the two paragraphs about purging of the haplotigs. Are these the scaffolds remaining after primary assembly using the PacBio+ONT+10X Genomics Chromium+Bionano optical map+Hi-C? Also, after the first round of purging, it seems only 279 scaffolds were processed, leaving 558 scaffolds to be processed in the second round. Is this correct? The authors need to better clarify these points so that they are more understandable for readers.
REPLY: Added/ clarified three sentences as described below. Also, we added a small section on our final contamination screening step.

[Low-coverage filter]
Of the 1,057 Pilon-polished scaffolds, 220 scaffolds were removed in the initial Low-coverage filter, leaving 837 scaffolds.

[Purge Haplotigs analysis - round 1]
This analysis resulted in a further 11 scaffolds filtered for low coverage and 268 filtered as haplotigs or assembly artefacts, leaving 558 scaffolds.

[Purge Haplotigs analysis - round 2]
Subreads were re-mapped on to the remaining 558 scaffolds resulting in a further 128 scaffolds filtered as haplotigs or assembly artefacts leaving 430 scaffolds.

[Final Scaffold Classification]
Twenty REPEAT scaffolds corresponding to a PacBio control sequence were removed from the assembly, leaving the final 409 nuclear scaffolds plus mitochondrion. Seventeen scaffolds had small regions masked or trimmed by the NCBI Contamination screen, corresponding to a 3.4kb chunk of Escherichia coli.

27. Page 21, Methods, Purge Haplotigs analysis - round 2: "A single remaining Scaffold marked as JUNK…" Scaffold in this sentence does not need to be capitalized.
REPLY: OK.

28. Page 21, Methods, CanFam3.1 Chromosome Mapping: The PAFScaff v0.2.0 program - please provide

a reference or URL for this software.
REPLY: Reference to https://github.com/slimsuite/pafscaff added. The tool has also been registed at SciCrunch.org and bio.tools, as requested by the editor.

29. Pages 22-23, Methods, Gene prediction including Annotation of repetitive elements: Please provide NCBI accession numbers for the assembly/annotation of the nine species used for the homology-based gene prediction analyses.
REPLY: Now reads on L659:
The nine species used for the homology-based gene prediction analyses were Canis lupus familiaris (CanFam3.1; GCF_000002285.3), Vulpes vulpes (VulVul2.2; GCF_003160815.1), Felis catus (Felis_catus_9.0; GCF_000181335.3), Sus scrof (Sscrofa11.1; GCF_000003025.6), Bos taurus (ARS-UCD1.2; GCF_002263795.1), Ailuropoda melanoleuca (ASM200744v1; GCF_000004335.2), Ursus maritimus (UrsMar_1.0; GCA_000687225.1), Mus musculus (GRCm38.p6; GCF_000001635.26), and Homo sapiens (GRCh38.p13; GCA_000001405.39), which were downloaded from NCBI.

30. Page 23, Discussion: "…commonly registered KC breeds…" Please spell out "KC" when used for the first time. I assume the authors mean Kennel Club here.
REPLY: OK.

31. Page 23, Discussion: I suggest the authors expand on the discussion in the first paragraph of the Discussion section by describing in a few sentences how the German shepherd dog genome assembly can be applied "for advancing knowledge of breed specific diseases." As currently written, the paragraph only provides a description of diseases that German shepherds are pre-disposed to. Surely, the genetic etiology of some of these conditions/diseases has been previously researched. The authors should reference some of these studies and then detail how the high-quality reference genome they have generated will advance such research.
REPLY: Added new paragraph L310
"The high quality genome assembly will advance knowledge of breed specific diseases such as CHD and extend to issues related to canine personality. The severity of CHD depends on both genetic and environmental factors. In GSDs, the heritability (h2) estimates have varied from 0.1 to 0.6 [53]. To date, different study populations and methods affect the results substantially, as the reported quantitative trait locus (QTL) association and candidate genes are inconsistent between studies [54-56]. While boxers are prone to CHD the hip scores of Tasha, used for CanFam, are unknown. Further, GSD specific SNPs as well as significant CNVs and SVs are difficult to detect. In a cohort containing over 10 000 behaviorally tested GSD and Rottweiler dogs Saetre et al. [57] examined how traits are transmitted between generations. In both breeds, the pattern of co-inheritance was found to be similar for a broad personality trait previously named shyness–boldness with heritability estimated to be 0.25 in the two breeds. Currently, the underling genes involved in these behaviours are not known."

--
Please also take a moment to check our website at https://www.editorialmanager.com/giga/l.asp?i=61160&l=IAAB5AEW for any additional comments that were saved as attachments. Please note that as GigaScience has a policy of open peer review, you will be able to see the names of the reviewers.

____
In compliance with data protection regulations, you may request that we remove your personal registration details at any time. (Use the following URL: https://www.editorialmanager.com/giga/login.asp?a=r). Please contact the publication office if you have any questions.

Close
