# Supplementary material for: Canfam_GSD: De novo chromosome-length genome assembly of the German Shepherd Dog (Canis lupus familiaris) using a combination of long reads, optical mapping, and Hi-C
Source: Gigascience. 2020 Apr 1;9(4):giaa027. doi: 10.1093/gigascience/giaa027 (PMC7111595; doi:10.1093/gigascience/giaa027)
Supplement: giaa027_Response_to_Reviewer_Comments_Revision_1 [file giaa027_response_to_reviewer_comments_revision_1.pdf]

Author's Response To Reviewer Comments

Close

Dear Professor Ballard,

Your manuscript "Canfam_GSD: De novo chromosome-length genome assembly of the German Shepherd Dog (Canis lupus familiaris) using a combination of long reads, optical mapping and Hi-C" (GIGA-D-19-00364R1) has been assessed by our reviewers. Based on these reports, and my own assessment as Editor, I am pleased to inform you that it is potentially acceptable for publication in GigaScience, once you have carried out some final minor revisions suggested by our reviewers. Please also include ORCID IDs in the authorship section and cite the GigaDB DOI from the data section as soon as you have it from our curators.

RESPONSE: ORCID ids are added to the author section. GigaDB DOI is included in the "Availability of supporting data and materials" section and referenced. All typos observed by the reviewer have been corrected.

Their reports, together with any other comments, are below. Please also take a moment to check our website at https://www.editorialmanager.com/giga/ for any additional comments that were saved as attachments.

Once you have made the necessary corrections, please submit a revised manuscript online at:

https://www.editorialmanager.com/giga/

If you have forgotten your username or password please use the "Send Login Details" link to get your login information. For security reasons, your password will be reset.

Please include a point-by-point within the 'Response to Reviewers' box in the submission system. Please ensure you describe additional experiments that were carried out and include a detailed rebuttal of any criticisms or requested revisions that you disagreed with. Please also ensure that your revised manuscript conforms to the journal style, which can be found in the Instructions for Authors on the journal homepage. If the data and code has been modified in the revision process please be sure to update the public versions of this too.

The due date for submitting the revised version of your article is 12 May 2020.

We look forward to receiving your revised manuscript soon.

Best wishes,

Hongling Zhou
GigaScience
www.gigasciencejournal.com

Reviewer reports:

Reviewer #2: I have read the revised manuscript by Field et al. and find that it has been much improved compared to the original submission. The authors have done an outstanding job in providing thorough responses to my comments (and those from the other reviewer) and have revised the manuscript accordingly. The revisions have clarified specific sections that were previously opaque and the added text has nicely expanded upon certain themes related to German shepherd dog breeds. The Canfam_GSD assembly establishes an important benchmark in de novo genome assembly and will no doubt be an invaluable resource to the canine genomics community.

There are just a few minor errors in some of the new text the authors have added to the revised

manuscript that need correction. These corrections can be easily made in the proofs of the manuscript.

Line 2: Make sure to italicize Canis lupus familiaris in the title.
RESPONSE: This is changed

Line 297: "underlining genes" should be changed to "underlying genes"
RESPONSE: This is changed

Line 378: "Protocol used..." Revise as "The protocol used..."
RESPONSE: This is changed

Close
