# Supplementary material for: Canfam_GSD: De novo chromosome-length genome assembly of the German Shepherd Dog (Canis lupus familiaris) using a combination of long reads, optical mapping, and Hi-C
Source: Gigascience. 2020 Apr 1;9(4):giaa027. doi: 10.1093/gigascience/giaa027 (PMC7111595; doi:10.1093/gigascience/giaa027)
Supplement: giaa027_Reviewer_1_Report_Original_Submission — Jocelyn Plassais -- 12/6/2019 Reviewed [file giaa027_reviewer_1_report_original_submission.pdf]

Reviewer Report

Title: Canfam_GSD : De novo chromosome-length genome assembly of the German Shepherd Dog (Canis lupus familiaris) using a combination of long reads, optical mapping and Hi-C

Version: Original Submission **Date:** 12/6/2019

Reviewer name: Jocelyn Plassais

Reviewer Comments to Author:

The manuscript" De novo genome assembly of German Shepherd Dog (Canis lupus familiaris)" presents a new genome assembly for the dog model community. The authors chose to sequence one of the most famous breeds, the German Shepherd, with recent technologies including long read sequencing using PacBio SMRT, and ONT PromethION sequencing. Their goal is to improve the genome reference used by the dog community. They also explored the methylome and produced HI-C library using a second dog. They provide detailed methodology and used the example of the AMY2B locus to illustrate how this new reference sequence can be used to capture missing data, particularly around structural variations such as copy number variations. As a resource paper, this work has great potential and will be heavily citable. Regarding the quality of the work, I have a few comments/questions:

1) Taking a look on NCBI, I noticed two other full high-quality genome assemblies available for Canis lupus familiaris, since CanFam3.1 was initially made available in 2011. Did the authors try to contact the two other groups to compare data, as you did with canfam3.1? This manuscript gives rise to a major question in the dog community concerning the nomenclature used for new reference genomes, i.e. since many new references are being produced, I recommend that the authors name their assembly in a way that is easily recognizable and discernable. Using CanFam 4 is far from ideal since all previous dog genome references (canfam1, 2, 3) were produced using the same Boxer, Tasha. An alternative would be to use a name like CanfamGSD.1, for example? This sets the stage for other additional de novo assemblies. Each can retain the CanFam designation, but an abbreviation to indicate which dog/breed it is. This issue must be dealt with.

I also wondering how these data will be shared with the scientific community. Do you also plan to submit your data to established genome browsers commonly used by the scientific community (i.e: Ensembl, UCSC...?). Presumably yes in order that it will be widely used. The paper should contain this information.

2) I noticed that there is no data for the mitochondrial DNA. Is it a choice from the authors or a technical limitation? The actual Boxer reference CanFam3.1 has mitochondrial sequence as does the Great Dane sequence submitted to NCBI this year. Since the authors proposed their new genome as a future dog genome reference, can they explain in one or two sentences why they did not work on the mitochondrial DNA.

3) Concerning the annotation: did the authors improve the annotation of keratin clusters (CFA9, for example) or the olfactory receptor clusters that are typically poorly annotated? If yes, please discuss. These are some of the biggest flaws in the previous genome assembly and it is imperative that this be highlighted in this paper. Alternatively, if large paralog gene families continue to present a problem, it is

worth mentioning. Also, in order to provide the most completely annotated genome reference, I recommend the authors include all non-coding RNA (miRNAs, lncRNAs) recently published data (See Wucher et al, Nuc Acid.Res 2017, Megquier K et al, Genes 2019). It could be also interesting to compare your data with the observations published last year (Holden et al, Sci Rep. 2018) which identified missing gene sequences associated with diseases using unmapped sequences.
4) Concerning the AMY2B story, the authors describe how Bionano technology analyzed the relevant CNV but did not discuss their results in an evolutionary context, such as what are the consequences of higher copy number. What hypothesis can be made concerning the complete loss of AMY2B locus observed in CanFam3.1 in comparison with the GSD genome? Is it related to the breed creation (Boxer vs GSD), or just a polymorphic CNV in modern breeds without any particular selection in current times? For example, it could be very interesting to see if, using your genome reference, you can use the GSD genomes already published and available on SRA and other related breeds to: 1) confirm your observations on this locus, and 2) explore breeds that you cite in your discussion (L490-496), checking how many copies each has on this locus. This will provide insight regarding about the evolutionary position of the GSD as regards the two cited papers L493-494 (Bigi et al 2015, Parker et al, 2017).
5) This comment follows my first comment and is maybe beyond this paper: I noticed the first author released the Dingo genome (Canis lupus dingo), submitted on NCBI in June 2018 (Bioproject: PRJNA477859) and already available on Ensembl. I did not find an associated paper to this work, so I am wondering if authors could make a comparison between both Dingo and GSD in this paper, highlighting potential genomic differences which would support the last sentence of your abstract: "This resource will enable further research related to canine diseases, the evolutionary relationships of canids, and other aspects of canid biology".
I noticed some typos:
L98: "best-known"
L186: Pancreatic amylase (AMY2B) analysis
L221-223: For a better understanding, I recommend your write this sentence like this:
"Alignment results confirmed the presence of seven repeat units, showing a perfect alignment to the seven-copy sequence construct (Figure 2A), but a "deletion" of one repeat unit relative to the eight-copy construct (Figure 2B)."
L290-292: Can you re-write this sentence please - I do not understand what is largely methylated and largely unmethylated?
L297-299: you should specify "in other models" because these references did not work on dogs.
L315: Please correct " Saphyr instrument"
L465: remove uppercase for "annotation".
Supplementary Table 1: typo in title: "statistics" when you open the file.

Methods

Are the methods appropriate to the aims of the study, are they well described, and are necessary controls included? Choose an item.

Conclusions

Are the conclusions adequately supported by the data shown? Choose an item.

Reporting Standards

Does the manuscript adhere to the journal's guidelines on minimum standards of reporting? Choose an item.

Choose an item.

Statistics

Are you able to assess all statistics in the manuscript, including the appropriateness of statistical tests used? Choose an item.

Quality of Written English

Please indicate the quality of language in the manuscript: Choose an item.

Declaration of Competing Interests

Please complete a declaration of competing interests, considering the following questions:

- Have you in the past five years received reimbursements, fees, funding, or salary from an organisation that may in any way gain or lose financially from the publication of this manuscript, either now or in the future?
- Do you hold any stocks or shares in an organisation that may in any way gain or lose financially from the publication of this manuscript, either now or in the future?
- Do you hold or are you currently applying for any patents relating to the content of the manuscript?
- Have you received reimbursements, fees, funding, or salary from an organization that holds or has applied for patents relating to the content of the manuscript?
- Do you have any other financial competing interests?
- Do you have any non-financial competing interests in relation to this paper?

If you can answer no to all of the above, write 'I declare that I have no competing interests' below. If your reply is yes to any, please give details below.

I declare that I have no competing interests

I agree to the open peer review policy of the journal. I understand that my name will be included on my report to the authors and, if the manuscript is accepted for publication, my named report including any attachments I upload will be posted on the website along with the authors' responses. I agree for my report to be made available under an Open Access Creative Commons CC-BY license (http://creativecommons.org/licenses/by/4.0/). I understand that any comments which I do not wish to

be included in my named report can be included as confidential comments to the editors, which will not be published.

Choose an item.

To further support our reviewers, we have joined with Publons, where you can gain additional credit to further highlight your hard work (see: https://publons.com/journal/530/gigascience). On publication of this paper, your review will be automatically added to Publons, you can then choose whether or not to claim your Publons credit. I understand this statement.

Yes Choose an item.
