# Supplementary material for: Canfam_GSD: De novo chromosome-length genome assembly of the German Shepherd Dog (Canis lupus familiaris) using a combination of long reads, optical mapping, and Hi-C
Source: Gigascience. 2020 Apr 1;9(4):giaa027. doi: 10.1093/gigascience/giaa027 (PMC7111595; doi:10.1093/gigascience/giaa027)
Supplement: giaa027_Reviewer_2_Report_Original_Submission — Klaus-Peter Koepfli, PhD -- 12/17/2019 Reviewed [file giaa027_reviewer_2_report_original_submission.pdf]

Reviewer Report

Title: Canfam_GSD : De novo chromosome-length genome assembly of the German Shepherd Dog (Canis lupus familiaris) using a combination of long reads, optical mapping and Hi-C

Version: Original Submission **Date:** 12/17/2019

Reviewer name: Klaus-Peter Koepfli, PhD

Reviewer Comments to Author:

General comments
The authors report on the de novo genome assembly and annotation of a female German shepherd, a popular and well-known breed of domestic dog. The chromosome-length genome was generated using a combination of sequences obtained from PacBio and ONT long reads,10X Genomics Chromium libraries, Bionano Genomics optical maps and Hi-C libraries. The assembly was produced using an iterative workflow in which the long-read data were first assembled into contigs and these were then successively scaffolded using the 10X Genomics Chromium data, optical maps and Hi-C data. Each stage of the assembly was evaluated in terms of continuity and completeness as judged by BUSCO scores. The hybrid assembly was subjected to three rounds of rigorous polishing to produce an accurate and highly continuous assembly. In fact, the German shepherd dog assembly is 80X more contiguous than the standard domestic dog reference genome, CanFam version 3.1 (based on the boxer breed). As such, the newly reported assembly achieves an important benchmark in terms of genome assembly quality.
Specific comments
The manuscript is generally well written and the methods of the genome sequencing and assembly are thoroughly presented in detail in both the main text and the Supplementary Materials. The newly reported assembly represents one of the most contiguous genome assemblies yet generated for a mammalian species outside of humans and will no doubt provide an important resource for countless studies in addition to the CanFam version 3.1 assembly which has been the primary assembly for many years. The in-depth annotation and analysis of the AMY2B copy number is especially thorough and well done. The paper will no doubt stimulate great interest by researchers in mammalian genomics as well as the general public. I recommend the manuscript be accepted for publication once the authors have addressed specific comments that I think would improve the clarity and impact of the manuscript, as described below.
1. There are no page numbers or line numbers, which makes it unnecessarily difficult to provide comments on the manuscript. Therefore, I will reference my comments according to the page number and manuscript section based on the pdf version of the manuscript. Please add page numbers and line numbers in future versions of the manuscript.
2. In the provided manuscript, the Methods section was placed before the Discussion section. As this has been submitted as a Research article, the Methods section should be moved to after the Discussion section.
3. Contig assembly, scaffolding and polishing were evaluated using N50 and BUSCO scores. However, I recommend the authors also assess the German Shepherd dog assembly using the K-mer Analysis

Toolkit (KAT; Mapleson et al. 2017 Bioinformatics 33: 574-576 and https://github.com/TGAC/KAT). KAT uses k-mer frequencies to profile errors, GC-bias, and other metrics along with providing quality control checking of assemblies at different stages. This would involve using the PacBio and ONT long reads as well as the de-barcoded raw reads from the 10X Genomics Chromium sequencing that were generated.
4. Title: I suggest revising the title of the manuscript so that it is less generic and more descriptive of the methods/technologies used:
"De novo chromosome-length genome assembly of the German Shepherd Dog (Canis lupus familiaris) using a combination of long reads, optical mapping and Hi-C"
5. Page 8, Results, Workflow: "Contigs were assembled using SMRT and ONT sequencing and then polished to minimize error propagation." Although the authors refer to Supplementary Figure 1 in this paragraph, for this sentence, the authors should reference the program(s) used for polishing and also the section of the Methods where this is detailed.
6. Page 9, Results, Assembly stats / completeness: "…against Laurasiatheria_ob9 (n=6,253)…" This should be revised as "…against the Laurasiatheria_ob9 data set (n=6,253)…"
7. Page 10, Table 1: In column three (CanFam3.1) and the row, "BUSCO complete (genome)", the value 91.10.8% single copy doesn't make sense. I assume the authors mean 91.1%. Please correct.
8. Page 10, Results: "Based on the existing CanFam3.1 annotation and the GSD annotation provided by GeMoMa…" Please provide a reference for the GeMoMa software.
9. Page 11, Results: "Pacreatic amylase (AMY2B) analysis" should be corrected as "Pancreatic amylase (AMY2B) analysis"
10. Page 11, Results, Pancreatic amylase (AMY2B) analysis: "The longest read in the region covered three plus copies…" This description of the number of copies is vague. The following revision is suggested: "The longest read in the region covered between three to [insert maximum number observed] copies…"
11. Page 11, Results, Pancreatic amylase (AMY2B) analysis: "Further examination of this region was attempted using both the Bionano genome map…" Suggested revision: "Further examination of this region was attempted using both the Bionano optical map…"
12. Page 13, Methods, Sampling: Nala the German Shepherd Dog: "Nala had a combined hip score of 3 (1 on LHS and 2 on RHS) when the x-ray was taken at 5 years of age…" Not all readers may understand the LHS and RHS notation. Suggested revision: "Nala had a combined hip score of 3 (1 on the left hand side and 2 on the right hand side) when the x-ray was taken at 5 years of age…"
13. Page 14, Methods, Pacific Bioscience Single Molecule Real-Time (SMRT) sequencing: "…and molecular integrity was assessed using pulse-field gel electrophoresis." In the interests of repeatability, the authors should provide more details about the PFGE experiment(s) including the instrument used (if commercial instrument), gel type and concentration, the voltage, run time, and the amount of DNA run and DNA standards used.
14. Page 14, Methods, Pacific Bioscience Single Molecule Real-Time (SMRT) sequencing: "…and at the Arizona Genomic Institute, University of Arizona (four SMRT cells with a 11Gb of data: NOTE: short read lengths were due to DNA shearing of the DNA during shipping from Australia to Arizona)." Suggested revision: "…and at the Arizona Genomic Institute, University of Arizona (four SMRT cells with a total of 11Gb of data; NOTE: short read lengths were due to DNA shearing of the DNA during shipping from Australia to Arizona)."

15. Page 15, Methods, 10X Genomics Chromium sequencing: The authors should reference Supplementary File 3 at the end of this paragraph, which provides a more detailed description of the methods used for the 10X Genomics Chromium sequencing. Also, Supplementary File 3 should be retitled as "10X Genomics Chromium sequencing: Detailed Methods."
16. Page 15, Methods, 10X Genomics Chromium sequencing: "…was barcoded from high-molecular-weight DNA according to manufacturers recommended protocols." Suggested revision: "…was barcoded from high-molecular-weight DNA according to the manufacturer's recommended protocols." Also, the authors should provide a reference for these protocols (i.e., a manual document or website URL).
17. Page 15, Methods, 10X Genomics Chromium sequencing: "QC was performed using LabChip GX and Qubit." Please provide manufacturer information and location for both of these instruments.
18. Page 15, Methods, Methylome: "…using MethylSeekR algorithm [27]." Suggested revision: "…using the MethylSeekR algorithm [27]."
19. Page 16, Methods, Bionano optical mapping: "Multiple cycles were performed to reach average raw genome depth of coverage of 190X." Suggested revision: "Multiple cycles were performed to reach an average raw genome depth of coverage of 190X."
20. Page 17, Methods, 10X Chromium linked-reads: "The arrow polished SMRT/ONT assembly was scaffolded…" Suggested revision: "The Arrow-polished SMRT/ONT assembly was scaffolded…"
21. Page 17, Methods, 10X Chromium linked-reads: "The 10X data was aligned using the linked-read analysis software provided by 10X Genomics, Long Ranger, v2.1.6 (https://www.10xgenomics.com/), misaligned reads and reads not mapping to contig ends were removed, all possible connections between contigs were computed keeping best reciprocal connections." This sentence is overly complicated and doesn't make sense as written. I suggest breaking this into two sentences and revising as follows: "The 10X read data was aligned using the Long Ranger, v2.1.6 software (https://www.10xgenomics.com/). Misaligned reads and reads not mapping to contig ends were removed, and all possible connections between contigs were computed by keeping the best reciprocal connections."
22. Page 18, Methods, Optical mapping for super-scaffolding using Bionano data: "Alignments indicating conflict between the sequence and optical maps, and hence suggestive of mis-assembly, were evaluated such that, conflicts supported by single-molecule optical maps (thus supporting optical map) would cause the sequence map to be "cut" at the conflict point, else the optical map was "cut"." This meaning and method described in the second part of this sentence is not clear (after "were evaluated such that"). Please revise to improve clarity.
23. Page 19, Methods, Gap filling: "After scaffolding and correction, all raw reads were aligned to the assembly…" Which raw reads are the authors referring to here? The raw reads from the 10X Genomics Chromium sequencing? Please specify.
24. Page 20, Methods, Polishing Round 2: "…and correcting the single nucleotide polymorphism's (SNP) and indels using Pilon [43]." Suggested revision: "…and correcting the single nucleotide polymorphisms (SNPs) and indels using Pilon [43]."
25. Page 20, Methods, Low-coverage filter: "Any scaffolds with median coverage less than 3 (e.g., less than 50% of the scaffold covered by at least three reads) were filtered as Low Coverage." To improve clarity, I suggest the following revision: "Any scaffolds with median coverage less than 3 (e.g., less than 50% of the scaffold covered by at least three reads) were filtered out as Low Coverage scaffolds."
26. Pages 20-21, Methods, Purge Haplotigs analysis - round 1 and round 2: "Subreads were re-mapped

on to the remaining 837 scaffolds…" and "Subreads were re-mapped on to the remaining 558 scaffolds…" It's not clear where these remaining scaffolds originate from in the two paragraphs about purging of the haplotigs. Are these the scaffolds remaining after primary assembly using the PacBio+ONT+10X Genomics Chromium+Bionano optical map+Hi-C? Also, after the first round of purging, it seems only 279 scaffolds were processed, leaving 558 scaffolds to be processed in the second round. Is this correct? The authors need to better clarify these points so that they are more understandable for readers.

27. Page 21, Methods, Purge Haplotigs analysis - round 2: "A single remaining Scaffold marked as JUNK…" Scaffold in this sentence does not need to be capitalized.

28. Page 21, Methods, CanFam3.1 Chromosome Mapping: The PAFScaff v0.2.0 program - please provide a reference or URL for this software.

29. Pages 22-23, Methods, Gene prediction including Annotation of repetitive elements: Please provide NCBI accession numbers for the assembly/annotation of the nine species used for the homology-based gene prediction analyses.

30. Page 23, Discussion: "…commonly registered KC breeds…" Please spell out "KC" when used for the first time. I assume the authors mean Kennel Club here.

31. Page 23, Discussion: I suggest the authors expand on the discussion in the first paragraph of the Discussion section by describing in a few sentences how the German shepherd dog genome assembly can be applied "for advancing knowledge of breed specific diseases." As currently written, the paragraph only provides a description of diseases that German shepherds are pre-disposed to. Surely, the genetic etiology of some of these conditions/diseases has been previously researched. The authors should reference some of these studies and then detail how the high-quality reference genome they have generated will advance such research.

Methods

Are the methods appropriate to the aims of the study, are they well described, and are necessary controls included? Choose an item.

Conclusions

Are the conclusions adequately supported by the data shown? Choose an item.

Reporting Standards

Does the manuscript adhere to the journal's guidelines on minimum standards of reporting? Choose an item.

Choose an item.

Statistics

Are you able to assess all statistics in the manuscript, including the appropriateness of statistical tests used? Choose an item.

Quality of Written English

Please indicate the quality of language in the manuscript: Choose an item.

Declaration of Competing Interests

Please complete a declaration of competing interests, considering the following questions:

- Have you in the past five years received reimbursements, fees, funding, or salary from an organisation that may in any way gain or lose financially from the publication of this manuscript, either now or in the future?
- Do you hold any stocks or shares in an organisation that may in any way gain or lose financially from the publication of this manuscript, either now or in the future?
- Do you hold or are you currently applying for any patents relating to the content of the manuscript?
- Have you received reimbursements, fees, funding, or salary from an organization that holds or has applied for patents relating to the content of the manuscript?
- Do you have any other financial competing interests?
- Do you have any non-financial competing interests in relation to this paper?

If you can answer no to all of the above, write 'I declare that I have no competing interests' below. If your reply is yes to any, please give details below.

I declare that I have no competing interests.

I agree to the open peer review policy of the journal. I understand that my name will be included on my report to the authors and, if the manuscript is accepted for publication, my named report including any attachments I upload will be posted on the website along with the authors' responses. I agree for my report to be made available under an Open Access Creative Commons CC-BY license (http://creativecommons.org/licenses/by/4.0/). I understand that any comments which I do not wish to be included in my named report can be included as confidential comments to the editors, which will not be published.

Choose an item.

To further support our reviewers, we have joined with Publons, where you can gain additional credit to further highlight your hard work (see: https://publons.com/journal/530/gigascience). On publication of this paper, your review will be automatically added to Publons, you can then choose whether or not to claim your Publons credit. I understand this statement.

Yes Choose an item.
