# Supplementary material for: Canfam_GSD: De novo chromosome-length genome assembly of the German Shepherd Dog (Canis lupus familiaris) using a combination of long reads, optical mapping, and Hi-C
Source: Gigascience. 2020 Apr 1;9(4):giaa027. doi: 10.1093/gigascience/giaa027 (PMC7111595; doi:10.1093/gigascience/giaa027)
Supplement: giaa027_Reviewer_2_Report_Revision_1 — Klaus-Peter Koepfli, PhD -- 2/1/2020 Reviewed [file giaa027_reviewer_2_report_revision_1.pdf]

Reviewer Report

Title: Canfam_GSD : De novo chromosome-length genome assembly of the German Shepherd Dog (Canis lupus familiaris) using a combination of long reads, optical mapping and Hi-C

Version: Revision 1 **Date:** 2/1/2020

Reviewer name: Klaus-Peter Koepfli, PhD

Reviewer Comments to Author:

I have read the revised manuscript by Field et al. and find that it has been much improved compared to the original submission. The authors have done an outstanding job in providing thorough responses to my comments (and those from the other reviewer) and have revised the manuscript accordingly. The revisions have clarified specific sections that were previously opaque and the added text has nicely expanded upon certain themes related to German shepherd dog breeds. The Canfam_GSD assembly establishes an important benchmark in de novo genome assembly and will no doubt be an invaluable resource to the canine genomics community.
There are just a few minor errors in some of the new text the authors have added to the revised manuscript that need correction. These corrections can be easily made in the proofs of the manuscript.
Line 2: Make sure to italicize Canis lupus familiaris in the title.
Line 297: "underlining genes" should be changed to "underlying genes"
Line 378: "Protocol used..." Revise as "The protocol used..."
I recommend the manuscript be accepted for publication.

Methods

Are the methods appropriate to the aims of the study, are they well described, and are necessary controls included? Choose an item.

Conclusions

Are the conclusions adequately supported by the data shown? Choose an item.

Reporting Standards

Does the manuscript adhere to the journal's guidelines on [minimum standards of reporting?](minimum standards of reporting) Choose an item.

Choose an item.

Statistics

Are you able to assess all statistics in the manuscript, including the appropriateness of statistical tests used? Choose an item.

Quality of Written English

Please indicate the quality of language in the manuscript: Choose an item.

Declaration of Competing Interests

Please complete a declaration of competing interests, considering the following questions:

- Have you in the past five years received reimbursements, fees, funding, or salary from an organisation that may in any way gain or lose financially from the publication of this manuscript, either now or in the future?
- Do you hold any stocks or shares in an organisation that may in any way gain or lose financially from the publication of this manuscript, either now or in the future?
- Do you hold or are you currently applying for any patents relating to the content of the manuscript?
- Have you received reimbursements, fees, funding, or salary from an organization that holds or has applied for patents relating to the content of the manuscript?
- Do you have any other financial competing interests?
- Do you have any non-financial competing interests in relation to this paper?

If you can answer no to all of the above, write 'I declare that I have no competing interests' below. If your reply is yes to any, please give details below.

I declare that I have no competing interests.

I agree to the open peer review policy of the journal. I understand that my name will be included on my report to the authors and, if the manuscript is accepted for publication, my named report including any attachments I upload will be posted on the website along with the authors' responses. I agree for my report to be made available under an Open Access Creative Commons CC-BY license (http://creativecommons.org/licenses/by/4.0/). I understand that any comments which I do not wish to be included in my named report can be included as confidential comments to the editors, which will not be published.

Choose an item.

To further support our reviewers, we have joined with Publons, where you can gain additional credit to further highlight your hard work (see: https://publons.com/journal/530/gigascience). On publication of this paper, your review will be automatically added to Publons, you can then choose whether or not to claim your Publons credit. I understand this statement.

Yes Choose an item.
